# Quantifying cause-related mortality in Australia, incorporating multiple causes: observed patterns, trends and practical considerations

**DOI:** 10.1093/ije/dyac167

**Published:** 2022-08-19

**Authors:** Karen Bishop, Margarita Moreno-Betancur, Saliu Balogun, James Eynstone-Hinkins, Lauren Moran, Chalapati Rao, Emily Banks, Rosemary J Korda, Michelle Gourley, Grace Joshy

**Affiliations:** National Centre for Epidemiology and Population Health, Australian National University, Canberra, ACT, Australia; Clinical Epidemiology and Biostatistics Unit, Murdoch Children’s Research Institute, Melbourne, VIC, Australia; Department of Paediatrics, University of Melbourne, Melbourne, VIC, Australia; National Centre for Epidemiology and Population Health, Australian National University, Canberra, ACT, Australia; Health and Vital Statistics Section, Australian Bureau of Statistics, Canberra, ACT, Australia; Health and Vital Statistics Section, Australian Bureau of Statistics, Canberra, ACT, Australia; National Centre for Epidemiology and Population Health, Australian National University, Canberra, ACT, Australia; National Centre for Epidemiology and Population Health, Australian National University, Canberra, ACT, Australia; National Centre for Epidemiology and Population Health, Australian National University, Canberra, ACT, Australia; Population Health Group, Australian Institute of Health and Welfare, Canberra, ACT, Australia; National Centre for Epidemiology and Population Health, Australian National University, Canberra, ACT, Australia

**Keywords:** Mortality, multiple causes of death, chronic diseases, risk factors, comorbidity, population health

## Abstract

**Background:**

Mortality statistics using a single underlying cause of death (UC) are key health indicators. Rising multimorbidity and chronic disease mean that deaths increasingly involve multiple conditions. However, additional causes reported on death certificates are rarely integrated into mortality indicators, partly due to complexities in data and methods. This study aimed to assess trends and patterns in cause-related mortality in Australia, integrating multiple causes (MC) of death.

**Methods:**

Deaths (*n *= 1 773 399) in Australia (2006–17) were mapped to 136 ICD-10-based groups and MC indicators applied. Age-standardized cause-related rates (deaths/100 000) based on the UC (ASR_UC_) were compared with rates based on any mention of the cause (ASR_AM_) using rate ratios (RR = ASR_AM_/ASR_UC_) and to rates based on weighting multiple contributing causes (ASR_W_).

**Results:**

Deaths involved on average 3.4 causes in 2017; the percentage with >4 causes increased from 20.9 (2006) to 24.4 (2017). Ischaemic heart disease (ASR_UC_ = 73.3, ASR_AM_ = 135.8, ASR_W_ = 63.5), dementia (ASR_UC_ = 51.1, ASR_AM_ = 98.1, ASR_W_ = 52.1) and cerebrovascular diseases (ASR_UC_ = 39.9, ASR_AM_ = 76.7, ASR_W_ = 33.5) ranked as leading causes by all methods. Causes with high RR included hypertension (ASR_UC_ = 2.2, RR = 35.5), atrial fibrillation (ASR_UC_ = 8.0, RR = 6.5) and diabetes (ASR_UC_ = 18.5, RR = 3.5); the corresponding ASR_W_ were 12.5, 12.6 and 24.0, respectively. Renal failure, atrial fibrillation and hypertension ranked among the 10 leading causes by ASR_AM_ and ASR_W_ but not by ASR_UC_. Practical considerations in working with MC data are discussed.

**Conclusions:**

Despite the similarities in leading causes under the three methods, with integration of MC several preventable diseases emerged as leading causes. MC analyses offer a richer additional perspective for population health monitoring and policy development.

Key Messages

- Increasingly, death is the result of multiple contributing causes, but mortality statistics usually rely on a single underlying cause. This study presents a whole-of-population analysis of Australian multiple cause of death data, measuring mortality for 136 specific causes.
- Mortality indicators using multiple causes of death highlight health conditions that contribute considerably to death but are less likely to feature as the underlying cause. Recent multiple-cause weighting methods enable a principled estimation of cause-related mortality rates integrating the contribution of risk factors and comorbid chronic diseases.
- For application of multiple-cause weighting methods, careful consideration is required around the weighting strategy, the cause list and the handling of ill-defined causes. Methodological standards in analysis and reporting would enhance comparability between studies.
- Our results provide an in-depth population-level examination of cause-related mortality in Australia, incorporating multiple causes by various methods. The comprehensive findings offer further insight into the contribution to mortality from potentially preventable health conditions.

## Background

Worldwide, deaths are occurring at increasingly older ages and are largely due to chronic diseases. Recent global statistics underscore this health burden: 73% of all deaths are due to non-communicable diseases, with 71% from four main causes—cardiovascular diseases, chronic respiratory diseases, cancer and diabetes.^1^ Substantial increases in deaths due to diabetes, renal failure and dementia have occurred since 2000^2^^,^^3^ and rapid rises are forecast for chronic obstructive pulmonary disease (COPD), diabetes, chronic kidney disease, dementia and lung cancer.^4^ In this context of increasing prevalence of multimorbid chronic diseases in older age groups, ascribing causality of death to a single disease obscures the complex pathological involvement of contributing causes.^5–7^

Yet, mortality statistics are typically based on a single underlying cause of death (UC) selected from multiple causes of death (MC) reported on the death certificate. The UC is an established construct selected to reflect the disease or condition that initiated the main morbid process and is a targeted point for public health intervention: its embedded use is important for measuring population health and for monitoring progress. However, the information on the certificate on the remaining causes is largely unused, despite its potential value in quantifying the genuine contributions to death of MC.^5^^,^^8^^,^^9^ Indeed, deaths due to natural causes in Italy and France (2003), and in Australia (2007), involved on average 4.0, 3.1 and 3.1 health conditions, respectively; in the USA (2003–16) and Australia (2018), 30% and 24% of deaths, respectively, had four or more causes.^10–12^

Mortality indicators that incorporate MC have been proposed,^5–7^^,^^13^^,^^14^ enabling the use of data on all causes in each death, that is the UC and other causes reported in Part I (the main morbid process, typically consequences and complications of the UC) and Part II (theoretically significant contributing conditions) of a standard medical death certificate. The availability of this information provides many options for studying the drivers of mortality, but it also raises some methodological challenges that may explain why these indicators are yet to be comprehensively assessed and reported in many countries. For instance, MC summary measures commonly consider whether the cause of interest is mentioned anywhere on the death certificate when estimating cause-related mortality rates^5^^,^^11^^,^^12^^,^^15^^,^^16^ (Supplementary File 1, available as Supplementary data at *IJE* online). We refer to this as the ‘any-mention’ approach. Although simple to apply, this approach may provide a misleading view of population-level mortality patterns, as cause-related rates calculated this way do not add up to the total overall (all-cause) mortality; each death is counted as many times as there are causes. To circumvent this issue, a MC-weighting approach was recently proposed that conceptualizes death as the outcome of a combination of conditions.^6^^,^^7^

Another consideration in integrating MC data in indicators is whether all the causes in each death are used. Assessment of all the conditions associated with the UC is important for monitoring population health. Alternatively, indicators could be computed using the UC and causes in Part II of the death certificate only,^6^ referred to as contributing causes (CC), capturing conditions which are typically comorbid with or antecedent to the underlying cause, including relevant previous illness or significantly contributing risk factors. This approach ignores the consequences and complications of the UC, emphasizing causal pathways theoretically independent or antecedent to the main morbid process.

There has been no previous attempt to quantify cause-related mortality in Australia comprehensively, incorporating MC. This study aimed to provide an in-depth, population-level examination of cause-related mortality in Australia, assessing trends in MC indicators over time and comparing UC-based rates with those based on any-mention and MC-weighting approaches that consider the location of causes on the death certificate.^6^^,^^7^ This study also highlights practical considerations for application of MC methods to administrative death registration data, which could be used when applying these methods in other settings.

## Methods

### Study population and data

Individual-level data for all deaths in Australia during 2006–17 were used, including age at death, registration year and the causes of death. In Australia the latter are available in two formats: ‘entity axis’ data contains contributing causes (CC) as reported in Part II of the death certificate, and the ‘record axis’ data comprises a single UC and up to 19 associated causes (AC) generated by application of the International Classification of Diseases 10th Revision (ICD-10) coding and by processing rules to all causes reported on the death certificate (Parts I and II) (Supplementary File 2, available as Supplementary data at *IJE* online). We used record axis data, except for rates based on weighting where the CC were extracted from the entity axis. Death records with missing age at death were excluded (<0.01%).

We constructed 136 mutually exclusive and exhaustive groups of health conditions based on existing cause of death categories^17–20^ and expert opinion (Supplementary File 2). The derived list included commonly monitored causes of death and usually non-fatal health conditions, and allowed aggregation to 19 major groups. All causes in the record and entity axes were mapped to the list. Death records with an ill-defined UC were excluded.

### Statistical methods

Using all mentions of causes in the record axis, we described the proportions of deaths by number of causes, and the average number of causes per death for each year (2006–17). For each cause group, we estimated cause-related, age-standardized mortality rates (ASR, as deaths/100 000) based on underlying causes only (ASR_UC_), and by the ‘any-mention’ method (ASR_AM_), which considers each death that mentions the cause of interest either as UC or as an associated cause. Using these rates, we calculated the rate ratio (ASR_AM_/ASR_UC_), also known as the standardized ratio of multiple to underlying cause (SRMU), to indicate the extent to which each cause is selected as associated relative to underlying cause.^5^^,^^21–23^ An SRMU = 1 indicates that the cause under consideration is always the UC; SRMU = 2 indicates equal representation in underlying and associated causes; SRMU > 2 indicates that the cause is more often selected as an associated than underlying cause. The reciprocal of the SRMU expressed as a percentage describes the proportion of the cause-related mortality that is indicated by the UC^14^^,^^22^ (Supplementary File 2).

Since cause-related rates by the any-mention method do not add to the total all-cause mortality rate, we applied an MC-weighting strategy^6^^,^^7^ to deaths registered over 2015–17. From previously applied MC-weighting methods (Supplementary File 1), we selected a primary MC-weighting strategy for this study, which ascribed 50% weight to the UC and apportioned the remaining 50% equally across the CC, so that the total sum of weights within each death is 100%. In deaths with a single cause, the UC received 100%. Thus, the cause-related rates (ASR_W_) estimated using the weighted causes of death add up to the all-cause mortality rate. Ill-defined causes and duplicate mentions (due to mapping to the same cause group) in the associated or contributing causes were not considered in deriving the weights. A hypothetical example of the weighting applied to causes on the death certificate is shown in Table 1. For sensitivity, we compared ASR_W_ with two additional weighting strategies (Supplementary File 2). MC-weighted rates (ASR_W_) were compared with ASR_UC_ using percentage changes and rate differences. Causes were ranked according to magnitude of ASR_UC_, ASR_AM_ and ASR_W_, and the 20 leading causes by each method were compared.

**Table 1:** Hypothetical example of application of multiple-cause weighting to causes selected as underlying and other causes according to location on death certificate

ICD-10 description (ICD-10 code)	Position (part/line)	Mapped to cause list	Inclusion criteria	Weighting strategy	
				UC only	UC 0.5
Pneumonia (J18.9)	I/a	Pneumonia	Exclude (Part I)	0.0	0.0
Lung cancer (C18.9)	I/b	Lung cancer	Include (Underlying)	1.0	0.5
Emphysema (J43.9)	II	COPD	Include (Contributing)	0.0	0.25
Atherosclerotic heart disease (I25.1)	II	Ischaemic heart disease	Include (Contributing)	0.0	0.25
Chronic ischaemic heart disease (I25.9)	II	Ischaemic heart disease	Exclude (Duplicate)	0.0	0.0
			Sum of weights	1.0	1.0

Considering the underlying and contributing causes, the average number of causes per death was 1.9.

UC, underlying cause of death; COPD, chronic obstructive pulmonary disease.

Throughout, rates were directly standardized to the 2011 Australian Estimated Resident Population in 5-year age groups (0–4 to 95+). All analysis was undertaken using SAS EG version 9.4.

## Results

The 1 773 525 deaths registered over 2006–17 were used for analysis. All-cause standardized mortality rates for these deaths declined from 696 to 602 (per 100 000) over this period, with most (80–82%) occurring at ages ≥65 (Table 2). Deaths with more than four causes increased from 20.9% in 2006 to 24.4% in 2017. The annual average number (causes/death) was 3.1–3.4, which varied by age group: lower in the oldest group (95+) compared with the 85–94-year age group. Notable increases in this measure during the study period occurred for coroner-certified deaths (from 2.7 to 3.8). Considering only the UC and CC (2015–17 combined), the average number was 1.9.

**Table 2:** Summary of deaths and multiple causes of death in Australia, 2006–2017

Year of registration	2006	2007	2008	2009	2010	2011	2012	2013	2014	2015	2016	2017
Deaths												
Total number deaths^a^	133 733	137 843	143 932	140 749	143 459	146 928	147 095	147 660	153 546	159 050	158 500	160 904
Rate (deaths/100 000)^b^	696	694	705	671	662	658	639	623	629	633	612	602
Male deaths (%)	51.3	51.2	51.1	51.4	51.2	51.3	50.8	51.3	51.0	51.1	51.6	51.5
Deaths at age 65 or more (%)	79.7	79.7	80.4	79.7	80.3	80.7	81.3	81.1	81.2	81.5	81.8	82.3
Coroner-certified deaths (%)	12.7	12.2	13.0	13.3	12.1	11.5	11.7	13.2	14.0	12.8	12.1	11.9
**Causes^c^**												
Total number of causes	426 009	433 638	467 062	448 320	454 323	465 638	465 563	490 560	513 826	535 636	532 886	539 841
Deaths (%) with												
1 cause	18.0	19.9	17.4	17.6	18.0	18.1	18.6	17.5	17.4	18.0	18.3	18.7
2 causes	22.6	21.9	21.8	22.5	22.4	22.4	22.2	21.4	21.1	21.1	21.1	21.1
3 causes	22.1	21.5	22.0	22.2	22.3	22.2	22.1	21.3	21.4	20.4	20.4	20.2
4 causes	16.4	16.2	16.8	17.0	16.9	16.9	16.8	16.3	16.1	16.1	15.8	15.5
>4 causes	20.9	20.6	22.0	20.7	20.3	20.5	20.3	23.6	24.0	24.5	24.5	24.4
Average number of causes per death												
Doctor-certified deaths	3.3	3.2	3.3	3.3	3.2	3.2	3.2	3.4	3.4	3.4	3.3	3.3
Coroner-certified deaths	2.7	2.7	2.8	2.7	2.7	2.7	2.8	3.1	3.3	3.4	3.5	3.8
All deaths	3.2	3.1	3.2	3.2	3.2	3.2	3.2	3.3	3.3	3.4	3.4	3.4

a Excludes 126 deaths with missing age.

b All-cause mortality rates are directly standardized to the Australian Estimated Resident Population 2011 by 5-year age groups (0–4 to 95+).

c ‘Causes’ refers to all causes in the death record extracted from the record axis.

**Figure 1:**
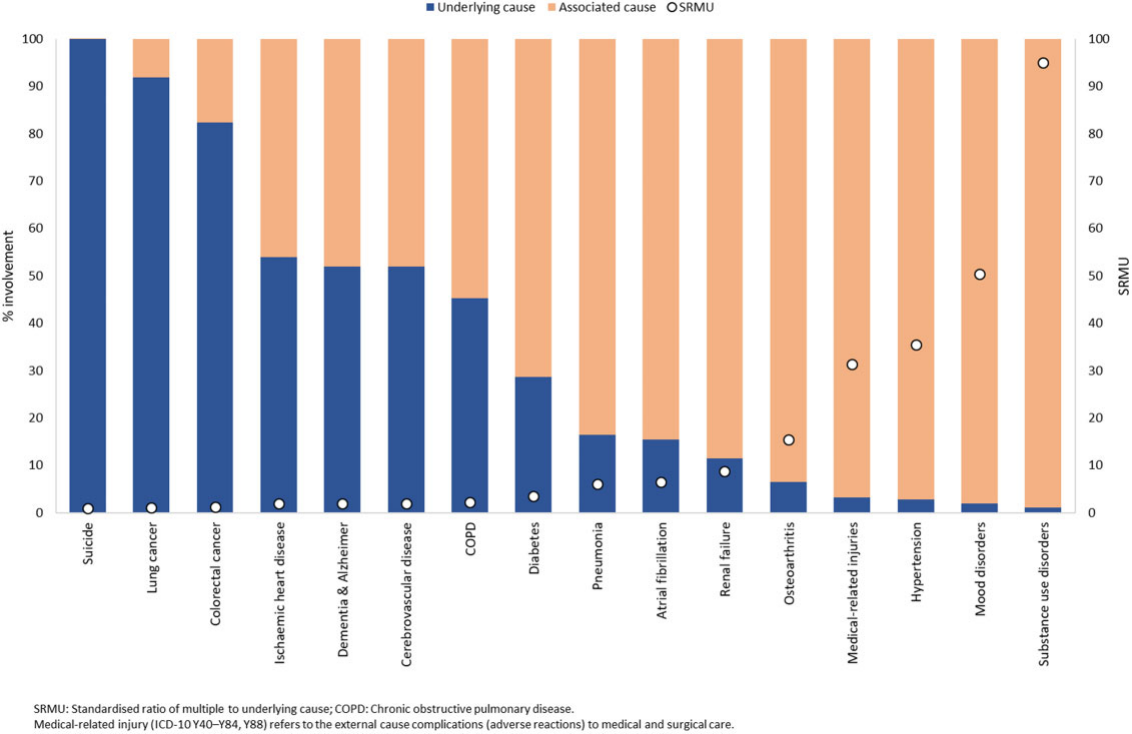
Per cent involvement as underlying and associated causes and SRMU, selected causes, Australia, 2015–17

Evaluation of the SRMU and the percent involvement represented by UC showed that external causes and cancers are typically the UC and occur less frequently among associated causes, and consequently have low SRMU (Figure 1). All cancer causes measured had SRMU < 2.0, ranging from 1.0 for pancreatic cancer to 1.4 for prostate cancer (Supplementary File 3, Supplementary Table S3.1, available as Supplementary data at *IJE* online). The SRMU of 1.9 for cerebrovascular diseases, ischaemic heart disease (IHD) and dementia indicated equivalent involvement as the UC (52–54%). With SRMU = 2.2, less than half the involvement of chronic obstructive pulmonary disease (COPD) (45%) is indicated by the UC. Several causes had high SRMU, indicating that they are more often associated rather than UC; these included diabetes (SRMU = 3.5), metabolic disorders (5.5), atrial fibrillation (6.5), renal failure (8.7) and hypertension (35.5). The highest SRMU were for substance use (95.0) and mood disorders (50.4), manifested by their involvement almost entirely as associated causes (99% and 98%, respectively).

With MC-weighting, rates (ASR_W_) increased relative to those by UC for musculoskeletal (by 75%), mental (139%), genitourinary (55%), blood (50%) and endocrine (34%) conditions. With MC-weighting, rates decreased for cancers (by 12%) and infections (15%), with little or no change for cardiovascular, nervous system, respiratory and digestive diseases (Supplementary File 3, Supplementary Table S3.2, available as Supplementary data at *IJE* online).

Table 3 shows a comparison of ranking and magnitude of leading causes during 2015–17, according to underlying cause, any mention and MC-weighting. Ischaemic heart disease (IHD), dementia and cerebrovascular diseases were consistently the three leading causes under all methods. SRMU nearing 2.0 shows that these causes were involved in substantially more deaths than recognized by the UC alone; for example, IHD was the UC in 57 685 (12.3%) deaths but mentioned in 106 547 (22.7%) deaths. Diabetes and COPD ranked 8th or higher under each method. With MC-weighting, the ranking of renal failure and atrial fibrillation rose from 17th and 18th positions by ASR_UC_ to 8th and 9th, respectively, by ASR_W_. Consideration of MC analysis revealed the contribution to mortality of hypertension as well; hypertension did not feature among leading UC but emerged as a leading cause by ASR_AM_ and ASR_W_ by ranking 4th and 10th, respectively. On the other hand, MC-weighting diminished rankings for suicide (from 9th by UC to 19th by MC) and fell (from 15th by UC to 20th by MC). The MC-weighting method had less effect on the rankings of site-specific cancers, probably due to their propensity to be recorded as underlying causes, as indicated by the SRMU analysis, and to be reported alone or with no other CC in Part II.

**Table 3:** Leading causes of death by underlying cause, any-mention and multiple-cause weighting methods, Australia, 2015–17

Leading underlying cause				Leading ‘any-mention’				Leading weighted cause			
Cause	No. deaths	% deaths	ASR_UC_	Cause	No. causes	% deaths	ASR_AM_	Cause	No. deaths	% deaths	ASR_W_
1. Ischaemic heart disease	57 685	12.3	73.3	1. Ischaemic heart disease	106 547	22.7	135.8	1. Ischaemic heart disease	49 886	10.6	63.5
2. Dementia and Alzheimer	40 853	8.7	51.1	2. Dementia and Alzheimer	78 187	16.6	98.1	2. Dementia and Alzheimer	41 673	8.9	52.1
3. Cerebrovascular disease	31 521	6.7	39.9	3. Cerebrovascular disease	60 415	12.9	76.7	3. Cerebrovascular disease	26 393	5.6	33.5
4. Lung cancer	25 142	5.4	32.8	4. Hypertension	60 298	12.8	76.4	4. COPD	21 887	4.7	28.2
5. COPD	21 938	4.7	28.2	5. Renal failure	57 767	12.3	73.6	5. Lung cancer	20 774	4.4	27.1
6. Colorectal cancer	16 339	3.5	21.3	6. Pneumonia	51 877	11.0	65.6	6. Diabetes	18 589	4.0	24.0
7. Diabetes	14 310	3.0	18.5	7. Diabetes	49 871	10.6	64.4	7. Colorectal cancer	14 442	3.1	18.8
8. Other blood cancers	10 549	2.2	13.7	8. COPD	48 397	10.3	62.3	8. Renal failure	12 120	2.6	15.4
9. Suicide	9132	1.9	12.6	9. Atrial fibrillation	41 172	8.8	52.0	9. Atrial fibrillation	10 011	2.1	12.6
10. Prostate cancer	9722	2.1	12.5	10. Heart failure (specified)	39 498	8.4	49.6	10. Hypertension	9897	2.1	12.5
11. Breast cancer	8901	1.9	11.7	11. Septicaemia	28 162	6.0	36.2	11. Other blood cancers	9402	2.0	12.2
12. Pancreatic cancer	8671	1.8	11.3	12. Lung cancer	27 392	5.8	35.7	12. Prostate cancer	9107	1.9	11.7
13. Pneumonia	8666	1.8	10.8	13. Other heart diseases	26 912	5.7	34.7	13. Breast cancer	8469	1.8	11.1
14. Cancer unknown primary	8127	1.7	10.5	14. Cancer secondary site	23 540	5.0	30.8	14. Pneumonia	7783	1.7	9.7
15. Falls	8115	1.7	10.3	15. Pneumonitis	22 625	4.8	28.9	15. Pancreatic cancer	7418	1.6	9.7
16. Other heart diseases	6806	1.4	8.8	16. Residual injuries	19 700	4.2	26.5	16. Heart failure (specified)	7388	1.6	9.2
17. Renal failure	6708	1.4	8.5	17. Colorectal cancer	19 925	4.2	25.9	17. Other heart diseases	6980	1.5	9.0
18. Atrial fibrillation	6413	1.4	8.0	18. Artery diseases	19 290	4.1	24.6	18. Cancer unknown primary	6779	1.4	8.8
19. Liver cancer	5600	1.2	7.4	19. Medical-related injuries	15 171	3.2	19.7	19. Suicide	5850	1.2	8.1
20. Artery diseases	5621	1.2	7.2	20. Other blood cancers	14 934	3.2	19.3	20. Falls	6040	1.3	7.6
**Leading underlying causes**	**310** **819**	**66.2**	**398.3**					**Leading weighted causes**	**300** **890**	**64.1**	**384.7**
**All other underlying causes**	**158** **839**	**33.8**	**206.1**					**All other weighted causes**	**168** **768**	**35.9**	**219.6**
**Total (all causes)**	**469** **658**	**100.0**	**604.4**					**Total (weighted all causes)**	**469** **658**	**100.0**	**604.4**

Excludes 8796 (1.8%) deaths with ill-defined underlying cause. The number and percentage of causes under the any-mention method will not sum to totals as each death can be counted more than once. ‘Medical-related injuries’ (ICD-10 Y40–Y84, Y88) refers to the external cause complications (adverse reactions) to medical and surgical care. ‘Residual injuries’ refers to the ‘other’ category of consequences of external causes (see cause list for the ICD-10 codes captured here). Consequences of injuries are largely excluded from weighting due to the high proportion (86%) recorded as being in ‘Part I’ in the processing of causes of death.

COPD, chronic obstructive pulmonary disease; ASR, age standardized rate (deaths per 100 000) based on the underlying cause (ASR_UC_), any-mention (ASR_AM_) and weighted multiple causes (ASR_W_) methods. For weighted multiple causes, the underlying cause is weighted 50% and the remaining 50% apportioned equally to contributing causes.

**Figure 2:**
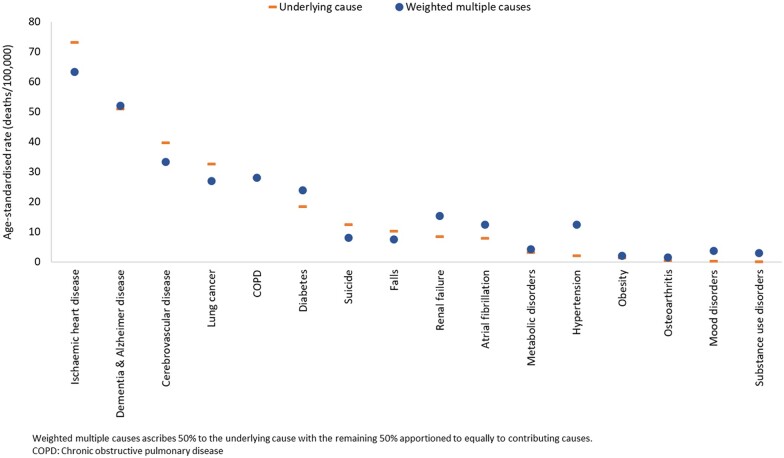
Age-standardized mortality rates for underlying cause and weighted multiple causes, selected causes, Australia, 2015–17

Compared with rates by UC, MC-weighted rates increased substantially for diabetes (by 30%), renal failure (82%), atrial fibrillation (57%), obesity (56%), metabolic disorders (37%) and hypertension (482%) (Figure 2). Examples of causes where rates decreased included IHD (by 13%) and cerebrovascular diseases (16%), suggesting 9.9 and 6.5 fewer deaths, respectively, per 100 000 than the ASR_UC_. The ASR_W_ did not change for dementia, COPD and Parkinson disease. Consistent rate declines were observed for all cancers, with the largest for lung cancer (17%), unknown primary cancer (17%) and liver cancer (16%). As MC-weighting preserves total death counts, the fractions of deaths ‘lost’ for lung cancer are ‘gained’ elsewhere according to the frequencies and combinations of other causes recorded for lung cancer deaths. Patterns were similar when rates based on MC-weighting methods were estimated using two alternative weighting approaches^6^^,^^7^^,^^24^ (Supplementary File 3, Supplementary Table S3.3, available as Supplementary data at *IJE* online).

Sensitivity analyses (results not shown) indicated that MC indicators are highly sensitive to the specificity of cause list used and the handling of ill-defined and duplicate mentions: key considerations when working with MC are outlined in Table 4.

**Table 4:** Key considerations when working with multiple causes of death

Key considerations in multiple cause analysis	
Specificity of cause list	Exhaustive and mutually exclusive to map all possible causes of death Specific enough to identify causes that are not usually the underlying cause, for example, hypertension Suitable for monitoring causes of interest in the local health context, e.g. specifying asthma and chronic obstructive pulmonary disease as separate causes Suitable for the research question; specific causes of interest may need to be grouped separately
Ill-defined and unknown causes	Consider ignoring deaths and their causes when the underlying cause is ill-defined, as these do not provide further insight into patterns of causes of death. Similarly, ill-defined causes could be excluded from the non-underlying causes. Alternatively, redistribution using a method suitable to multiple causes, or a method of multiple imputation, could be applied to maintain the deaths with an ill-defined underlying cause
Duplicate mentions of causes	Ignore duplicate mentions of the underlying and non-underlying causes to avoid overestimating their contribution to death
Weighting strategy and causes included (if relevant)	For population-level quantification of mortality, consider using the underlying cause as well as other contributing causes that were not part of the main morbid process (conditions in Part II of the death certificate, if these data are available). As the underlying cause of death is an internationally developed construct with a negotiated set of processes for identification, and remains critical for mortality statistics, it is reasonable to assign greater importance to the underlying cause such as assigning a weight of 50% to the UC and the remaining 50% equally distributed among the causes in Part II Both the objective of the study and data availability should determine the inclusion or otherwise of conditions from Parts I and II of the death certificate. For example, a study to assess end-of-life care needs associated with certain antecedent causes could consider the immediate causes of death, as might a study on the safety and quality of care, assessing the impact of sepsis-related mortality For quantification of mortality for a specific cause of interest, consider using non-arbitrary weighting. Ideally, the development of non-arbitrary weights will be the object of an international consensus needing significant expert input using Delphi style consultation. If weighting of causes based on position on the death certificate is not relevant for the research objective, consider all causes irrespective of their location on the death certificate
Transparent reporting of methods	When reporting the application of multiple cause methods, include the cause list used and information on how ill-defined causes and duplicate mentions were handled. If weighting strategies are applied, include the type of causes considered for weighting (e.g. associated or contributing or whether from Part I or Part II of the death certificate)

## Discussion

This study provides comprehensive population-level estimates of mortality in Australia integrating multiple causes of death. Substantial differences in rates based on UC and ‘any-mention’ were observed, but the differences reduced in magnitude with MC-weighting. IHD, dementia and cerebrovascular diseases ranked as leading causes by all methods; diabetes and COPD consistently ranked among the eight leading causes. Despite the similarities in some leading causes, with the integration of MC several preventable diseases emerged as leading causes. These included renal failure and atrial fibrillation, which ranked higher with weighting compared with using UC alone, and hypertension and heart failure, which emerged as leading causes of death under any-mention and MC-weighting. Our results confirm that monitoring population health considering only the UC in mortality indicators is insufficient for capturing the extensive contribution of major preventable causes of death. Indicators incorporating MC data offer an additional perspective for population health monitoring and policy development, enhancing available evidence on factors contributing to death.

Our application of the SRMU confirmed that health conditions that can act as risk factors (e.g. hypertension, atrial fibrillation, diabetes, osteoarthritis and mood disorders) and complications of diseases (e.g. renal failure, pneumonia and medically related adverse reactions) are less likely to feature as the UC but are involved in substantial numbers of deaths. Our results also support international estimates showing high SRMU for deaths attributable to these conditions, e.g. diabetes and renal failure,^9^^,^^10^^,^^12^^,^^15^^,^^25^^,^^26^ infectious diseases,^23^ mental health conditions,^27^^,^^28^ COPD^29^ and hypertension.^30^ Like other studies,^31–33^ we also found higher SRMUs for cancers with long relative survival and vice versa. The sizeable decrease in rates for some cancers (lung cancer, unknown primary site cancer and liver cancer) by MC-weighting compared with UC, as demonstrated in this study, supports the notion that MC analysis is not relevant to all causes of death,^33^ particularly those with low (close to 1.0) SRMU. Further studies could be undertaken to develop standards or thresholds to indicate causes suited to UC versus MC-weighting analysis. Further, the any-mention analyses showed that common conditions such as IHD, dementia, cerebrovascular disease, COPD, renal failure and diabetes contribute substantially more to death than recognized by the UC alone, underscoring the value of multiple-cause analysis in understanding their true impact on mortality. The SRMU and weighted MC analysis also highlighted the fact that cancers and external causes are almost always certified as underlying causes of death.

Few studies to date have applied MC-weighting methods^6^^,^^7^^,^^24^^,^^34^ and direct comparisons of our findings are hindered by methodological differences. Nevertheless, our findings on the direction of rate changes for major cause groups (increases or decreases) were consistent with those from a previous study that had applied the same weighting approach to data from France.^7^ However, there were differences in the magnitude of some of the rate changes, particularly for mental, musculoskeletal and genitourinary conditions, for which our weighted MC rates increased by 139%, 75% and 55% over the UC rates, as compared with rate increases of 34%, 11% and 24% from the French analysis.^7^ More broadly, differences may arise from variation across studies in the weighting strategy applied, the cause list used, the treatment of ill-defined and duplicate causes, and differences in coding or certification practices between countries. As ill-defined causes provide no additional insight^22^^,^^35^^,^^36^ they were excluded from our analysis. Also, duplicate mappings of individual ICD-10 codes to the cause list were excluded to avoid overestimating their contribution to mortality. Adoption of common standards and practices for these aspects of MC analysis, as outlined in Table 4, would facilitate comparisons across studies. Above all, complete and transparent reporting of analytical methods is necessary, to assist interpretation of indicators incorporating MC.

The increasing proportion of deaths involving more than four contributing causes is likely a reflection of increasing multimorbidity in an ageing Australian population. As noted elsewhere, the slight drop in the average number causes for older ages (≥95) could reflect less thorough death certification at very old ages or the healthy survivor effect.^16^^,^^31^^,^^37^ The notable increase in average number of causes in coroner-certified deaths, particularly since 2013, coincides with continued increases in suicides, falls and accidental poisoning deaths,^38^ as well as significant administrative changes that led to improved availability of data^38^ and likely reflects the joint impact of these factors.

Weighting methods conceptualize death as the outcome of a combination of conditions, with the size of weight reflecting the relative contribution or causal responsibility of each cause.^6^ To inform prevention strategies that aim to improve health at the population level, it is relevant to consider only conditions or exposures antecedent to or concurrent with the UC, rather than terminal conditions initiated by the UC.^6^ To uphold this, we restricted MC-weighting to contributing causes listed in Part II of the death certificate, ignoring health conditions that occur as consequences or complications of the UC (from Part I), thus ascribing all responsibility to the UC and comorbid chronic diseases, risk factors and relevant previous illness.^6^^,^^7^

In addition to evaluation of a broad spectrum of causes and inclusion of vast amounts of underused pathological information, a major advantage of MC-weighting is the preservation of the statistical or counting unit (deaths) which avoids double counting of deaths, enabling re-evaluation of mortality rates incorporating the contribution of each cause involved in each death.^6^^,^^7^ Further, MC-weighting demonstrates a more interpretable approach than application of the often-used any-mention method, which although useful for identifying the extent of the role of causes, provides highly misleading mortality indicators. All known weighting methods are arbitrary, (including ascribing 100% to the UC),^6^ as it is not possible to estimate the contribution of each cause based on cause of death data alone. Nonetheless, the MC-weighting strategy in this study maintains both the significance of the UC and overall involvement of CC, and our sensitivity analysis showed no differences in rates to our chosen method. Of note, the restriction to causes listed in Part II of the death certificate for MC-weighting purposes could overlook instances for certain conditions that are subject to ICD coding modification rules (e.g. diabetes, hypertension),^39^ where they could be listed in Part 1 but not be nominated as the underlying cause. Further research is needed to carefully disentangle such intricacies in death certification and coding practices, to better inform MC-weighting methods for quantification of mortality from these conditions in future. The development of a standard set of non-arbitrary weights for population-level analysis poses a challenging task, but disease-risk models could be used to inform the value of weights for targeted MC analyses.^6^^,^^7^

As demonstrated, analyses using MC-weighting methods, together with sensitivity analyses, can provide interpretable estimates for monitoring mortality burden which incorporate the contribution of each disease listed on individual death certificates.

## Conclusion

The results from this study confirm that monitoring population health using mortality indicators considering only the UC is insufficient for capturing the extensive contribution of major preventable causes of death. Cause-related mortality patterns based on MC data offer an additional perspective for informing public health prevention strategies and monitoring mortality burden. This study demonstrates the feasibility of the application of MC methods, including a more complex method of MC-weighting that could be applied more broadly to monitor the contribution of conditions amenable to effective population-based prevention strategies for reducing mortality.

## Ethics approval

Human Ethics Protocol was approved by the ANU Science and Medical Delegated Ethical Review Committee (Protocol number 2019/022).

## Supplementary Material

dyac167_Supplementary_Data

## Data Availability

This study used death records from each of the State and Territory Registries of Births, Deaths and Marriages and from State and Chief Coroners through the National Coronial Information System. The underlying data were provided the Australian Coordinating Registry (ACR) for Cause of Death Unit Record File. Restrictions apply to the availability of these data, which were used under licence for the current study, and so are not publicly available. Data are however available to approved users meeting the eligibility requirements through an application process administered by the ACR.
