# Supplementary material for: Quantifying cause-related mortality in Australia, incorporating multiple causes: observed patterns, trends and practical considerations
Source: Int J Epidemiol. 2022 Aug 19;52(1):284–94. doi: 10.1093/ije/dyac167 (PMC9908048; doi:10.1093/ije/dyac167)
Supplement: dyac167_Supplementary_Data [file dyac167_supplementary_data.zip › dyac167_Supplementary_Data/Sup File1.pdf]

Supplementary File 1 LITERATURE REVIEW OF METHODS AND PRACTICES USED TO ANALYSE MULTIPLE CAUES OF DEATH DATA

for Quantifying cause-related mortality in Australia incorporating multiple causes: observed patterns, trends, and practical considerations

For the most recent version of the review, please see:

https://medrxiv.org/cgi/content/short/2022.08.01.22278086v1

Aim: To identify and appraise methods and practices used to analyse multiple causes of death data.

Description of Methods: We searched four databases, including the Web of Science, Medline, Pubmed and Scopus, from inception to August 2020, with no language restriction. Eligible studies included those which utilised multiple causes of death (MCoD) to quantify mortality.

Outcome: Method used in the analyses of multiple cause of death data.

Inclusion criteria:

Study designs: Published, peer-reviewed original research articles reporting observational studies (cross-sectional, cohort, case-control)

Data source: Multiple causes of death data from death certificates or vital registrations

Outcome: Multiple cause of death indicator/method/measure used

Timing: Database inception through to August 2020

Setting: Any country

Language: Articles reported in all languages

Exclusion criteria:

Study designs: Conference abstracts, letters, editorials, correspondence, opinion pieces, government reports, position statements, qualitative research, intervention studies (e.g. randomised controlled trials), protocols.

Data source: Did not use multiple causes of death from death certificates or vital registrations (e.g. case studies, forensic reports, verbal autopsy)

Outcome: Studies that used multiple causes of death data but did not reported a multiple cause of death indicator/method/measure.

Timing: No exclusion criteria

Setting: No exclusion criteria

Language: No exclusion criteria

Search strategy:

Search terms

1. Multiple cause(s) of death
2. Multiple cause(s) of mortality
3. Contributory cause(s) of death
4. Contributory cause(s) of mortality
5. Underlying and contributory cause(s) of death
6. Underlying and contributory cause(s) of mortality
7. Associated cause(s) of death
8. Associated cause(s) of mortality

Databases: Web of Science, Medline, Pubmed and Scopus, from inception to 28th August 2020.

Screening: Results from the search were imported into EndNote version 20. After removing duplicates, the titles and abstracts of the articles were screened. Articles were selected for full text review if they indicated use of multiple cause of death data. Articles that met all of the inclusion criteria at full text screening were included in the review. Of the records identified through database searching (n = 8002), 3998 were duplicates, and another 3512 were excluded based on eligibility criteria; 10 articles were identified through hand search and audit, leaving 502 articles potentially eligible for full text review. After excluding 36 irretrievable articles and 57 based on eligibility criteria, 409 articles were included in the full text review.

Results: Of the 409 articles reviewed most (75%) were published since 2001 and were largely from countries with robust vital registration systems and mortality collections (the United States, Europe, United Kingdom, and Brazil). Research objectives included descriptive analysis of mentions, investigation of underlying cause epidemiology, investigation of immediate cause epidemiology, investigation of external cause of death, investigation of mortality risk factors, exploratory analyses of causes listed (e.g. uses network analysis), investigation of process of coding of causes of death and quantification of disease burden using multiple cause weighting approaches. Papers analysing multiple causes were highly skewed towards the application of multiple cause summary measures such as rates based on any mention with/without comparisons to rates by underlying cause (85%, n=346)(1–346) and those assessing pairwise contribution of causes to mortality (54%, n=222),(1–163, 347–405) with very few quantifying mortality based on groupings or clusters of diseases (2.0%, n=8)(

161–163, 344–346, 408, 409) and fewer using multiple causes weighting methods (1.0%, n=4).(342, 343, 406, 407) There were 16 articles that used population-based multiple cause of death data from Australia,(20, 37, 46, 74, 92, 108, 111, 353, 378, 418, 456, 497, 531, 582, 616, 641); all focussed on a specific cause of death (e.g. diabetes, renal disease, suicide).

Interpretation: Our systematic review confirms rapidly increasing international interest in the use of multiple causes of death data in mortality research, and provides the most comprehensive overview of methods and practices to date. Methods based on any mention offer an additional perspective on specific causes under consideration and are commonly applied; the main limitation is that deaths are being counted multiple times. Multiple cause weighting methods allow consideration of all relevant causes for the research objective and quantification of mortality without double counting of deaths, but the weights applied in previous studies remain arbitrary.

References

1. Adekoya, N. and Majumder, R. Fatal traumatic brain injury, West Virginia, 1989-1998. Public Health Rep 2004; 119:486-92. 10.1016/j.phr.2004.07.006
2. Adih, W. K., Selik, R. M. and Hu, X. Trends in Diseases Reported on US Death Certificates That Mentioned HIV Infection, 1996-2006. J Int Assoc Physicians AIDS Care (Chic) 2011; 10:5-11. 10.1177/1545109710384505
3. Alicandro, G., Frova, L., Di Fraia, G. and Colombo, C. Cystic fibrosis mortality trend in Italy from 1970 to 2011. J Cyst Fibros 2015; 14:267-74. 10.1016/j.jcf.2014.07.010
4. Amaral, T. L. M., Amaral, C. A., Miranda Filho, A. L. and Monteiro, G. T. R. Trends and multiple causes of death due to chronic renal failure in a municipality in the Brazilian Amazon. Cien Saude Colet 2018; 23:3821-3828. 10.1590/1413-812320182311.29902016
5. Andreasyan, K. and Hoy, W. E. Renal-related deaths in Indigenous people in Queensland, Australia. Nephrology 2007; 12:514-519. 10.1111/j.1440-1797.2007.00834.x
6. Arif, N., Yousfi, S. and Vinnard, C. Deaths from necrotizing fasciitis in the United States, 2003-2013. Epidemiol Infect 2016; 144:1338-44. 10.1017/s0950268815002745
7. Ascoli, V., Minelli, G., Kanieff, M., Crialesi, R., Frova, L. and Conti, S. Cause-specific mortality in classic Kaposi's sarcoma: a population-based study in Italy (1995-2002). Br J Cancer 2009; 101:1085-90. 10.1038/sj.bjc.6605265
8. Ascoli, V., Minelli, G., Kanieff, M., Frova, L. and Conti, S. Merkel cell carcinoma: a population-based study on mortality and the association with other cancers. Cancer Causes Control 2011; 22:1521-7. 10.1007/s10552-011-9826-4
9. Avouac, J., Amrouche, F., Meune, C., Rey, G., Kahan, A. and Allanore, Y. Mortality profile of patients with rheumatoid arthritis in France and its change in 10 years. Semin Arthritis Rheum 2017; 46:537-543. 10.1016/j.semarthrit.2016.10.007
10. Barragan, N. C., Sorvillo, F. and Kuo, T. Cryptococcosis-related deaths and associated medical conditions in the United States, 2000-2010. Mycoses 2014; 57:741-6. 10.1111/myc.12238
11. Bate, J., Ladhani, S., Sharland, M., Chisholm, J., Lamagni, T., Ramsay, M., Johnson, A. and Pebody, R. Infection-related mortality in children with malignancy in England and Wales, 2003-2005. Pediatr Blood Cancer 2009; 53:371-4. 10.1002/pbc.22057
12. Baum, H. M. and Manton, K. G. National trends in stroke-related mortality: a comparison of multiple cause mortality data with survey and other health data. Gerontologist 1987; 27:293-300. 10.1093/geront/27.3.293

13. Bennion, J. R., Sorvillo, F., Wise, M. E., Krishna, S. and Mascola, L. Decreasing listeriosis mortality in the United States, 1990-2005. Clinical Infectious Diseases 2008; 47:867-874. 10.1086/591131
14. Bi, P., Parton, K. A. and Whitby, M. Co-existing conditions for deaths from infectious and parasitic diseases in Australia. Int J Infect Dis 2004; 8:121-5. 10.1016/j.ijid.2003.05.002
15. Boyle, C. A., Decoufle, P. and Holmgreen, P. Contribution of developmental-disabilities to childhood mortality in the United-States - A multiple casue-of-death analysis. Am J Epidemiol 1993; 138:665-665.
16. Buschner, A. and Grunwald-Muhlberger, A. Influence of methodological changes on unicausal cause-of-death statistics and potentials of a multicausal data basis. Bundesgesundheitsblatt-Gesundheitsforschung-Gesundheitsschutz 2019; 62:1476-1484. 10.1007/s00103-019-03048-z
17. Cardoso, B. B. and Kale, P. L. Coding pulmonary sepsis and mortality statistics in Rio de Janeiro, RJ. Rev Bras Epidemiol 2016; 19:609-620. 10.1590/1980-5497201600030011
18. Chamblee, R. F. and Evans, M. C. New dimensions in cause of death statistics. Am J Public Health 1982; 72:1265-70. 10.2105/ajph.72.11.1265
19. Cheng, Y., Han, X., Luo, Y. and Xu, W. Deaths of obstructive lung disease in the Yangpu district of Shanghai from 2003 through 2011: a multiple cause analysis. Chin Med J (Engl) 2014; 127:1619-25.
20. Chiavegatto Filho, A. D. P., Laurenti, R., Gotlieb, S. L. D. and Jorge, M. H. P. D. M. Malnutrition as an underlying or associated cause of death: Analysis of the quality of information on women of reproductive age. Revista Brasileira de Epidemiologia 2007; 10:30-38. 10.1590/s1415-790x2007000100004
21. Chorba, T. L., Holman, R. C., Clarke, M. J. and Evatt, B. L. Effects of HIV infection on age and cause of death for persons with hemophilia A in the United States. Am J Hematol 2001; 66:229-40. 10.1002/ajh.1050
22. Coren, S. and Hewitt, P. L. Is anorexia nervosa associated with elevated rates of suicide? Am J Public Health 1998; 88:1206-1207. 10.2105/AJPH.88.8.1206
23. Costi, L. R., Iwamoto, H. M., Neves, D. C. D. and Caldas, C. A. M. Mortality from systemic erythematosus lupus in Brazil: evaluation of causes according to the government health database. Rev Bras Reumatol Engl Ed 2017; 57:574-582. 10.1016/j.rbre.2017.09.001
24. Craig Hooper, W., Holman, R. C., Clarke, M. J. and Chorba, T. L. Trends in non-Hodgkin lymphoma (NHL) and HIV-associated NHL deaths in the United States. Am J Hematol 2001; 66:159-166. 10.1002/1096-8652(200103)66:3<159::AID-AJH1039>3.0.CO;2-2
25. Crews, D. E., Stamler, J. and Dyer, A. Conditions other than underlying cause of death listed on death certificates provide additional useful information for epidemiologic research. Epidemiology 1991; 2:271-5. 10.1097/00001648-199107000-00006
26. Croker, C., Reporter, R., Redelings, M. and Mascola, L. Strongyloidiasis-related deaths in the United States, 1991-2006. American Journal of Tropical Medicine and Hygiene 2010; 83:422-426. 10.4269/ajtmh.2010.09-0750
27. Cummings, P. L., Sorvillo, F. and Kuo, T. Salmonellosis-related mortality in the United States, 1990-2006. Foodborne Pathog Dis 2010; 7:1393-9. 10.1089/fpd.2010.0588
28. Cummings, P. L., Kuo, T., Javanbakht, M. and Sorvillo, F. Trends, productivity losses, and associated medical conditions among toxoplasmosis deaths in the United States, 2000-2010. Am J Trop Med Hyg 2014; 91:959-64. 10.4269/ajtmh.14-0287
29. Cutter, J. L. Diabetes mortality in Singapore, 1991--a preliminary study. Singapore Med J 1998; 39:311-8.
30. Da Silva, L. R., Araújo, E. T. H., Carvalho, M. L., Almeida, C. A. P. L., Da Silva Oliveira, A. D., De Carvalho, P. M. G., Rodrigues, T. S. and Campelo, V. Epidemiological situation of acquired immunodeficiency syndrome (Aids)-related mortality in a municipality in northeastern brazil. a retrospective cross-sectional study. Sao Paulo Medical Journal 2018; 136:37-43. 10.1590/1516-3180.2017.0130100917

31. Désesquelles, A., Salvatore, M. A., Frova, L., Pace, M., Pappagallo, M., Meslé, F. and Egidi, V. Revisiting the mortality of France and Italy with the multiple-cause-of-death approach. Demogr Res 2010; 23:771-805. 10.4054/DemRes.2010.23.28
32. Désesquelles, A. F., Salvatore, M. A., Pappagallo, M., Frova, L., Pace, M., Meslé, F. and Egidi, V. Analysing Multiple Causes of Death: Which Methods For Which Data? An Application to the Cancer-Related Mortality in France and Italy. European Journal of Population-Revue Europeenne De Demographie 2012; 28:467-498. 10.1007/s10680-012-9272-3
33. Désesquelles, A., Demuru, E., Salvatore, M. A., Pappagallo, M., Frova, L., Mesle, F. and Egidi, V. Mortality from Alzheimer's disease, Parkinson's disease, and dementias in France and Italy: a comparison using the multiple cause-of-death approach. J Aging Health 2014; 26:283-315. 10.1177/0898264313514443
34. Désesquelles, A., Demuru, E., Pappagallo, M., Frova, L., Meslé, F. and Egidi, V. After the epidemiologic transition: a reassessment of mortality from infectious diseases among over-65s in France and Italy. Int J Public Health 2015; 60:961-7. 10.1007/s00038-015-0704-9
35. Dong, C., Yoon, Y. H., Chen, C. M. and Hsiao-Ye, Y. Heavy alcohol use and premature death from hepatocellular carcinoma in the United States, 1999-2006. J Stud Alcohol Drugs 2011; 72:892-902. 10.15288/jsad.2011.72.892
36. Duncan, M. E. and Goldacre, M. J. Mortality trends for benign prostatic hyperplasia and prostate cancer in English populations 1979-2006. BJU Int 2011; 107:40-5. 10.1111/j.1464-410X.2010.09487.x
37. Duncan, M. Trends in mortality for agranulocytosis in English populations, 1979-2010 and associations with cancer. British Journal of Haematology 2013; 161:738-751.
38. Duncan, M. E., Pitcher, A. and Goldacre, M. J. Atrial fibrillation as a cause of death increased steeply in England between 1995 and 2010. Europace 2014; 16:797-802. 10.1093/europace/eut388
39. Durkin, A., Connolly, S. and O'Reilly, D. Quantifying alcohol-related mortality: should alcohol-related contributory causes of death be included? Alcohol 2010; 45:374-8. 10.1093/alcalc/agq025
40. Ecclestone, T. C., Yeates, D. G. and Goldacre, M. J. Fall in population-based mortality from coronary heart disease negated in people with diabetes mellitus: data from England. Diabet Med 2015; 32:1329-34. 10.1111/dme.12770
41. Evans, J. M., Barnett, K. N., McMurdo, M. E. and Morris, A. D. Reporting of diabetes on death certificates of 1872 people with type 2 diabetes in Tayside, Scotland. Eur J Public Health 2008; 18:201-3. 10.1093/eurpub/ckm113
42. Fearnley, E., Li, S. Q. and Guthridge, S. Trends in chronic disease mortality in the Northern Territory Aboriginal population, 1997-2004: using underlying and multiple causes of death. Aust N Z J Public Health 2009; 33:551-5. 10.1111/j.1753-6405.2009.00452.x
43. Fedeli, U., Avossa, F., Goldoni, C. A., Caranci, N., Zambon, F. and Saugo, M. Education level and chronic liver disease by aetiology: A proportional mortality study. Dig Liver Dis 2015; 47:1082-5. 10.1016/j.dld.2015.07.154
44. Fedeli, U. and Schievano, E. Increase in Parkinson's disease-related mortality among males in Northern Italy. Parkinsonism Relat Disord 2017; 40:47-50. 10.1016/j.parkreldis.2017.04.008
45. Fedeli, U., Grande, E., Grippo, F. and Frova, L. Mortality associated with hepatitis C and hepatitis B virus infection: A nationwide study on multiple causes of death data. World J Gastroenterol 2017; 23:1866-1871. 10.3748/wjg.v23.i10.1866
46. Fingerhut, L. A. and Cox, C. S. Poisoning mortality, 1985-1995. Public Health Rep 1998; 113:218-33.
47. Ford, M. M., Desai, P. S., Maduro, G. and Laraque, F. Neighborhood Inequalities in Hepatitis C Mortality: Spatial and Temporal Patterns and Associated Factors. J Urban Health 2017; 94:746-755. 10.1007/s11524-017-0174-x

48. Franco, L. J., Mameri, C., Pagliaro, H., Iochida, L. C. and Goldenberg, P. Diabetes as underlying or associated cause of death in the State of S. Paulo, 1992. Rev Saude Publica 1998; 32:237-245. 10.1590/s0034-89101998000300006
49. García Benavides, F., Godoy, C., Pérez, S. and Bolumar, F. [Multiple codification of the causes of death: from dying "of" to dying "from"]. Gac Sanit 1992; 6:53-7. 10.1016/s0213-9111(92)71092-9
50. Garcia-Ptacek, S., Kareholt, I., Cermakova, P., Rizzuto, D., Religa, D. and Eriksdotter, M. Causes of Death According to Death Certificates in Individuals with Dementia: A Cohort from the Swedish Dementia Registry. J Am Geriatr Soc 2016; 64:e137-e142. 10.1111/jgs.14421
51. Goldacre, M. J., Mant, D., Duncan, M. and Griffith, M. Mortality from heart failure in an English population, 1979–2003: study of death certification. Journal of Epidemiology and Community Health 2005; . 10.1136/jech.2004.028951
52. Goldacre, M. J., Duncan, M. E., Griffith, M. and Davidson, M. Trends in mortality from appendicitis and from gallstone disease in English populations, 1979-2006: study of multiple-cause coding of deaths. Postgrad Med J 2011; 87:245-50. 10.1136/pgmj.2010.104471
53. Goldacre, M. J. and Duncan, M. E. Death rates for acquired hypothyroidism and thyrotoxicosis in English populations (1979-2010): comparison of underlying cause and all certified causes. QJM 2013; 106:229-35. 10.1093/qjmed/hct011
54. Gorina, Y. and Lentzner, H. Multiple causes of death in old age. Aging Trends 2008;1-9.
55. Grande, E., Zucchetto, A., Suligoi, B., Grippo, F., Pappagallo, M., Virdone, S., Camoni, L., Taborelli, M., Regine, V., Serraino, D. and Frova, L. Multiple cause-of-death data among people with AIDS in Italy: a nationwide cross-sectional study. Popul Health Metr 2017; 15:19. 10.1186/s12963-017-0135-3
56. Griffiths, E. C., Pedersen, A. B., Fenton, A. and Petchey, O. L. Reported co-infection deaths are more common in early adulthood and among similar infections. BMC Infect Dis 2015; 15. 10.1186/s12879-015-1118-2
57. Grippo, F., Pappagallo, M., Burgio, A. and Crialesi, R. Drug induced mortality: A multiple cause approach on italian causes of death register. Epidemiology Biostatistics and Public Health 2015; 12:e-1. 10.2427/11177
58. Gu, K., Cowie, C. C. and Harris, M. I. Mortality in adults with and without diabetes in a national cohort of the U.S. population, 1971-1993. Diabetes Care 1998; 21:1138-45. 10.2337/diacare.21.7.1138
59. Guralnick, L. Some problems in the use of multiple causes of death. J Chronic Dis 1966; 19:979-90. 10.1016/0021-9681(66)90031-2
60. Harding, A. H. and Darnton, A. J. Asbestosis and mesothelioma among British asbestos workers (1971-2005). Am J Ind Med 2010; 53:1070-80. 10.1002/ajim.20844
61. Harzke, A. J., Baillargeon, J., Paar, D. P., Pulvino, J. and Murray, O. J. Chronic liver disease mortality among male prison inmates in Texas, 1989-2003. The American journal of gastroenterology 2009; . 10.1038/ajg.2009.106
62. Horlander, K. R., Mannino, D. M. and Leeper, K. V. Pulmonary embolism mortality in the United States, 1979-1998 - An analysis using multiple-cause mortality data. Arch Intern Med 2003; 163:1711-1717. 10.1001/archinte.163.14.1711
63. Janssen, T. A. Importance of Tabulating Multiple Causes of Death. Am J Public Health Nations Health 1940; 30:871-9. 10.2105/ajph.30.8.871
64. Johnson, N. E. and Christenson, B. A. Socio-demographic correlates of multiple causes of death: Real or artifactual? Population Research and Policy Review 1998; 17:261-274. Doi 10.1023/A:1005985730638
65. Juel, K. and Helweg-Larsen, K. Drug-related mortality in Denmark 1970-93. Scand J Public Health 1999; 27:48-53.

66. Jung, R. S., Bennion, J. R., Sorvillo, F. and Bellomy, A. Trends in tuberculosis mortality in the United States, 1990-2006: a population-based case-control study. Public Health Rep 2010; 125:389-97. 10.1177/003335491012500307
67. Kenneson, A., Kolor, K., Yang, Q., Olney, R. S., Rasmussen, S. A. and Friedman, J. M. Trends and racial disparities in muscular dystrophy deaths in the United States, 1983-1998: an analysis of multiple cause mortality data. Am J Med Genet A 2006; 140:2289-97. 10.1002/ajmg.a.31437
68. Kiadaliri, A. A., Turkiewicz, A. and Englund, M. Mortality from Musculoskeletal Disorders Including Rheumatoid Arthritis in Southern Sweden: A Multiple-cause-of-death Analysis, 1998-2014. J Rheumatol 2017; 44:571-579. 10.3899/jrheum.161219
69. Kiadaliri, A. A., Rosengren, B. E. and Englund, M. Fracture-related mortality in southern Sweden: A multiple cause of death analysis, 1998-2014. Injury 2018; 49:236-242. 10.1016/j.injury.2017.12.005
70. Kiadaliri, A. A., Rosengren, B. E. and Englund, M. Fall-related mortality in southern Sweden: a multiple cause of death analysis, 1998-2014. Inj Prev 2019; 25:129-135. 10.1136/injuryprev-2017-042425
71. Kishamawe, C., Rumisha, S. F., Mremi, I. R., Bwana, V. M., Chiduo, M. G., Massawe, I. S. and Mboera, L. E. G. Trends, patterns and causes of respiratory disease mortality among inpatients in Tanzania, 2006-2015. Trop Med Int Health 2019; 24:91-100. 10.1111/tmi.13165
72. Kugeler, K. J., Griffith, K. S., Gould, L. H., Kochanek, K., Delorey, M. J., Biggerstaff, B. J. and Mead, P. S. A review of death certificates listing Lyme disease as a cause of death in the United States. Clin Infect Dis 2011; 52:364-7. 10.1093/cid/ciq157
73. Landes, S. D., Stevens, J. D. and Turk, M. A. Obscuring effect of coding developmental disability as the underlying cause of death on mortality trends for adults with developmental disability: a cross-sectional study using US Mortality Data from 2012 to 2016. BMJ Open 2019; 9:e026614. 10.1136/bmjopen-2018-026614
74. Lee, D. S., Gona, P., Albano, I., Larson, M. G., Benjamin, E. J., Levy, D., Kannel, W. B. and Vasan, R. S. A Systematic Assessment of Causes of Death After Heart Failure Onset in the Community Impact of Age at Death, Time Period, and Left Ventricular Systolic Dysfunction. Circulation-Heart Failure 2011; 4:36-U85. 10.1161/circheartfailure.110.957480
75. Leone, M., Chandra, V. and Schoenberg, B. S. Motor neuron disease in the United States, 1971 and 1973–1978: Patterns of mortality and ssociated conditions at the time of death. Neurology 1987; 37:1339-1343. 10.1212/wnl.37.8.1339
76. Lethbridge, L., Johnston, G. M. and Turnbull, G. Co-morbidities of persons dying of Parkinson's disease. Progress in Palliative Care 2013; 21:140-145. 10.1179/1743291X12Y.0000000037
77. Li, S. Q., Cunningham, J. and Cass, A. Renal-related deaths in Australia 1997-1999. Intern Med J 2004; 34:259-65. 10.1111/j.1444-0903.2004.00565.x
78. Lin, J. C. and Nichol, K. L. Excess mortality due to pneumonia or influenza during influenza seasons among persons with acquired immunodeficiency syndrome. Arch Intern Med 2001; 161:441-6. 10.1001/archinte.161.3.441
79. Lott, J. P. and Gross, C. P. Mortality from nonneoplastic skin disease in the United States. J Am Acad Dermatol 2014; 70:47-54 e1. 10.1016/j.jaad.2013.09.039
80. Ly, K. N., Xing, J., Klevens, R. M., Jiles, R. B., Ward, J. W. and Holmberg, S. D. The increasing burden of mortality from viral hepatitis in the United States between 1999 and 2007. Ann Intern Med 2012; 156:271-8. 10.7326/0003-4819-156-4-201202210-00004
81. Ly, K. N. and Klevens, R. M. Trends in disease and complications of hepatitis A virus infection in the United States, 1999-2011: a new concern for adults. J Infect Dis 2015; 212:176-82. 10.1093/infdis/jiu834
82. Mackenbach, J. P., Kunst, A. E., Lautenbach, H., Oei, Y. B. and Bijlsma, F. Competing causes of death: a death certificate study. J Clin Epidemiol 1997; 50:1069-77. 10.1016/s0895-4356(97)00165-0

83. Mackenbach, J. P., Kunst, A. E., Lautenbach, H., Bijlsma, F. and Oei, Y. B. Competing causes of death: an analysis using multiple-cause-of-death data from The Netherlands. Am J Epidemiol 1995; 141:466-75. 10.1093/oxfordjournals.aje.a117449
84. Mannino, D. M., Ford, E., Giovino, G. A. and Thun, M. Lung cancer deaths in the United States from 1979 to 1992: an analysis using multiple-cause mortality data. Int J Epidemiol 1998; 27:159-66. 10.1093/ije/27.2.159
85. Masocco, M., Kodra, Y., Vichi, M., Conti, S., Kanieff, M., Pace, M., Frova, L. and Taruscio, D. Mortality associated with neurofibromatosis type 1: a study based on Italian death certificates (1995-2006). Orphanet J Rare Dis 2011; 6:11. 10.1186/1750-1172-6-11
86. Maynard, C., Lowy, E., McDonell, M. and Fihn, S. D. Cause of death in Washington state veterans hospitalized with acute coronary syndromes in the veterans health administration. Popul Health Metr 2008; 6:3. 10.1186/1478-7954-6-3
87. McEwen, L. N., Kim, C., Haan, M., Ghosh, D., Lantz, P. M., Mangione, C. M., Safford, M. M., Marrero, D., Thompson, T. J. and Herman, W. H. Diabetes reporting as a cause of death: results from the Translating Research Into Action for Diabetes (TRIAD) study. Diabetes Care 2006; 29:247-53. 10.2337/diacare.29.02.06.dc05-0998
88. McEwen, L. N., Karter, A. J., Curb, J. D., Marrero, D. G., Crosson, J. C. and Herman, W. H. Temporal trends in recording of diabetes on death certificates: results from Translating Research Into Action for Diabetes (TRIAD). Diabetes Care 2011; 34:1529-33. 10.2337/dc10-2312
89. McKenzie, K., Chen, L. and Walker, S. M. Correlates of undefined cause of injury coded mortality data in Australia. Health Inf Manag 2009; 38:8-14. 10.1177/183335830903800102
90. McNeil, M. M., Nash, S. L., Hajjeh, R. A., Phelan, M. A., Conn, L. A., Plikaytis, B. D. and Warnock, D. W. Trends in mortality due to invasive mycotic diseases in the United States, 1980-1997. Clin Infect Dis 2001; 33:641-7. 10.1086/322606
91. McPherson, D., Griffiths, C., Williams, M., Baker, A., Klodawski, E., Jacobson, B. and Donaldson, L. Sepsis-associated mortality in England: an analysis of multiple cause of death data from 2001 to 2010. BMJ Open 2013; 3. 10.1136/bmjopen-2013-002586
92. Murray, C. J., Dias, R. H., Kulkarni, S. C., Lozano, R., Stevens, G. A. and Ezzati, M. Improving the comparability of diabetes mortality statistics in the U.S. and Mexico. Diabetes Care 2008; 31:451-8. 10.2337/dc07-1370
93. Najafi, F., Dobson, A. J. and Jamrozik, K. Is mortality from heart failure increasing in Australia? An analysis of official data on mortality for 1997-2003. Bull World Health Organ 2006; 84:722-8. 10.2471/blt.06.031286
94. Nam, C. B., Hummer, R. A. and Rogers, R. G. Underlying and Multiple Causes of Death Related to Smoking. Population Research and Policy Review 1994; 13:305-325. Doi 10.1007/Bf01074340
95. Nashold, R. D. and Naor, E. M. Alcohol-related deaths in Wisconsin: the impact of alcohol on mortality. Am J Public Health 1981; 71:1237-41. 10.2105/ajph.71.11.1237
96. Nath, U., Thomson, R., Wood, R., Ben-Shlomo, Y., Lees, A., Rooney, C. and Burn, D. Population based mortality and quality of death certification in progressive supranuclear palsy (Steele-Richardson-Olszewski syndrome). J Neurol Neurosurg Psychiatry 2005; 76:498-502. 10.1136/jnnp.2004.039370
97. Nizamo, H., Meyrowitsch, D. W., Zacarias, E. and Konradsen, F. Mortality due to injuries in Maputo City, Mozambique. Int J Inj Contr Saf Promot 2006; 13:1-6. 10.1080/17457300500151705
98. Nizard, A. and Munozperez, F. Alcohol, tobacco, and mortality in France since 1950 - An estimate of the annual numbers of deaths. Population 1993; 48:571-607. 10.2307/1534096
99. Ochi, J. W., Melton, L. J., 3rd, Palumbo, P. J. and Chu, C. P. A population-based study of diabetes mortality. Diabetes Care 1985; 8:224-9. 10.2337/diacare.8.3.224
100. Olson, A. L., Swigris, J. J., Sprunger, D. B., Fischer, A., Fernandez-Perez, E. R., Solomon, J., Murphy, J., Cohen, M., Raghu, G. and Brown, K. K. Rheumatoid arthritis-interstitial lung disease-associated mortality. Am J Respir Crit Care Med 2011; 183:372-8. 10.1164/rccm.201004-0622OC

101. Owe, J. F., Daltveit, A. K. and Gilhus, N. E. Does myasthenia gravis provide protection against cancer? Acta Neurol Scand 2006; 113:33-36. 10.1111/j.1600-0404.2006.00612.x
102. Pacheco, A. G., Tuboi, S. H., Faulhaber, J. C., Harrison, L. H. and Schechter, M. Increase in non-AIDS related conditions as causes of death among HIV-infected individuals in the HAART era in Brazil. PLoS One 2008; 3:e1531. 10.1371/journal.pone.0001531
103. Paik, J. M., Henry, L., De Avila, L., Younossi, E., Racila, A. and Younossi, Z. M. Mortality Related to Nonalcoholic Fatty Liver Disease Is Increasing in the United States. Hepatol Commun 2019; 3:1459-1471. 10.1002/hep4.1419
104. Palladino, C., Climent, F. J., De Jose, M. I., De Ory, S. J., Bellon, J. M., Guillen, S., Gurbindo, M. D., Gonzalez-Tome, I., Mellado, M. J., Perez, J. M., Calvo, C., Ramos, J. T. and Munoz-Fernandez, M. A. Causes of Death in Pediatric Patients Vertically Infected by the Human Immunodeficiency Virus Type 1 in Madrid, Spain, From 1982 to Mid-2009. Pediatric Infectious Disease Journal 2011; 30:495-500. 10.1097/INF.0b013e318211399f
105. Park, C. B., Yokoyama, E. and Tokuyama, G. H. Medical Conditions at Death among the Caucasian and Japanese Elderly in Hawaii - Analysis of Multiple Causes of Death, 1976-78. Journal of Clinical Epidemiology 1991; 44:519-530. Doi 10.1016/0895-4356(91)90215-U
106. Park, J. Mortality from Alzheimer's disease in Canada: A multiple-cause-of-death analysis, 2004 to 2011. Health Rep 2016; 27:17-21.
107. Parks, S. E., Kegler, S. R., Annest, J. L. and Mercy, J. A. Characteristics of fatal abusive head trauma among children in the USA: 2003-2007: an application of the CDC operational case definition to national vital statistics data. Inj Prev 2012; 18:193-9. 10.1136/injuryprev-2011-040128
108. Paulozzi, L. J., Budnitz, D. S. and Xi, Y. Increasing deaths from opioid analgesics in the United States. Pharmacoepidemiol Drug Saf 2006; 15:618-27. 10.1002/pds.1276
109. Pechholdová, M. Multiple cause-of-death data in the Czech Republic: An exploratory analysis. Demografie 2014; 56:335-346.
110. Pechholdova, M. Sepsis-related mortality in the Czech Republic: multiple causes of death analysis. Epidemiologie, Mikrobiologie, Imunologie 2017; 66(2):73-79.
111. Peng, J. K., Higginson, I. J. and Gao, W. Place of death and factors associated with hospital death in patients who have died from liver disease in England: a national population-based study. Lancet Gastroenterol Hepatol 2019; 4:52-62. 10.1016/s2468-1253(18)30379-0
112. Pillutla, P., Sherry, K. D. and Foster, E. Mortality associated with adult congenital heart disease: Trends in the US population from 1979 to 2005. Am Heart J 2009; 158:874-879. 10.1016/j.ahj.2009.08.014
113. Pinheiro, F. A., Souza, D. C. and Sato, E. I. A Study of Multiple Causes of Death in Rheumatoid Arthritis. J Rheumatol 2015; 42:2221-8. 10.3899/jrheum.150166
114. Polednak, A. P. Surveillance of US deaths related to myelodysplastic syndromes, and the need for linkages with central cancer registries. J Registry Manag 2011; 38:183-9.
115. Polednak, A. P. Trend (1999-2009) in U.S. death rates from myelodysplastic syndromes: utility of multiple causes of death in surveillance. Cancer Epidemiol 2013; 37:569-74. 10.1016/j.canep.2013.05.004
116. Polednak, A. P. Trend in rates for deaths with mention of schizophrenia on death certificates of US residents, 1999-2010. Soc Psychiatry Psychiatr Epidemiol 2014; 49:1083-91. 10.1007/s00127-014-0846-8
117. Poston Jr, D. L. and Min, H. The multinomial regression modeling of the cause-of-death mortality of the oldest old in the U.S. Journal of Modern Applied Statistical Methods 2008; 7:597-606. 10.22237/jmasm/1225513380
118. Prescott, E., Lange, P. and Vestbo, J. Chronic mucus hypersecretion in COPD and death from pulmonary infection. Eur Respir J 1995; 8:1333-8. 10.1183/09031936.95.08081333
119. Rasmussen, S. A., Yang, Q. and Friedman, J. M. Mortality in neurofibromatosis 1: an analysis using U.S. death certificates. Am J Hum Genet 2001; 68:1110-8. 10.1086/320121

120. Riihimaki, M., Thomsen, H., Brandt, A., Sundquist, J. and Hemminki, K. Death causes in breast cancer patients. Ann Oncol 2012; 23:604-610. 10.1093/annonc/mdr160
121. Roaeid, R. B. and Kablan, A. A. Diabetes mortality and causes of death in Benghazi: a 5-year retrospective analysis of death certificates. East Mediterr Health J 2010; 16:65-9.
122. Rocha, M. S., de Oliveira, G. P., Aguiar, F. P., Saraceni, V. and Pinheiro, R. S. What are the causes of death of patients with tuberculosis: multiple causes of death in a cohort of cases and a research proposal of presumed causes. Cad Saude Publica 2015; 31:709-721. 10.1590/0102-311x00101214
123. Ruhm, C. J. Drug involvement in fatal overdoses. SSM Popul Health 2017; 3:219-226. 10.1016/j.ssmph.2017.01.009
124. Ruhm, C. J. Corrected US opioid-involved drug poisoning deaths and mortality rates, 1999-2015. Addiction 2018; 113:1339-1344. 10.1111/add.14144
125. Rushton, L. Use of multiple causes of death in the analysis of occupational cohorts--an example from the oil industry. Occup Environ Med 1994; 51:722-9. 10.1136/oem.51.11.722
126. Ruzicka, L. T., Choi, C. Y. and Sadkowsky, K. Medical disorders of suicides in Australia: analysis using a multiple-cause-of-death approach. Soc Sci Med 2005; 61:333-41. 10.1016/j.socscimed.2004.12.005
127. Salive, M. E., Satterfield, S., Ostfeld, A. M., Wallace, R. B. and Havlik, R. J. Disability and cognitive impairment are risk factors for pneumonia-related mortality in older adults. Public Health Rep 1993; 108:314-22.
128. Sampaio, V. D., Rodrigues, M. G. D., da Silva, L. C. F., de Castro, D. B., Balieiro, P. C. D., Cabrinha, A. A. and Costa, A. J. L. Social, demographic, health care and co-morbidity predictors of tuberculosis mortality in Amazonas, Brazil: a multiple cause of death approach (vol 15, e0218359, 2020). PLoS One 2020; 15. 10.1371/journal.pone.0229749
129. Santo, A. H., Pinheiro, C. E. and Jordani, M. S. [Aids as underlying and associated causes of death, State of S. Paulo, Brazil, 1998]. Rev Saude Publica 2000; 34:581-8. 10.1590/s0034-89102000000600004
130. Santo, A. H., Pinheiro, C. E. and Jordani, M. S. [Multiple-causes-of-death related to tuberculosis in the State of São Paulo, Brazil, 1998]. Rev Saude Publica 2003; 37:714-21. 10.1590/s0034-89102003000600005
131. Santo, A. H. Deaths attributed to multiple causes and involving tuberculosis in the state of Rio de Janeiro Brazil between 1999 and 2001. J Bras Pneumol 2006; 32:544-52. 10.1590/s1806-37132006000600012
132. Santo, A. H., Souza, J. M., Pinheiro, C. E., Souza, D. C. and Sato, E. I. Trends in dermatomyositis- and polymyositis-related mortality in the state of São Paulo, Brazil, 1985-2007: multiple cause-of-death analysis. BMC Public Health 2010; 10:597. 10.1186/1471-2458-10-597
133. Seuc, A. H., Fernandez, L., Mirabal, M., Rodriguez, A. and Rodriguez, C. A. Cuban Application of Two Methods for Analyzing Multiple Causes of Death. MEDICC Rev 2018; 20:30-35.
134. Sleeman, K. E., Ho, Y. K., Verne, J., Glickman, M., Silber, E., Gao, W. and Higginson, I. J. Place of death, and its relation with underlying cause of death, in Parkinson's disease, motor neurone disease, and multiple sclerosis: a population-based study. Palliat Med 2013; 27:840-6. 10.1177/0269216313490436
135. Souza, D. C., Santo, A. H. and Sato, E. I. Mortality profile related to systemic lupus erythematosus: a multiple cause-of-death analysis. J Rheumatol 2012; 39:496-503. 10.3899/jrheum.110241
136. Speizer, F. E., Trey, C. and Parker, P. The uses of multiple causes of death data to clarify changing patterns of cirrhosis mortality in Massachusetts. Am J Public Health 1977; 67:333-6. 10.2105/ajph.67.4.333
137. Stares, J. and Kosatsky, T. Hypothermia as a cause of death in British Columbia, 1998-2012: a descriptive assessment. CMAJ Open 2015; 3:E352-8. 10.9778/cmajo.20150013

138. Sterling, T. D., Rosenbaum, W. L. and Weinkam, J. J. Bias in the attribution of lung cancer as cause of death and its possible consequences for calculating smoking-related risks. Epidemiology 1992; 3:11-6. 10.1097/00001648-199201000-00004
139. Sweitzer, K. and Stallones, L. Significant contributing causes of cancer deaths among Hispanics in Colorado, USA, 1983-1992. Cad Saude Publica 1998; 14 Suppl 3:187-91. 10.1590/s0102-311x1998000700020
140. Swigris, J. J., Olson, A. L., Huie, T. J., Fernandez-Perez, E. R., Solomon, J., Sprunger, D. and Brown, K. K. Sarcoidosis-related mortality in the United States from 1988 to 2007. Am J Respir Crit Care Med 2011; 183:1524-30. 10.1164/rccm.201010-1679OC
141. Takamori, A., Takahashi, I., Kasagi, F., Suyama, A., Ozasa, K. and Yanagawa, T. Mortality Analysis of the Life Span Study (LSS) Cohort Taking into Account Multiple Causes of Death Indicated in Death Certificates. Radiat Res 2017; 187:20-31. 10.1667/rr14314.1
142. Tamashiro, H., Akagi, H., Arakaki, M., Futatsuka, M. and Roht, L. H. Causes of death in Minamata disease: analysis of death certificates. Int Arch Occup Environ Health 1984; 54:135-46. 10.1007/bf00378516
143. Thomas, G., Mancini, J., Jourde-Chiche, N., Sarlon, G., Amoura, Z., Harle, J. R., Jougla, E. and Chiche, L. Mortality associated with systemic lupus erythematosus in France assessed by multiple-cause-of-death analysis. Arthritis Rheumatol 2014; 66:2503-11. 10.1002/art.38731
144. Turner, C., Chandrakumar, D., Rowe, C., Santos, G. M., Riley, E. D. and Coffin, P. O. Cross-sectional cause of death comparisons for stimulant and opioid mortality in San Francisco, 2005-2015. Drug Alcohol Depend 2018; 185:305-312. 10.1016/j.drugalcdep.2017.12.030
145. Van Natta, P., Malin, H., Bertolucci, D. and Kaelber, C. The influence of alcohol abuse as a hidden contributor to mortality. Alcohol 1985; 2:535-9. 10.1016/0741-8329(85)90130-2
146. Vinnard, C., Longworth, S., Mezochow, A., Patrawalla, A., Kreiswirth, B. N. and Hamilton, K. Deaths Related to Nontuberculous Mycobacterial Infections in the United States, 1999-2014. Ann Am Thorac Soc 2016; 13:1951-1955. 10.1513/AnnalsATS.201606-474BC
147. Wattigney, W. A., Mensah, G. A. and Croft, J. B. Increased atrial fibrillation mortality: United States, 1980-1998. Am J Epidemiol 2002; 155:819-26. 10.1093/aje/155.9.819
148. White, M. C. Mortality associated with nosocomial infections: analysis of multiple cause-of-death data. J Clin Epidemiol 1993; 46:95-100.
149. White, M. C. and Portillo, C. J. Tuberculosis mortality associated with AIDS and drug or alcohol abuse: analysis of multiple cause-of-death data. Public Health 1996; 110:185-9. 10.1016/s0033-3506(96)80074-6
150. Whiteside, Y. O., Selik, R., An, Q., Huang, T., Karch, D., Hernandez, A. L. and Hall, H. I. Comparison of Rates of Death Having any Death-Certificate Mention of Heart, Kidney, or Liver Disease Among Persons Diagnosed with HIV Infection with those in the General US Population, 2009-2011. Open AIDS J 2015; 9:14-22. 10.2174/1874613601509010014
151. Wickramasekaran, R. N., Sorvillo, F. and Kuo, T. Legionnaires' disease and associated comorbid conditions as causes of death in the U.S., 2000-2010. Public Health Rep 2015; 130:222-9. 10.1177/003335491513000309
152. Wilkins, K., Parsons, G. F., Gentleman, J. F. and Forbes, W. F. Deaths due to dementia: An analysis of multiple-cause-of-death data. Chronic Dis Can 1999; 20:26-35.
153. Wong, O., Rockette, H. E., Redmond, C. K. and Heid, M. Evaluation of Multiple Causes of Death in Occupational Mortality Studies. Journal of Chronic Diseases 1978; 31:183-193. Doi 10.1016/0021-9681(78)90033-4
154. Yang, Q., McDonnell, S. M., Khoury, M. J., Cono, J. and Parrish, R. G. Hemochromatosis-associated mortality in the United States from 1979 to 1992: an analysis of Multiple-Cause Mortality Data. Ann Intern Med 1998; 129:946-53. 10.7326/0003-4819-129-11_part_2-199812011-00005
155. Yashin, A. I., Ukraintseva, S. V., Akushevich, I. V., Arbeev, K. G., Kulminski, A. and Akushevich, L. Trade-off between cancer and aging: what role do other diseases play? Evidence from

experimental and human population studies. Mech Ageing Dev 2009; 130:98-104. 10.1016/j.mad.2008.03.006
156. Yen, E. Y. and Singh, R. R. Lupus-An Unrecognized Leading Cause of Death in Young Females: A Population-Based Study Using Nationwide Death Certificates, 2000-2015. Arthritis & Rheumatology 2018; 70:1251-1255. 10.1002/art.40512
157. Yoon, Y. H., Stinson, F. S., Yi, H. Y. and Dufour, M. C. Accidental alcohol poisoning mortality in the United States, 1996-1998. Alcohol Res Health 2003; 27:110-8.
158. Zargar, A. H., Wani, A. I., Masoodi, S. R., Bashir, M. I., Laway, B. A., Gupta, V. K. and Wani, F. A. Causes of mortality in diabetes mellitus: data from a tertiary teaching hospital in India. Postgrad Med J 2009; 85:227-32. 10.1136/pgmj.2008.067975
159. Ziade, N., Jougla, E. and Coste, J. Population-level impact of osteoporotic fractures on mortality and trends over time: a nationwide analysis of vital statistics for France, 1968-2004. Am J Epidemiol 2010; 172:942-51. 10.1093/aje/kwq215
160. Zoppini, G., Fedeli, U., Gennaro, N., Saugo, M., Targher, G. and Bonora, E. Mortality from chronic liver diseases in diabetes. Am J Gastroenterol 2014; 109:1020-5. 10.1038/ajg.2014.132
161. Frova, L., Salvatore, M. A., Pappagallo, M. and Egidi, V. The multiple cause of death approach to analyse mortality patterns. Genus 2009; .
162. Stallard, E. Underlying and Multiple Case Mortality Advanced Ages: United States 1980-1998. North American Actuarial Journal 2002; 6:64-87. 10.1080/10920277.2002.11073999
163. Yoon, Y. H., Chen, C. M., Yi, H. Y. and Moss, H. B. Effect of comorbid alcohol and drug use disorders on premature death among unipolar and bipolar disorder decedents in the United States, 1999 to 2006. Compr Psychiatry 2011; 52:453-64. 10.1016/j.comppsych.2010.10.005
164. Bah, S. Using multiple-cause mortality data to resolve conflicting information on trends in maternal mortality in South Africa. S Afr Med J 2006; 96:308.
165. Baker, S. P., Brady, J. E., Shanahan, D. F. and Li, G. Aviation-related injury morbidity and mortality: data from U.S. health information systems. Aviat Space Environ Med 2009; 80:1001-5. 10.3357/asem.2575.2009
166. Balkau, B. and Papoz, L. Certification of cause of death in French diabetic patients. J Epidemiol Community Health 1992; 46:63-5. 10.1136/jech.46.1.63
167. Barreto, S. M., Passos, V. M., Almeida, S. K. and Assis, T. D. The increase of diabetes mortality burden among Brazilian adults. Rev Panam Salud Publica 2007; 22:239-45. 10.1590/s1020-49892007000900003
168. Burke, J. F., Lisabeth, L. D., Brown, D. L., Reeves, M. J. and Morgenstern, L. B. Determining stroke's rank as a cause of death using multicause mortality data. Stroke 2012; 43:2207-11. 10.1161/strokeaha.112.656967
169. Bustamante-Montes, L. P., Alvarez-Solorza, I., Valencia, A. D., Hernandez-Valero, M. A., Tlachino, G. T. and Huidobro, L. G. Applicability of the analysis by multiple cause of death by cervical cancer: The experience in Mexico. Cien Saude Colet 2011; 16:4815-4821. 10.1590/s1413-81232011001300030
170. Cascão, A. M., Costa, A. J. and Kale, P. L. [Quality of mortality information in a diabetes cohort--State of Rio de Janeiro, 2000 to 2003]. Rev Bras Epidemiol 2012; 15:134-42. 10.1590/s1415-790x2012000100012
171. Castle, I. J., Yi, H. Y., Hingson, R. W. and White, A. M. State variation in underreporting of alcohol involvement on death certificates: motor vehicle traffic crash fatalities as an example. J Stud Alcohol Drugs 2014; 75:299-312. 10.15288/jsad.2014.75.299
172. Coeli, C. M., Ferreira, L. G., Drbal Md Mde, M., Veras, R. P., Camargo, K. R., Jr. and Cascao, A. M. [Diabetes mellitus mortality among elderly as an underlying or secondary cause of death]. Rev Saude Publica 2002; 36:135-40. 10.1590/s0034-89102002000200003
173. Crews, D. E. Multiple causes of death and the epidemiological transition in American Samoa. Soc Biol 1988; 35:198-213. 10.1080/19485565.1988.9988702

174. Cutter, G. R., Zimmerman, J., Salter, A. R., Knappertz, V., Suarez, G., Waterbor, J., Howard, V. J. and Marrie, R. A. Causes of death among persons with multiple sclerosis. Mult Scler Relat Disord 2015; 4:484-490. 10.1016/j.msard.2015.07.008
175. da Silva, E. M. and e Silva, G. A. Asthma-related mortality in the city of Rio de Janeiro, Brazil, 2000-2009: A multicausal analysis. Cad Saude Publica 2013; 29:667-680. 10.1590/s0102-311x2013000400005
176. de Oliveira, B. Z., Gotlieb, S. L. D., Laurenti, R. and Jorge, M. H. P. M. Mortality due to hypertension in women: A multiple cause analysis. Revista Brasileira de Epidemiologia 2009; 12:556-565. 10.1590/s1415-790x2009000400006
177. Dembling, B. Mental disorder as a contributing cause of death in the U.S. in 1992. Psychiatr Serv 1997; 48:45. 10.1176/ps.48.1.45
178. Désesquelles, A., Demuru, E., Salvatore, M. A., Pappagallo, M., Frova, L., Meslé, F. and Egidi, V. Mortality from Alzheimer's disease, Parkinson's disease, and dementias in France and Italy: a comparison using the multiple cause-of-death approach. J Aging Health 2014; 26:283-315. 10.1177/0898264313514443
179. Désesquelles, A., Gamboni, A., Demuru, E., Barbieri, M., Denissov, G., Egidi, V., Frova, L., Pappagallo, M., Goldberger, N., Grundy, E., Marshall, C., Meslé, F., Pechholdova, M. and Sakkeus, L. We only die once. but from how many causes? Population and Societies 2016; . https://www.scopus.com/inward/record.uri?eid=2-s2.0-84978829787&partnerID=40&md5=eefdec9306c731fd93028bf4eaacd291
180. Doi, Y., Yokoyama, T., Nakamura, Y., Nagai, M., Fujimoto, K. and Nakano, I. How can the national burden of Parkinson's disease comorbidity and mortality be estimated for the Japanese population? J Epidemiol 2011; 21:211-6. 10.2188/jea.je20100149
181. Domingues, C. S. B. and Waldman, E. A. Causes of Death among People Living with AIDS in the Pre- and Post-HAART Eras in the City of S(a)over-tildeo Paulo, Brazil. PLoS One 2014; 9. 10.1371/journal.pone.0114661
182. Dorn H, M. I. Uses and significance of multiple cause tabulations for mortality statistics. AJPH 1964; 54.
183. Drapkina, O. M., Samorodskaya, I. V. and Vaisman, D. S. [Opportunities and Problems of Analysis of Mortality from Myocardial Infarction According to Medical Certificates of Death (on the Example of the Tula Region)]. [Russian]. Kardiologiia 2019; 59(7):5-10.
184. D'Souza, M. J., Li, R. C. and Wentzien, D. E. Delaware's 1999-2017 Leading Causes of Death Information Illustrates Its Obesity and Obesity-Related Life-Limiting Disease Burdens. Res Health Sci 2019; 4:327-346. 10.22158/rhs.v4n4p327
185. Duncan, M. E. and Goldacre, M. J. Mortality trends for tuberculosis and sarcoidosis in English populations, 1979-2008. Int J Tuberc Lung Dis 2012; 16:38-42. 10.5588/ijtld.11.0077
186. Duncan, M. E. and Goldacre, M. J. Certification of deaths from diabetes mellitus and obesity in England: trends into the twenty-first century. J Public Health (Oxf) 2013; 35:293-7. 10.1093/pubmed/fds074
187. Esposito, D. H., Holman, R. C., Haberling, D. L., Tate, J. E., Podewils, L. J., Glass, R. I. and Parashar, U. Baseline estimates of diarrhea-associated mortality among United States children before rotavirus vaccine introduction. Pediatr Infect Dis J 2011; 30:942-7. 10.1097/INF.0b013e3182254d19
188. Fedeli, U., Schievano, E., Lisiero, M., Avossa, F., Mastrangelo, G. and Saugo, M. Descriptive epidemiology of chronic liver disease in northeastern Italy: an analysis of multiple causes of death. Popul Health Metr 2013; 11:20. 10.1186/1478-7954-11-20
189. Fedeli, U., Schievano, E., Saugo, M. and Rodeghiero, F. Mortality from myelodysplastic syndromes: a multiple causes of death approach. Am J Hematol 2014; 89:450-1. 10.1002/ajh.23671

190. Fedeli, U., Zoppini, G., Goldoni, C. A., Avossa, F., Mastrangelo, G. and Saugo, M. Multiple causes of death analysis of chronic diseases: the example of diabetes. Popul Health Metr 2015; 13:21. 10.1186/s12963-015-0056-y
191. Fedeli, U., Piccinni, P., Schievano, E., Saugo, M. and Pellizzer, G. Growing burden of sepsis-related mortality in northeastern Italy: a multiple causes of death analysis. BMC Infect Dis 2016; 16:330. 10.1186/s12879-016-1664-2
192. Ferreira, A. F., de Souza, E. A., Lima, M. D. S., García, G. S. M., Corona, F., Andrade, E. S. N., Neto, S. A. S., Filha, C. R., Dos Reis, A. D. S., Teixeira, L. G. and Ramos, A. N., Jr. [Mortality from leprosy in highly endemic contexts: integrated temporal-spatial analysis in BrazilMortalidad por lepra en zonas de alta endemicidad: análisis espacio-temporal integrado en Brasil]. Rev Panam Salud Publica 2019; 43:e87. 10.26633/rpsp.2019.87
193. Fife, D. and Rappaport, E. What role do injuries play in the deaths of old people? Accid Anal Prev 1987; 19:225-30. 10.1016/0001-4575(87)90006-6
194. Fife, D. Injuries and deaths among elderly persons. Am J Epidemiol 1987; 126:936-41. 10.1093/oxfordjournals.aje.a114731
195. Fink, A. K., German, R. R., Heron, M., Stewart, S. L., Johnson, C. J., Finch, J. L., Yin, D. and Schaeffer, P. E. Impact of using multiple causes of death codes to compute site-specific, death certificate-based cancer mortality statistics in the United States. Cancer Epidemiol 2012; 36:22-28. 10.1016/j.canep.2011.07.004
196. Flaten, T. P. Mortality from dementia in Norway, 1969-83. J Epidemiol Community Health 1989; 43:285-9. 10.1136/jech.43.3.285
197. Fuhrman, C., Jougla, E., Nicolau, J., Eilstein, D. and Delmas, M. C. Deaths from chronic obstructive pulmonary disease in France, 1979-2002: A multiple cause analysis. Thorax 2006; 61:930-934. 10.1136/thx.2006.061267
198. Furukawa, T. S., Santo, A. H. and Mathias, T. A. Multiple causes of death related to cerebrovascular diseases in the State of Parana, Brazil. Rev Bras Epidemiol 2011; 14:231-9. 10.1590/s1415-790x2011000200005
199. Garshick, E., Kelley, A., Cohen, S. A., Garrison, A., Tun, C. G., Gagnon, D. and Brown, R. A prospective assessment of mortality in chronic spinal cord injury. Spinal Cord 2005; 43:408-16. 10.1038/sj.sc.3101729
200. Gaui, E. N., Klein, C. H. and De Oliveira, G. M. M. Mortality due to heart failure: Extended analysis and temporal trend in three states of Brazil. Arq Bras Cardiol 2010; 94:52-58.
201. Gillum, R. F. and Obisesan, T. O. Differences in mortality associated with dementia in U.S. blacks and whites. J Am Geriatr Soc 2011; 59:1823-8. 10.1111/j.1532-5415.2011.03598.x
202. Goldacre, M. J. Cause-specific mortality: understanding uncertain tips of the disease iceberg. J Epidemiol Community Health 1993; 47:491-6. 10.1136/jech.47.6.491
203. Goldacre, M. J., Duncan, M. E., Cook-Mozaffari, P. and Griffith, M. Trends in mortality rates comparing underlying-cause and multiple-cause coding in an English population 1979-1998. J Public Health Med 2003; 25:249-53. 10.1093/pubmed/fdg058
204. Goldacre, M. J., Duncan, M. E., Cook-Mozaffari, P. and Neil, H. A. Trends in mortality rates for death-certificate-coded diabetes mellitus in an English population 1979-99. Diabet Med 2004; 21:936-9. 10.1111/j.1464-5491.2004.01226.x
205. Goldacre, M. J., Duncan, M., Cook-Mozaffari, P. and Griffith, M. Mortality rates for common respiratory diseases in an English population 1979-1998: artefact and substantive trends. J Public Health (Oxf) 2004; 26:8-12. 10.1093/pubmed/fdh099
206. Goldacre, M. J., Duncan, M., Cook-Mozaffari, P. and Griffith, M. Trends in mortality for cancers, comparing multiple- and underlying-cause rates, in an English population 1979-1999. Br J Cancer 2004; 90:1019-1021. 10.1038/sj.bjc.6601633
207. Goldacre, M. J., Duncan, M., Griffith, M. and Cook-Mozaffari, P. Alcohol as a certified cause of death in a 'middle England' population 1979-1999: database study. J Public Health (Oxf) 2004; 26:343-6. 10.1093/pubmed/fdh183

208. Goldacre, M. J., Duncan, M. E., Griffith, M. and Cook-Mozaffari, P. Psychiatric disorders certified on death certificates in an English population. Soc Psychiatry Psychiatr Epidemiol 2006; 41:409-14. 10.1007/s00127-006-0035-5
209. Goldacre, M. J., Duncan, M., Cook-Mozaffari, P., Griffith, M. and Travis, S. Inflammatory bowel disease, peptic ulcer and diverticular disease as certified causes of death in an English population 1979-2003. Eur J Gastroenterol Hepatol 2008; 20:96-103. 10.1097/MEG.0b013e3282f1cbc8
210. Goldacre, M. J., Duncan, M., Griffith, M. and Rothwell, P. M. Mortality rates for stroke in England from 1979 to 2004: trends, diagnostic precision, and artifacts. Stroke 2008; 39:2197-203. 10.1161/strokeaha.107.509695
211. Goldacre, M. J., Duncan, M., Griffith, M. and Turner, M. R. Trends in death certification for multiple sclerosis, motor neuron disease, Parkinson's disease and epilepsy in English populations 1979-2006. J Neurol 2010; 257:706-15. 10.1007/s00415-009-5392-z
212. Goldacre, M. J., Duncan, M. E. and Griffith, M. Death rates for asthma in English populations 1979-2007: comparison of underlying cause and all certified causes. Public Health 2012; 126:386-93. 10.1016/j.puhe.2012.01.022
213. Goldberger, N., Applbaum, Y., Meron, J. and Haklai, Z. High Israeli mortality rates from diabetes and renal failure - Can international comparison of multiple causes of death reflect differences in choice of underlying cause? Isr J Health Policy Res 2015; 4:31. 10.1186/s13584-015-0027-6
214. Goodman, R. A., Manton, K. G., Nolan, T. F., Bregman, D. J. and Hinman, A. R. Mortality Data Analysis Using a Multiple-Cause Approach. JAMA: The Journal of the American Medical Association 1982; 247:793-796. 10.1001/jama.1982.03320310041026
215. Grande, E., Grippo, F., Frova, L., Pantosti, A., Pezzotti, P. and Fedeli, U. The increase of sepsis-related mortality in Italy: a nationwide study, 2003-2015. Eur J Clin Microbiol Infect Dis 2019; 38:1701-1708. 10.1007/s10096-019-03601-3
216. Greenlund, S. F., Croft, J. B. and Kobau, R. Epilepsy by the Numbers: Epilepsy deaths by age, race/ethnicity, and gender in the United States significantly increased from 2005 to 2014. Epilepsy Behav 2017; 69:28-30. 10.1016/j.yebeh.2017.01.016
217. Hansell, A. L., Walk, J. A. and Soriano, J. B. What do chronic obstructive pulmonary disease patients die from? A multiple cause coding analysis. Eur Respir J 2003; 22:809-14. 10.1183/09031936.03.00031403
218. Harding, J. L., Shaw, J. E., Peeters, A., Guiver, T., Davidson, S. and Magliano, D. J. Mortality trends among people with type 1 and type 2 diabetes in Australia: 1997-2010. Diabetes Care 2014; 37:2579-86. 10.2337/dc14-0096
219. Harzke, A. J., Baillargeon, J. G., Kelley, M. F., Diamond, P. M., Goodman, K. J. and Paar, D. P. HCV-related mortality among male prison inmates in Texas, 1994-2003. Ann Epidemiol 2009; 19:582-9. 10.1016/j.annepidem.2009.03.009
220. Huang, J. Y., Bristow, B., Shafir, S. and Sorvillo, F. Coccidioidomycosis-associated Deaths, United States, 1990-2008. Emerg Infect Dis 2012; 18:1723-8. 10.3201/eid1811.120752
221. Hummer, R. A., Nam, C. B. and Rogers, R. G. Adult mortality differentials associated with cigarette smoking in the USA. Population Research and Policy Review 1998; 17:285-304. 10.1023/A:1005955127544
222. Jansson, B., Johansson, L. A., Rosen, M. and Svanstrom, L. National adaptations of the ICD rules for classification--a problem in the evaluation of cause-of-death trends. Journal of Clinical Epidemiology 1997; 50(4):367-75.
223. Jansson, B. and Ahmed, N. Epilepsy and injury mortality in Sweden--the importance of changes in coding practice. Seizure 2002; 11:361-70. 10.1053/seiz.2002.0680
224. Jowching, E. Multiple cause-of-death analysis of hypertension-related mortality in New-York-State. Public Health Reports 1987; 102:329-335.
225. Kaufmann, R. B., Staes, C. J. and Matte, T. D. Deaths related to lead poisoning in the United States, 1979-1998. Environ Res 2003; 91:78-84. 10.1016/s0013-9351(02)00017-8

226. Kingwell, E., Zhu, F., Evans, C., Duggan, T., Oger, J. and Tremlett, H. Causes that Contribute to the Excess Mortality Risk in Multiple Sclerosis: A Population-Based Study. Neuroepidemiology 2020; 54:131-139. 10.1159/000504804
227. Kodadhala, V., Obi, J., Wessly, P., Mehari, A. and Gillum, R. F. Asthma-related mortality in the United States, 1999 to 2015: A multiple causes of death analysis. Ann Allergy Asthma Immunol 2018; 120:614-619. 10.1016/j.anai.2018.03.005
228. Kosan, Z., Bedir, B., Yilmaz, S., Aras, A., Calikoglu, E. O. and Ucar, M. An Evaluation of the Infant Mortality Rate in 2014 and 2015 in Northeastern Anatolia. Konuralp Tip Dergisi 2019; 11:76-81. 10.18521/ktd.430972
229. Krueger, D. E. Hypertensive and chronic respiratory disease mortality - confirmation of trends by multiple cause of death data. Public Health Reports 1966; 81:197-+. 10.2307/4592683
230. Kuller, L., Seltser, R., Paffenbarger, R. S., Jr. and Krueger, D. E. Trends in cerebrovascular disease mortality based on multiple cause tabulation of death certificates 1930-1960. A comparison of trends in Memphis and Baltimore. Am J Epidemiol 1968; 88:307-17. 10.1093/oxfordjournals.aje.a120890
231. Lanska, D. J. and Peterson, P. M. Geographic variation in the decline of stroke mortality in the United States. Stroke 1995; 26:1159-65. 10.1161/01.str.26.7.1159
232. Laribi, S., Aouba, A., Resche-Rigon, M., Johansen, H., Eb, M., Peacock, F. W., Masip, J., Ezekowitz, J. A., Cohen-Solal, A., Jougla, E., Plaisance, P. and Mebazaa, A. Trends in death attributed to myocardial infarction, heart failure and pulmonary embolism in Europe and Canada over the last decade. QJM 2014; 107:813-20. 10.1093/qjmed/hcu083
233. Larsen, J. P., Kvale, G. and Aarli, J. A. Multiple sclerosis and mortality statistics. Acta Neurol Scand 1985; 71:237-241. 10.1111/j.1600-0404.1985.tb03194.x
234. Larson, T. C., Kaye, W., Mehta, P. and Horton, D. K. Amyotrophic Lateral Sclerosis Mortality in the United States, 2011-2014. Neuroepidemiology 2018; 51:96-103. 10.1159/000488891
235. Laurenti, R. Analysis of mortality, based on basic cause of death and on multiple causes. Rev Saude Publica 1974; 8:421-435.
236. Leung, J., Bialek, S. R. and Marin, M. Trends in varicella mortality in the United States: Data from vital statistics and the national surveillance system. Hum Vaccin Immunother 2015; 11:662-8. 10.1080/21645515.2015.1008880
237. Lin, Y. P. and Lu, T. H. Trends in death rate from diabetes according to multiple-cause-of-death differed from that according to underlying-cause-of-death in Taiwan but not in the United States, 1987-2007. J Clin Epidemiol 2012; 65:572-6. 10.1016/j.jclinepi.2011.09.010
238. Lin, J. J., Liang, F. W., Li, C. Y. and Lu, T. H. Leading causes of death among decedents with mention of schizophrenia on the death certificates in the United States. Schizophr Res 2018; 197:116-123. 10.1016/j.schres.2018.01.011
239. Lindahl, B. I. and Johansson, L. A. Multiple cause-of-death data as a tool for detecting artificial trends in the underlying cause statistics: a methodological study. Scand J Soc Med 1994; 22:145-58.
240. Lu, T. H., Walker, S., Johansson, L. A. and Huang, C. N. An international comparison study indicated physicians' habits in reporting diabetes in part I of death certificate affected reported national diabetes mortality. J Clin Epidemiol 2005; 58:1150-7. 10.1016/j.jclinepi.2005.03.009
241. Lu, T. H., Hsu, P. Y., Bjorkenstam, C. and Anderson, R. N. Certifying diabetes-related cause-of-death: a comparison of inappropriate certification statements in Sweden, Taiwan and the USA. Diabetologia 2006; 49:2878-81. 10.1007/s00125-006-0470-6
242. Lu, T. H. and Lin, J. J. Using multiple-cause-of-death data as a complement of underlying-cause-of-death data in examining mortality differences in psychiatric disorders between countries. Soc Psychiatry Psychiatr Epidemiol 2010; 45:837-42. 10.1007/s00127-009-0127-0
243. Lu, T. H., Hsiao, A., Chang, P. C., Chao, Y. C., Hsu, C. C., Peng, H. C., Chen, L. H. and Kawachi, I. Counting injury deaths: a comparison of two definitions and two countries. Inj Prev 2015; 21:e127-32. 10.1136/injuryprev-2013-040974

244. Madsen, M., Davidsen, M., Rasmussen, S., Abildstrom, S. Z. and Osler, M. The validity of the diagnosis of acute myocardial infarction in routine statistics: a comparison of mortality and hospital discharge data with the Danish MONICA registry. J Clin Epidemiol 2003; 56:124-30. 10.1016/s0895-4356(02)00591-7
245. Makela, P. Alcohol-related mortality by age and sex and its impact on life expectancy - Estimates based on the Finnish death register. Eur J Public Health 1998; 8:43-51. 10.1093/eurpub/8.1.43
246. Manderbacka, K., Arffman, M., Lyytikainen, O., Sajantila, A. and Keskimaki, I. What really happened with pneumonia mortality in Finland in 2000-2008?: a cohort study. Epidemiol Infect 2013; 141:800-4. 10.1017/S0950268812001562
247. Mannino, D. M., Brown, C. and Giovino, G. A. Obstructive lung disease deaths in the United States from 1979 through 1993. An analysis using multiple-cause mortality data. Am J Respir Crit Care Med 1997; 156:814-8. 10.1164/ajrccm.156.3.9702026
248. Manton, K. G. Sex and race specific mortality differentials in multiple cause of death data. Gerontologist 1980; 20:480-93. 10.1093/geront/20.4.480
249. Manton, K. G., Stallard, E. and Poss, S. S. Estimates of U.S. multiple cause life tables. Demography 1980; 17:85-102.
250. Manton, K. G. and Stallard, E. Temporal trends in U. S. multiple cause of death mortality data: 1968 to 1977. Demography 1982; 19:527-47.
251. Manton, K. G. B., H. M. CVD mortality, 1968-1978: observations and implications. Stroke 1984; .
252. Manton, K. G. Cause specific mortality patterns among the oldest old: multiple cause of death trends 1968 to 1980. J Gerontol 1986; 41:282-9. 10.1093/geronj/41.2.282
253. Manton, K. G. and Myers, G. C. Recent trends in multiple-caused mortality 1968 to 1982: Age and cohort components. Population Research and Policy Review 1987; 6:161-176. 10.1007/BF00149207
254. Manton, K. G., Wrigley, J. M., Cohen, H. J. and Woodbury, M. A. Cancer mortality, aging, and patterns of comorbidity in the United States: 1968 to 1986. J Gerontol 1991; 46:S225-34. 10.1093/geronj/46.4.s225
255. Marin, M., Zhang, J. X. and Seward, J. F. Near elimination of varicella deaths in the US after implementation of the vaccination program. Pediatrics 2011; 128:214-20. 10.1542/peds.2010-3385
256. Martins-Melo, F. R., Ramos, A. N., Alencar, C. H. and Heukelbach, J. Mortality related to Chagas disease and HIV/AIDS coinfection in Brazil. Journal of Tropical Medicine 2012; . 10.1155/2012/534649
257. Martins-Melo, F. R., da Silveira Lima, M., Alencar, C. H., Ramos Jr, A. N. and Heukelbach, J. Epidemiological patterns of mortality due to visceral leishmaniasis and HIV/AIDS co-infection in Brazil, 2000-2011. Transactions of the Royal Society of Tropical Medicine and Hygiene 2014; 108:338-347. 10.1093/trstmh/tru050
258. Martins, E. F., Almeida, P. F., Paixão, C. O., Bicalho, P. G. and Errico, L. S. [Multiple causes of maternal mortality related to abortion in Minas Gerais State, Brazil, 2000-2011]. Cad Saude Publica 2017; 33:e00133115. 10.1590/0102-311x00133116
259. Martins-Melo, F. R., Ramos, A. N., Alencar, C. H., Lima, M. S. and Heukelbach, J. Epidemiology of soil-transmitted helminthiases-related mortality in Brazil. Parasitology 2017; . 10.1017/S0031182016002341
260. Martins-Melo, F. R., Alencar, C. H., Ramos, A. N., Jr. and Heukelbach, J. Epidemiology of mortality related to Chagas' disease in Brazil, 1999-2007. PLoS Negl Trop Dis 2012; 6:e1508. 10.1371/journal.pntd.0001508
261. Martins-Melo, F. R., Pinheiro, M. C., Ramos, A. N., Jr., Alencar, C. H., Bezerra, F. S. and Heukelbach, J. Trends in schistosomiasis-related mortality in Brazil, 2000-2011. Int J Parasitol 2014; 44:1055-62. 10.1016/j.ijpara.2014.07.009
262. Maudsley, G., Hutton, J. L. and Pharoah, P. O. Cause of death in cerebral palsy: a descriptive study. Arch Dis Child 1999; 81:390-4. 10.1136/adc.81.5.390

263. McCoy, L., Sorvillo, F. and Simon, P. Varicella-related mortality in California, 1988-2000. Pediatr Infect Dis J 2004; 23:498-503. 10.1097/01.inf.0000129684.27717.d6
264. McCoy, L., Redelings, M., Sorvillo, F. and Simon, P. A multiple cause-of-death analysis of asthma mortality in the United States, 1990-2001. J Asthma 2005; 42:757-63. 10.1080/02770900500308189
265. Meyers, D. J., Hood, M. E. and Stopka, T. J. HIV and hepatitis C mortality in Massachusetts, 2002-2011: spatial cluster and trend analysis of HIV and HCV using multiple cause of death. PLoS One 2014; 9:e114822. 10.1371/journal.pone.0114822
266. Midgard, R., Albrektsen, G., Riise, T., Kvale, G. and Nyland, H. Prognostic factors for survival in multiple sclerosis: a longitudinal, population based study in More and Romsdal, Norway. Journal of Neurology, Neurosurgery & Psychiatry. 58(4):417-21, 1995 Apr. 1995; 58(4):417-21.
267. Milham, S., Jr. Using multiple cause of death coding in occupational mortality studies. Am J Ind Med 1988; 14:341-4. 10.1002/ajim.4700140311
268. Moussa, M. A., El Sayed, A. M., Sugathan, T. N., Khogali, M. M. and Verma, D. Analysis of underlying and multiple-cause mortality data. Genus 1992; 48:89-105.
269. Murdoch, D. R., Love, M. P., Robb, S. D., McDonagh, T. A., Davie, A. P., Ford, I., Capewell, S., Morrison, C. E. and McMurray, J. J. Importance of heart failure as a cause of death. Changing contribution to overall mortality and coronary heart disease mortality in Scotland 1979-1992. Eur Heart J 1998; 19:1829-35. 10.1053/euhj.1998.1269
270. Nembhard, W. N., Pathak, E. B. and Schocken, D. D. Racial/ethnic disparities in mortality related to congenital heart defects among children and adults in the United States. Ethn Dis 2008; 18:442-9.
271. Newman, J. M., DeStefano, F., Valway, S. E., German, R. R. and Muneta, B. Diabetes-associated mortality in Native Americans. Diabetes Care 1993; 16:297-9. 10.2337/diacare.16.1.297
272. Ning, P., Schwebel, D. C., Chu, H., Zhu, M. and Hu, G. Changes in reporting for unintentional injury deaths, United States of America. Bull World Health Organ 2019; 97:190-199. 10.2471/blt.18.215327
273. Nordstrom, D. L., Yokoi-Shelton, M. L. and Zosel, A. Using multiple cause-of-death data to improve surveillance of drug-related mortality. J Public Health Manag Pract 2013; 19:402-11. 10.1097/PHH.0b013e318271c622
274. Obi, J., Mehari, A. and Gillum, R. Mortality Related to Chronic Obstructive Pulmonary Disease and Co-morbidities in the United States, A Multiple Causes of Death Analysis. Copd 2018; 15:200-205. 10.1080/15412555.2018.1454897
275. Ogundipe, F., Kodadhala, V., Ogundipe, T., Mehari, A. and Gillum, R. Disparities in Sepsis Mortality by Region, Urbanization, and Race in the USA: a Multiple Cause of Death Analysis. J Racial Ethn Health Disparities 2019; 6:546-551. 10.1007/s40615-018-00553-w
276. Ogundipe, F., Mehari, A. and Gillum, R. Disparities in Sarcoidosis Mortality by Region, Urbanization, and Race in the United States: A Multiple Cause of Death Analysis. Am J Med 2019; 132:1062-1068.e3. 10.1016/j.amjmed.2019.03.048
277. Olie, V., Fuhrman, C., Chin, F., Lamarche-Vadel, A., Scarabin, P. Y. and de Peretti, C. Time trends in pulmonary embolism mortality in France, 2000-2010. Thromb Res 2015; 135:334-8. 10.1016/j.thromres.2014.12.002
278. Olson, F. E., Hammes, L. M., Shipley, P. W. and Norris, F. D. A study of multiple causes of death in California. J Chronic Dis 1962; 15:157-&. 10.1016/0021-9681(62)90065-6
279. Peden, A. E., Franklin, R. C., Mahony, A. J., Scarr, J. and Barnsley, P. D. Using a retrospective cross-sectional study to analyse unintentional fatal drowning in Australia: ICD-10 coding-based methodologies verses actual deaths. BMJ Open 2017; 7:e019407. 10.1136/bmjopen-2017-019407
280. Perazzo, H., Pacheco, A. G., De Boni, R., Luz, P. M., Fittipaldi, J., Cardoso, S. W., Grinsztejn, B. and Veloso, V. G. Age-Standardized Mortality Rates Related to Cirrhosis in Brazil from 2000 to 2012: A Nationwide Analysis. Annals of Hepatology 2017; 16:269-278. 10.5604/16652681.1231586

281. Perkins, B. A., Flood, J. M., Danila, R., Holman, R. C., Reingold, A. L., Klug, L. A., Virata, M., Cieslak, P. R., Zaki, S. R., Pinner, R. W., Khabbaz, R. F., Rothrock, G., Vugia, D., Hadler, J., Cartter, M., Meek, J., Ryder, R., Wilson, M., Osterholm, M., MacDonald, K. L., Rainbow, J., Crouch, N., LeDell, K., Fleming, D., Hedberg, K., Brenner, D., Eberhard, M., Olson, J., Rollin, P. and Parrish, R. G. Unexplained Deaths Due to Possibly Infectious Causes in the United States: Defining the Problem and Designing Surveillance and Laboratory Approaches. Emerg Infect Dis 1996; 2:47-53. 10.3201/eid0201.960106
282. Pickard, A. S., Jung, E., Bartle, B., Weiss, K. B. and Lee, T. A. Coded cause of death and timing of COPD diagnosis. Copd 2009; 6:41-7. 10.1080/15412550802587935
283. Polednak, A. P. Recent decline in the U.S. death rate from myeloproliferative neoplasms, 1999-2006. Cancer Epidemiol 2012; 36:133-6. 10.1016/j.canep.2011.05.016
284. Polednak, A. P. US regional differences in death rates from depression. Soc Psychiatry Psychiatr Epidemiol 2012; 47:1977-83. 10.1007/s00127-012-0503-z
285. Polednak, A. P. Surveillance of US death rates from chronic diseases related to excessive alcohol use. Alcohol and Alcoholism 2015; 51:54-62. 10.1093/alcalc/agv056
286. Prati, C., Puyraveau, M., Guillot, X., Verhoeven, F. and Wendling, D. Deaths associated with ankylosing spondylitis in France from 1969 to 2009. Journal of Rheumatology 2017; 44:594-598. 10.3899/jrheum.160942
287. Quinones, A., Lobach, I., Maduro, G. A., Jr., Smilowitz, N. R. and Reynolds, H. R. Diabetes and ischemic heart disease death in people age 25-54: a multiple-cause-of-death analysis based on over 400?000 deaths from 1990 to 2008 in New York City. Clin Cardiol 2015; 38:114-20. 10.1002/clc.22367
288. Rahimi, K., Duncan, M., Pitcher, A., Emdin, C. A. and Goldacre, M. J. Mortality from heart failure, acute myocardial infarction and other ischaemic heart disease in England and Oxford: a trend study of multiple-cause-coded death certification. Journal of Epidemiology & Community Health 2015; 69(10):1000-5.
289. Rao, C., Adair, T., Bain, C. and Doi, S. A. Mortality from diabetic renal disease: a hidden epidemic. Eur J Public Health 2012; . 10.1093/eurpub/ckq205
290. Richardson, D. B. Use of multiple cause of death data in cancer mortality analyses. Am J Ind Med 2006; 49:683-9. 10.1002/ajim.20343
291. Rinsky, J. L., Hoppin, J. A., Blair, A., He, K., Beane Freeman, L. E. and Chen, H. Agricultural exposures and stroke mortality in the Agricultural Health Study. J Toxicol Environ Health A 2013; 76:798-814. 10.1080/15287394.2013.819308
292. Rio, E. M., Gallo, P. R. and Reis, A. O. [Asthma mortality in the Municipality of São Paulo (1993-1995): analysis by multiple cause of death]. Cad Saude Publica 2003; 19:1541-4. 10.1590/s0102-311x2003000500033
293. Rodriguez, F., Blum, M. R., Falasinnu, T., Hastings, K. G., Hu, J., Cullen, M. R. and Palaniappan, L. P. Diabetes-attributable mortality in the United States from 2003 to 2016 using a multiple-cause-of-death approach. Diabetes Res Clin Pract 2019; 148:169-178. 10.1016/j.diabres.2019.01.015
294. Romon, I., Jougla, E., Balkau, B. and Fagot-Campagna, A. The burden of diabetes-related mortality in France in 2002: an analysis using both underlying and multiple causes of death. Eur J Epidemiol 2008; 23:327-34. 10.1007/s10654-008-9235-5
295. Rosenwax, L., McNamara, B. and Zilkens, R. A population-based retrospective cohort study comparing care for Western Australians with and without Alzheimer's disease in the last year of life. Health and Social Care in the Community 2009; 17:36-44. 10.1111/j.1365-2524.2008.00795.x
296. Sacks, J. J., Helmick, C. G. and Langmaid, G. Deaths from arthritis and other rheumatic conditions, United States, 1979-1998. J Rheumatol 2004; 31:1823-8.
297. Santo, A. H. [Chickenpox-related mortality trends in the state of Sao Paulo, Brazil, 1985-2004: a multiple cause approach]. Rev Panam Salud Publica 2007; 22:132-40. 10.1590/s1020-49892007000700008

298. Santo, A. H. Cysticercosis-related mortality in the State of Sao Paulo, Brazil, 1985-2004: A study using multiple causes of death. Cadernos De Saude Publica 2007; 23:2917-2927. Doi 10.1590/S0102-311x2007001200013
299. Santo, A. H. [Epidemiological potential of multiple-cause-of-death data listed on death certificates, Brazil, 2003]. Rev Panam Salud Publica 2007; 22:178-86. 10.1590/s1020-49892007000800004
300. Santo, A. H., Puech-Leao, P. and Krutman, M. Trends in aortic aneurysm- and dissection-related mortality in the state of Sao Paulo, Brazil, 1985-2009: multiple-cause-of-death analysis. BMC Public Health 2012; 12:859. 10.1186/1471-2458-12-859
301. Sejvar, J. J., Holman, R. C., Bresee, J. S., Kochanek, K. D. and Schonberger, L. B. Amyotrophic lateral sclerosis mortality in the United States, 1979-2001. Neuroepidemiology 2005; 25:144-52. 10.1159/000086679
302. Selik, R. M. and Rabkin, C. S. Cancer death rates associated with human immunodeficiency virus infection in the United States. J Natl Cancer Inst 1998; 90:1300-1302. 10.1093/jnci/90.17.1300
303. Sheth, S. G., Krauss, G., Krumholz, A. and Li, G. Mortality in epilepsy: Driving fatalities vs other causes of death in patients with epilepsy. Neurology 2004; 63:1002-1007. 10.1212/01.WNL.0000138590.00074.9A
304. Simmons, R., Ireland, G., Ijaz, S., Ramsay, M. and Mandal, S. Causes of death among persons diagnosed with hepatitis C infection in the pre- and post-DAA era in England: A record linkage study. J Viral Hepat 2019; 26:873-880. 10.1111/jvh.13096
305. Sjogren, H., Eriksson, A. and Ahlm, K. Role of alcohol in unnatural deaths: a study of all deaths in Sweden. Alcoholism: Clinical & Experimental Research 2000; 24(7):1050-6.
306. Sjogren, H., Valverius, P. and Eriksson, A. Gender differences in role of alcohol in fatal injury events. Eur J Public Health 2006; 16:266-270. 10.1093/eurpub/ckl039
307. Smestad, C., Sandvik, L. and Celius, E. G. Excess mortality and cause of death in a cohort of Norwegian multiple sclerosis patients. Mult Scler 2009; 15:1263-70. 10.1177/1352458509107010
308. Smith, C. A. and Barnett, E. Diabetes-related mortality among Mexican Americans, Puerto Ricans, and Cuban Americans in the United States. Rev Panam Salud Publica 2005; 18:381-7. 10.1590/s1020-49892005001000001
309. Smithers-Sheedy, H., Raynes-Greenow, C., Badawi, N., Khandaker, G., Menzies, R. and Jones, C. A. Cytomegalovirus-related childhood mortality in Australia 1999-2011. J Paediatr Child Health 2015; 51:901-5. 10.1111/jpc.12896
310. Snowdon, D. A. and Phillips, R. L. Does a vegetarian diet reduce the occurrence of diabetes? Am J Public Health 1985; 75:507-12. 10.2105/ajph.75.5.507
311. Sondermeyer, G. L., Lee, L. A., Gilliss, D. and Vugia, D. J. Coccidioidomycosis-Associated Deaths in California, 2000-2013. Public Health Rep 2016; 131:531-5. 10.1177/0033354916662210
312. Sosin, D. M., Sacks, J. J. and Smith, S. M. Head Injury-Associated Deaths in the United States From 1979 to 1986. JAMA: The Journal of the American Medical Association 1989; 262:2251-2255. 10.1001/jama.1989.03430160073033
313. Steenland, K., Steenland, K., Nowlin, S., Nowlin, S., Adams, S., Ryan, B. and Adams, S. Use of multiple-cause mortality data in epidemiologic analyses: US rate and proportion files developed by the National Institute for Occupational Safety and Health and the National Cancer Institute. American Journal of Epidemiology 1992; 136:855-862. 10.1093/aje/136.7.855
314. Steenland, K., Attfield, M. and Mannejte, A. Pooled analyses of renal disease mortality and crystalline silica exposure in three cohorts. Annals of Occupational Hygiene 2002; 46:4-9. 10.1093/annhyg/46.suppl_1.4
315. Suligoi, B., Virdone, S., Taborelli, M., Frova, L., Grande, E., Grippo, F., Pappagallo, M., Regine, V., Pugliese, L., Serraino, D. and Zucchetto, A. Excess mortality related to circulatory system diseases and diabetes mellitus among Italian AIDS patients vs. non-AIDS population: a population-based

cohort study using the multiple causes-of-death approach. Bmc Infectious Diseases 2018; 18. ARTN 428
316. García Tardón, A., Zaplana Piñeiro, J., Hernández Mejías, R. and Cueto Espinar, A. Estudio de las Causas Múltiples de Defunción en Asturias, 1988. Gaceta Sanitaria 1993; . 10.1016/s0213-9111(93)71137-1
317. Thomas, S. L., Griffiths, C., Smeeth, L., Rooney, C. and Hall, A. J. Burden of mortality associated with autoimmune diseases among females in the United Kingdom. Am J Public Health 2010; 100:2279-87. 10.2105/ajph.2009.180273
318. To, T., Simatovic, J., Zhu, J., Feldman, L., Dell, S. D., Lougheed, M. D., Licskai, C. and Gershon, A. Asthma deaths in a large provincial health system. A 10-year population-based study. Ann Am Thorac Soc 2014; 11:1210-7. 10.1513/AnnalsATS.201404-138OC
319. Todd, S., Barr, S. and Passmore, A. P. Cause of death in Alzheimer's disease: a cohort study. QJM 2013; 106:747-53. 10.1093/qjmed/hct103
320. Trias-Llimos, S., Martikainen, P., Makela, P. and Janssen, F. Comparison of different approaches for estimating age-specific alcohol-attributable mortality: The cases of France and Finland. PLoS One 2018; 13:e0194478. 10.1371/journal.pone.0194478
321. Tu, E. J. Multiple cause-of-death analysis of hypertension-related mortality in New York State. Public Health Rep 1987; 102:329-35.
322. Vandormael, S., Meirschaert, A., Steyaert, J. and De Lepeleire, J. Insights on dying, dementia and death certificates. Archives of Public Health 2018; 76. 10.1186/s13690-018-0263-7
323. Veazie, M., Ayala, C., Schieb, L., Dai, S., Henderson, J. A. and Cho, P. Trends and disparities in heart disease mortality among American Indians/Alaska Natives, 1990-2009. Am J Public Health 2014; 104:S359-S367. 10.2105/AJPH.2013.301715
324. Villela, P. B., Klein, C. H. and Oliveira, G. M. M. Cerebrovascular and hypertensive diseases as multiple causes of death in Brazil from 2004 to 2013. Public Health 2018; 161:36-42. 10.1016/j.puhe.2018.04.006
325. Wall, M. M., Huang, J., Oswald, J. and McCullen, D. Factors associated with reporting multiple causes of death. BMC Med Res Methodol 2005; 5:4. 10.1186/1471-2288-5-4
326. Ward, M., May, P., Briggs, R., McNicholas, T., Normand, C., Kenny, R. A. and Nolan, A. Linking death registration and survey data: Procedures and cohort profile for The Irish Longitudinal Study on Ageing. HRB Open Res 2020; 3:43. 10.12688/hrbopenres.13083.1
327. Westerling, R. Small-Area Variation in Multiple Causes of Death in Sweden - a Comparison with Underlying Causes of Death. International Journal of Epidemiology 1995; 24:552-558. DOI 10.1093/ije/24.3.552
328. Weir. Diabetes Mortality In Rhode Island: Comparing Underlying Cause of Death versus Any Listed Cause of Death. Medicine & Health Rhode Island 2008; .
329. Wild, S. H., Bryden, J. R., Lee, R. J., Bishop, J. L., Finlayson, A. R., Byrne, C. D. and Brewster, D. H. Cancer, cardiovascular disease and diabetes mortality among women with a history of endometrial cancer. Br J Cancer 2007; 96:1747-9. 10.1038/sj.bjc.6603761
330. Williams, P. T. Fifty-three year follow-up of coronary heart disease versus HDL2 and other lipoproteins in Gofman's Livermore Cohort. J Lipid Res 2012; 53:266-72. 10.1194/jlr.M019356
331. Wing, S. and Manton, K. G. A multiple cause of death analysis of hypertension-related mortality in North Carolina, 1968-1977. Am J Public Health 1981; 71:823-30. 10.2105/ajph.71.8.823
332. Wing, S. and Manton, K. G. The contribution of hypertension to mortality in the US: 1968, 1977. American Journal of Public Health 1983; 73(2):140-4.
333. Wise, M. E. and Sorvillo, F. Hepatitis A--related mortality in California, 1989-2000: analysis of multiple cause-coded death data. Am J Public Health 2005; 95:900-5. 10.2105/ajph.2003.035485
334. Wise, M., Bialek, S., Finelli, L., Bell, B. P. and Sorvillo, F. Changing trends in hepatitis C-related mortality in the United States, 1995-2004. Hepatology 2008; 47:1128-35. 10.1002/hep.22165

335. Wrigley, J. M. and Nam, C. B. Underlying Versus Multiple Causes of Death - Effects on Interpreting Cancer Mortality Differentials by Age, Sex, and Race. Population Research and Policy Review 1987; 6:149-160. Doi 10.1007/Bf00149206
336. Yamamura, M., Santos-Neto, M., dos Santos, R. A. N., Garcia, M. C. C., Nogueira, J. A. and Arcêncio, R. A. Epidemiological characteristics of cases of death from tuberculosis and vulnerable territories. Revista Latino-Americana de Enfermagem 2015; 23:910-918. 10.1590/0104-1169.0450.2631
337. Yang, Q., Khoury, M. J. and Mannino, D. Trends and patterns of mortality associated with birth defects and genetic diseases in the United States, 1979-1992: an analysis of multiple-cause mortality data. Genet Epidemiol 1997; 14:493-505. 10.1002/(sici)1098-2272(1997)14:5<493::Aid-gepi4>3.0.Co;2-2
338. Yoon, Y. H., Yi, H. Y. and Thomson, P. C. Alcohol-related and viral hepatitis C-related cirrhosis mortality among Hispanic subgroups in the United States, 2000-2004. Alcohol Clin Exp Res 2011; 35:240-9. 10.1111/j.1530-0277.2010.01340.x
339. Zhu, M., Li, J., Li, Z., Luo, W., Dai, D., Weaver, S. R., Stauber, C., Luo, R. and Fu, H. Mortality rates and the causes of death related to diabetes mellitus in Shanghai Songjiang District: an 11-year retrospective analysis of death certificates. BMC Endocr Disord 2015; 15:45. 10.1186/s12902-015-0042-1
340. Zilkens, R. R., Spilsbury, K., Bruce, D. G. and Semmens, J. B. Linkage of hospital and death records increased identification of dementia cases and death rate estimates. Neuroepidemiology 2009; 32:61-9. 10.1159/000170908
341. Zoppini, G., Fedeli, U., Schievano, E., Dauriz, M., Targher, G., Bonora, E. and Corti, M. C. Mortality from infectious diseases in diabetes. Nutr Metab Cardiovasc Dis 2018; 28:444-450. 10.1016/j.numecd.2017.12.007
342. Moreno-Betancur, M., Sadaoui, H., Piffaretti, C. and Rey, G. Survival Analysis with Multiple Causes of Death Extending the Competing Risks Model. Epidemiology 2017; 28:12-9. 10.1097/Ede.0000000000000531
343. Piffaretti, C., Moreno-Betancur, M., Lamarche-Vadel, A. and Rey, G. Quantifying cause-related mortality by weighting multiple causes of death. Bull World Health Organ 2016; 94:870-879. 10.2471/blt.16.172189
344. Adair, T. and Lopez, A. D. The role of overweight and obesity in adverse cardiovascular disease mortality trends: an analysis of multiple cause of death data from Australia and the USA. BMC Med 2020; 18:199. 10.1186/s12916-020-01666-y
345. Barbieri, M., Désesquelles, A., Egidi, V., Demuru, E., Frova, L., Meslé, F. and Pappagallo, M. Obesity-related mortality in France, Italy, and the United States: a comparison using multiple cause-of-death analysis. Int J Public Health 2017; 62:623-629. 10.1007/s00038-017-0978-1
346. Egidi, V., Salvatore, M. A., Rivellini, G. and D'Angelo, S. A network approach to studying cause-of-death interrelations. Demogr Res 2018; 38:373-400. 10.4054/DemRes.2018.38.16
347. Axtell, C. D., Ward, E. M., McCabe, G. P., Schulte, P. A., Stern, F. B. and Glickman, L. T. Underlying and multiple cause mortality in a cohort of workers exposed to aromatic amines. Am J Ind Med 1998; 34:506-11. 10.1002/(sici)1097-0274(199811)34:5<506::aid-ajim12>3.0.co;2-5
348. Ball, L. B., Macdonald, S. C., Mott, J. A. and Etzel, R. A. Carbon monoxide-related injury estimation using ICD-coded data: methodologic implications for public health surveillance. Arch Environ Occup Health 2005; 60:119-27. 10.3200/aeoh.60.3.119-127
349. Batty, G. D., Gale, C. R., Kivimaki, M. and Bell, S. Assessment of Relative Utility of Underlying vs Contributory Causes of Death. JAMA Netw Open 2019; 2:e198024. 10.1001/jamanetworkopen.2019.8024
350. Carrillo-Larco, R. M. and Bernabe-Ortiz, A. A divergence between underlying and final causes of death in selected conditions: an analysis of death registries in Peru. PeerJ 2018; 6:e5948. 10.7717/peerj.5948

351. Chamblee, R. F., Evans, M. C., Patten, D. G. and Pearce, J. S. Injuries causing death: Their nature, external causes, and associated diseases. J Safety Res 1983; 14:21-35. 10.1016/0022-4375(83)90004-X
352. Chang, C. Y., Lu, T. H. and Cheng, T. J. Trends in reporting injury as a cause of death among people with epilepsy in the U.S., 1981-2010. Seizure 2014; 23:836-43. 10.1016/j.seizure.2014.07.002
353. Chazal, T., Lhote, R., Rey, G., Haroche, J., Eb, M., Amoura, Z. and Aubart, F. C. Giant-cell arteritis-related mortality in France: A multiple-cause-of-death analysis. Autoimmun Rev 2018; 17:1219-1224. 10.1016/j.autrev.2018.06.012
354. Chitty, K. M., Schumann, J. L., Moran, L. L., Chong, D. G., Hurzeler, T. P. and Buckley, N. A. Reporting of alcohol as a contributor to death in Australian national suicide statistics and its relationship to post-mortem alcohol concentrations. Addiction ; . 10.1111/add.15180
355. Chu, S. Y., Buehler, J. W., Oxtoby, M. J. and Kilbourne, B. W. Impact of the human immunodeficiency virus epidemic on mortality in children, United States. Pediatrics 1991; 87:806-810.
356. Cohen, J. and Steinitz, R. Underlying and contributory causes of death of adult males in two districts. J Chronic Dis 1969; 22:17-24. 10.1016/0021-9681(69)90083-6
357. Ducci, R. D., Cirino, R. H., de Oliveira, R. A., Twardowschy, C. A., Chomatas, E. R. and Kowacs, P. A. Analysis of 621 death certificates issued from 1998 to 2007 in Curitiba, Brazil, mentioning epilepsy, epileptic seizures and/or status epilepticus. Seizure 2011; 20:406-8. 10.1016/j.seizure.2011.01.012
358. Fazito, E., Vasconcelos, A. M., Pereira, M. G. and Rezende, D. F. Trends in non-AIDS-related causes of death among adults with HIV/AIDS, Brazil, 1999 to 2010. Cad Saude Publica 2013; 29:1644-53. 10.1590/0102-311x00128912
359. Fedeli, U., Avossa, F., Guzzinati, S., Bovo, E. and Saugo, M. Trends in mortality from chronic liver disease. Ann Epidemiol 2014; 24:522-6. 10.1016/j.annepidem.2014.05.004
360. Fedeli, U., Schievano, E., Targher, G., Bonora, E., Corti, M. C. and Zoppini, G. Estimating the real burden of cardiovascular mortality in diabetes. Eur Rev Med Pharmacol Sci 2019; 12:6700-6706. 10.26355/eurrev_201908_18561
361. Fingerhut, L. A. and Warner, M. The ICD-10 injury mortality diagnosis matrix. Inj Prev 2006; 12:24-9. 10.1136/ip.2005.009076
362. Fuller, J. H., Elford, J., Goldblatt, P. and Adelstein, A. M. Diabetes mortality: new light on an underestimated public health problem. Diabetologia 1983; 24:336-41. 10.1007/bf00251820
363. González-Mariño, M. A. [Causes of death from breast cancer in Colombia]. Rev Salud Publica (Bogota) 2016; 18:344-353. 10.15446/rsap.v18n3.30483
364. Gravensteen, I. K., Ekeberg, O., Thiblin, I., Helweg-Larsen, K., Hem, E., Rogde, S. and Tollefsen, I. M. Psychoactive substances in natural and unnatural deaths in Norway and Sweden - a study on victims of suicide and accidents compared with natural deaths in psychiatric patients. BMC Psychiatry 2019; 19:33. 10.1186/s12888-019-2015-9
365. Hansen, J., Asberg, S., Kumlien, E. and Zelano, J. Cause of death in patients with poststroke epilepsy: Results from a nationwide cohort study. PLoS One 2017; 12:e0174659. 10.1371/journal.pone.0174659
366. Jerschow, E., Lin, R. Y., Scaperotti, M. M. and McGinn, A. P. Fatal anaphylaxis in the United States, 1999-2010: temporal patterns and demographic associations. J Allergy Clin Immunol 2014; 134:1318-1328 e7. 10.1016/j.jaci.2014.08.018
367. Kandel, D. B., Hu, M. C., Griesler, P. and Wall, M. Increases from 2002 to 2015 in prescription opioid overdose deaths in combination with other substances. Drug Alcohol Depend 2017; 178:501-511. 10.1016/j.drugalcdep.2017.05.047
368. Karkkainen, M., Nurmi, H., Kettunen, H. P., Selander, T., Purokivi, M. and Kaarteenaho, R. Underlying and immediate causes of death in patients with idiopathic pulmonary fibrosis. BMC Pulm Med 2018; 18:69. 10.1186/s12890-018-0642-4

369. Kramarow, E., Warner, M. and Chen, L. H. Food-related choking deaths among the elderly. Injury Prevention 2014; 20:200-203. 10.1136/injuryprev-2013-040795
370. Krapfl, H. R., Gohdes, D. M. and Croft, J. B. Racial and ethnic differences in premature heart disease deaths in New Mexico: what is the role of diabetes? Ethn Dis 2006; 16:85-8.
371. Lahti, R. A., Korpi, H. and Vuori, E. Blood-positive illicit-drug findings: implications for cause-of-death certification, classification and coding. Forensic Sci Int 2009; 187:14-8. 10.1016/j.forsciint.2009.02.007
372. Landes, S. D., Stevens, J. D. and Turk, M. A. Cause of death in adults with Down syndrome in the United States. Disabil Health J 2020; . 10.1016/j.dhjo.2020.100947
373. Lanska, D. J., Lanska, M., Lavine, L. and Schoenberg, B. S. Conditions Associated with Huntington's Disease at Death: A Case-Control Study. Archives of Neurology 1988; 45:878-880. 10.1001/archneur.1988.00520320068017
374. Ly, K. N., Xing, J., Klevens, R. M., Jiles, R. B. and Holmberg, S. D. Causes of death and characteristics of decedents with viral hepatitis, United States, 2010. Clinical Infectious Diseases 2014; 58:40-49. 10.1093/cid/cit642
375. Mackenbach, J. P., Kunst, A. E., Lautenbach, H., Oei, Y. B. and Bijlsma, F. Gains in life expectancy after elimination of major causes of death: Revised estimates taking into account the effect of competing causes. J Epidemiol Community Health 1999; 53:32-37. 10.1136/jech.53.1.32
376. Manton, K. G., Tolley, D. H. and Poss, S. S. Life table techniques for multiple-cause mortality. Demography 1976; 13:541-64.
377. Manton, K. G. and Sharon, S. P. Effects of dependency among causes of death fro elimination life table strategies. Demography 1979; 16:313-327.
378. Manton, K. G. and Stallard, E. Mortality of the chronically impaired. Demography 1980; 17:189-206.
379. Martikainen, P., Makela, P., Peltonen, R. and Myrskyla, M. Income differences in life expectancy: the changing contribution of harmful consumption of alcohol and smoking. Epidemiology 2014; 25(2):182-90.
380. McDonnell, W. F., Nishino-Ishikawa, N., Petersen, F. F., Chen, L. H. and Abbey, D. E. Relationships of mortality with the fine and coarse fractions of long-term ambient PM10 concentrations in nonsmokers. Journal of Exposure Analysis and Environmental Epidemiology 2000; 10(5):427-36.
381. Mendlein, J. M., Sattin, R. W., Waxweiler, R. J., Lui, K. J. and McGee, D. L. Fall Mortality and Related Medical Conditions in the Elderly: The Association with Pulmonary Embolism. J Aging Health 1990; 2:326-340. 10.1177/089826439000200303
382. Moussa, M. A. Analysis of underlying and multiple-cause mortality data: the life table methods. Comput Methods Programs Biomed 1987; 24:3-19. 10.1016/0169-2607(87)90060-5
383. Okuda, T., Wang, Z., Lapan, S. and Fowler, D. R. Bathtub drowning: An 11-year retrospective study in the state of Maryland. Forensic Sci Int 2015; 253:64-70. 10.1016/j.forsciint.2015.05.013
384. Pacheco, A. G., Saraceni, V., Tuboi, S. H., Lauria, L. M., Moulton, L. H., Faulhaber, J. C., King, B., Golub, J. E., Durovni, B., Cavalcante, S., Harrison, L. H., Chaisson, R. E. and Schechter, M. Estimating the Extent of Underreporting of Mortality Among HIV-Infected Individuals in Rio de Janeiro, Brazil. AIDS Research and Human Retroviruses 2011; 27:25-28. 10.1089/aid.2010.0089
385. Pinault, L., Brauer, M., Crouse, D. L., Weichenthal, S., Erickson, A., van Donkelaar, A., Martin, R. V., Charbonneau, S., Hystad, P., Brook, J. R., Tjepkema, M., Christidis, T., Menard, R., Robichaud, A. and Burnett, R. T. Diabetes Status and Susceptibility to the Effects of PM2.5 Exposure on Cardiovascular Mortality in a National Canadian Cohort. Epidemiology 2018; 29:784-794. 10.1097/ede.0000000000000908
386. Ramos, A. N., Jr., Matida, L. H., Hearst, N. and Heukelbach, J. Mortality in Brazilian children with HIV/AIDS: the role of non-AIDS-related conditions after highly active antiretroviral therapy introduction. AIDS Patient Care and STDs 2011; 25:713-8. 10.1089/apc.2011.0044

387. Redelings, M. D., Sorvillo, F. and Simon, P. A comparison of underlying cause and multiple causes of death: US vital statistics, 2000-2001. Epidemiology 2006; 17:100-3. 10.1097/01.ede.0000187177.96138.c6
388. Redelings, M. D., Wise, M. and Sorvillo, F. Using multiple cause-of-death data to investigate associations and causality between conditions listed on the death certificate. Am J Epidemiol 2007; 166:104-8. 10.1093/aje/kwm037
389. Rezende, E. M., Sampaio, I. B. and Ishitani, L. H. [Multiple causes of death due to non-communicable diseases: a multidimensional analysis]. Cad Saude Publica 2004; 20:1223-31. 10.1590/s0102-311x2004000500016
390. Rezende, E. M., Sampaio, B. M., Ishitani, L. H., Martins, E. F. and Vilella Lde, C. [Mortality of malnourished elderly in Belo Horizonte, Minas Gerais State, Brazil: a multidimensional analysis focusing on multiple causes of death]. Cad Saude Publica 2010; 26:1109-21. 10.1590/s0102-311x2010000600005
391. Riihimaki, M., Thomsen, H., Brandt, A., Sundquist, J. and Hemminki, K. What do prostate cancer patients die of? Oncologist 2011; 16(2):175-81.
392. Rockett, I. R., Wang, S., Lian, Y. and Stack, S. Suicide-associated comorbidity among US males and females: a multiple cause-of-death analysis. Inj Prev 2007; 13:311-5. 10.1136/ip.2007.015230
393. Rockett, I. R., Lian, Y., Stack, S., Ducatman, A. M. and Wang, S. Discrepant comorbidity between minority and white suicides: a national multiple cause-of-death analysis. BMC Psychiatry 2009; 9:10. 10.1186/1471-244x-9-10
394. Santo, A. H. Causes of death and mortality trends related to hemophilia in Brazil, 1999 to 2016. Hematol Transfus Cell Ther 2020; . 10.1016/j.htct.2020.02.003
395. Scholl, T., Stein, Z. and Hansen, H. Leukemia and other cancers, anomalies and infections as causes of death in Down's syndrome in the United States during 1976. Dev Med Child Neurol 1982; 24:817-29. 10.1111/j.1469-8749.1982.tb13702.x
396. Selik, R. M., Chu, S. Y. and Ward, J. W. Trends in infectious diseases and cancers among persons dying of HIV infection in the United States from 1987 to 1992. Ann Intern Med 1995; 123:933-6. 10.7326/0003-4819-123-12-199512150-00006
397. Selik, R. M., Byers Jr, R. H. and Dworkin, M. S. Trends in diseases reported on U.S. death certificates that mentioned HIV infection, 1987-1999. Journal of Acquired Immune Deficiency Syndromes 2002; 29:378-387. 10.1097/00126334-200204010-00009
398. Selik, R. M. and Lindegren, M. L. Changes in deaths reported with human immunodeficiency virus infection among United States children less than thirteen years old, 1987 through 1999. Pediatric Infectious Disease Journal 2003; 22:635-641. 10.1097/00006454-200307000-00013
399. Shah, N. A., Abate, M. A., Smith, M. J., Kaplan, J. A., Kraner, J. C. and Clay, D. J. Characteristics of alprazolam-related deaths compiled by a centralized state medical examiner. Am J Addict 2012; 21 Suppl 1:S27-34. 10.1111/j.1521-0391.2012.00298.x
400. Sosin, D. M., Sniezek, J. E. and Waxweiler, R. J. Trends in death associated with traumatic brain injury, 1979 through 1992. Success and failure. Jama 1995; 273:1778-80.
401. Thomas, B. M., Starr, J. M. and Whalley, L. J. Death certification in treated cases of presenile Alzheimer's disease and vascular dementia in Scotland. Age Ageing 1997; 26:401-406. 10.1093/ageing/26.5.401
402. Tolley, H. D., Manton, K. G. and Poss, S. S. A linear models application of competing risks to multiple causes of death. Biometrics 1978; 34:581-91.
403. Warner, M., Paulozzi, L. J., Nolte, K. B., Davis, G. G. and Nelson, L. S. State Variation in Certifying Manner of Death and Drugs Involved in Drug Intoxication Deaths. Academic Forensic Pathology 2013; 3:231-237. 10.23907/2013.029
404. Weiner, L. B., MT; McAvoy, GH et al. Use of multiple causes in the classification of deaths from cardiovascular-renal disease. American Journal of Public Health 1955; 14(2):100-5.

405. Whelton, P. K. and Goldblatt, P. An investigation of the relationship between stomach cancer and cerebrovascular disease: evidence for and against the salt hypothesis. Am J Epidemiol 1982; 115:418-27. 10.1093/oxfordjournals.aje.a113319
406. Breger, T. L., Edwards, J. K., Cole, S. R., Saag, M., Rebeiro, P. F., Moore, R. D. and Eron, J. J. Estimating a Set of Mortality Risk Functions with Multiple Contributing Causes of Death. Epidemiology 2020; 31:704-712. 10.1097/EDE.0000000000001225
407. Gonzalez, L. F., Jo, A. H. S. and Garcia, C. A. R. Weighted Mortality Method According to Multiple Causes of Death. Finlay 2019; 9:197-209.
408. Hassanzadeh, H. R., Sha, Y. andWang, M. D. DeepDeath: Learning to Predict the Underlying Cause of Death with Big Data. 2017 39th Annual International Conference of the IEEE Engineering in Medicine and Biology Society 2017; 3373-3376.
409. Jiang, H., Wu, H. and Wang, M. D. Causes of death in the United States, 1999 to 2014. 2017 IEEE EMBS International Conference on Biomedical and Health Informatics, BHI 2017 2017; 177-180. 10.1109/BHI.2017.7897234
