# Supplementary material for: Quantifying cause-related mortality in Australia, incorporating multiple causes: observed patterns, trends and practical considerations
Source: Int J Epidemiol. 2022 Aug 19;52(1):284–94. doi: 10.1093/ije/dyac167 (PMC9908048; doi:10.1093/ije/dyac167)
Supplement: dyac167_Supplementary_Data [file dyac167_supplementary_data.zip › dyac167_Supplementary_Data/Sup File2.pdf]

Supplementary File 2 – DETAILED METHODS

for Quantifying cause-related mortality in Australia incorporating multiple causes: observed patterns, trends, and practical considerations

Contents

1.1 Terminology for this analysis of multiple causes of death 2
1.2 Certification of causes of death in Australia 2
Doctor-certified deaths 3
Coroner-referred deaths 3
Cause of death data 4
Entity axis and record axis data 5
1.3 Standardised ratio of multiple to underlying (SRMU) 7
1.4 Weighting function for multiple cause weighting strategies 7
1.5 Key considerations for working with multiple causes of death 9
Descriptive multiple causes of death indicators 9
Specificity of the cause list 9
Ill-defined causes of death 10
Duplicate mentions of causes 11
Weighting strategy (if relevant) 11
Transparent reporting of methods 11
References 13
Cause list 14

1.1 Terminology for this analysis of multiple causes of death

Multiple causes of death: terminology

Medical certificate of cause of death (MCCD) is a standard international format for documenting the conditions that caused death, comprising:

> **Part I** refers to Part I of the MCCD and is used to record the temporal sequence of events including the initiating (**underlying**) cause, through to **intermediate** and the direct or **immediate** causes of death. Immediate and intermediate causes are typically complications or consequences of the underlying cause.
>
> **Part II** refers to Part II of the MCCD used for recording significant other **contributing** causes. These are not direct causes but causes that prevented the person from overcoming the illness. In principle the conditions reported here include diseases comorbid with, or antecedent to the underlying cause including previous history of a disease or significantly contributing risk factors.

Cause refers to a grouping of International Statistical Classification of Diseases and Related Health Problems 10th Revision (ICD-10)[1] codes.

Underlying cause (UC) refers to the condition or circumstances that initiated the morbid events leading to death and/or a useful intervention point for targeting public health and disease prevention strategies.

Entity axis data refers to causes of death data in a format that reflects causes as they were reported on the death certificate, including location and position.

Record axis data refers to the causes of death data in a format that reflects the roles (underlying or associated) of the causes according to the ICD-10 coding and processing rules.

Age-standardised rate (ASR) refers to the (sex-) and age-standardised rate. In this analysis rates were calculated based on the **underlying cause** (ASR_{UC}), **associated causes** (ASR_{AC}), **any mention** of a cause (ASR_{AM}) and **weighted multiple causes** (ASR_{W}).

Any mention (AM) refers to the consideration of each cause mentioned in the death record. In this analysis, these causes were selected from the **record axis**.

Contributing cause (CC) refers to the conditions involved in the death that were reported in Part II of the death certificate. In this analysis, these causes were selected from the **entity axis**.

Associated cause (AC) refers to the conditions involved in the death that were not the underlying cause; these include consequences and complications of diseases in the causal pathway as well as contributing causes (i.e. from **Part I** and **Part II** of the death certificate). In this analysis, these causes were selected from the **record axis**.

Standardised ratio of multiple to underlying cause (SRMU) measures the extent to which a cause is not the underlying cause. It is calculated as the rate ratio of the rate based on any mention to the rate underlying cause rate (ASR_{AM} / ASR_{UC}).

% Involvement as UC refers to the involvement of the cause of death as the underlying cause expressed as percent and is calculated as 1/SRMU x 100.

Weighted multiple causes refers to causes in each death that are weighted to reflect a proportional contribution to the death and such that the weights within each death sum to 1.0. Mortality indicators were assessed considering the following weighting strategies: the underlying cause weighted 1.0 (i.e. 100% responsible for causing death and reflecting the standard approach for measuring mortality); the underlying cause weighted 0.5 with the remaining causes in the death weighted equal portions of the remaining 0.5; and for sensitivity analysis, all causes weighted equally; and the underlying cause weighted twice the remaining causes with the latter weighted equally.

1.2 Certification of causes of death in Australia

The compilation of the Australian mortality data is a complex and highly structured process drawing together information from multiple jurisdictions and sources. Understanding the construction of the deaths data and processes for selecting the underlying cause, provides an

important framework for determining analytical techniques and drawing meaningful conclusions from the results.

The causes contributing to deaths in Australia are either certified by a doctor or referred to a coroner for further investigation. The documentation of the causes involved in the death differs considerably between these two practices; the implications for multiple cause analysis are described below.

Doctor-certified deaths

Doctors record the sequence of events leading to death in Part I of the Medical Certificate of Cause of Death (MCCD or death certificate), specifying at least an underlying cause and where relevant, intermediate causes and the immediate (direct) cause of death (Figure S2.1). All other significant diseases or conditions that contributed to the death but were not in the direct causal pathway, are recorded in Part II. These can include for example, comorbid chronic diseases, risk factors, and previous history of illness that prevented recovery or overcoming the morbid process leading to death.[2]

Medical Certificate of Cause of Death

				Time interval between onset and death
**PART I**<br>Disease or condition directly leading to death	a.	**immediate** (or direct) **cause of death**	due to (or as a consequence of) the next listed condition	
**Antecedent causes**<br>The conditions, if any, giving rise to the above cause, with the underlying condition stated last	b.			
	c.	**intermediate causes**	due to (or as a consequence of) the next listed condition	
	d.			
	e.	**underlying cause of death**	the cause listed last	
**PART II**<br>Other significant conditions contributing to the death, but not related to the disease or condition causing it		other **contributory casues**		

Figure S2.1: Layout of standard international form of Medical Certificate of Cause of Death

Coroner-referred deaths

In Australia deaths are referred to a coroner under a range of circumstances including when the person is unknown or died unexpectedly without known cause or in violent or unnatural circumstances or died during or resulting from anesthesia or in care or custody. Information about coroner-referred deaths is held in the National Coronial Information System (NCIS)

which comprises documents relating to the death including police, autopsy, toxicology, and coroners' reports. The Australian coronial system is jurisdictional, and information availability, timeliness and completeness differ across jurisdictions.

Approximately 12% of deaths annually are referred to a coroner. Approximately half of all coroner-referred deaths in Australia are due to external causes (accidents, assaults, and suicides) and the remainder are natural causes. Most external causes of death are certified by a coroner. For deaths due to external causes, the underlying cause reflects the mechanism and intent of the external circumstances, and the non-underlying causes reflect the type of injuries sustained, risk factors (for example, mental health conditions or chronic diseases as risk factors) or substances involved (for example, in poisoning-related deaths).

Cause of death data

All causes contributing to deaths are processed in accordance with World Health Organization (WHO) guidelines of the International Statistical Classification of Diseases and Related Health Problems 10th Revision (ICD-10).[3] At first, an ICD-10 code is assigned to all conditions as they appear on the MCCD. Then the ICD-10 coding rules are applied to select a single underlying cause of death. Considering the standpoint of prevention of death, the underlying cause represents a precipitating event for which a public health objective might prevent the death from occurring. The underlying cause is thus the disease or injury which initiated the train of morbid events leading directly to death, or the circumstances (e.g. accident or violence) leading to the fatal injury. The underlying cause is designated by WHO as the primary cause for tabulation of key health statistics (for example, leading causes of death) and consequently it is the underlying cause that underpins key statistics for public health policy and planning.

The assignment of ICD-10 codes and selection of the underlying cause for doctor-certified deaths is automated (using Iris software since 2013 and Medical Mortality Data System for earlier years).[4] The causal sequence reported in Part I of the MCCD is the primary focus of the highly complex ICD-10 coding guidelines for selecting the underlying cause of death. To construct the record axis, the coding process validates the reported sequence of events leading to death, identifying a single underlying cause. If a disease or condition is a logical consequence of another condition, (e.g. a secondary cancer) the prevention point is the precipitating cause (e.g. the primary cancer). This, in part, explains why some conditions are

rarely or never selected as the underlying cause. Where the sequence is implausible, or Part II indicates a more specific cause, all conditions on the death certificate (including from Part I and Part II) are re-examined to identify a single underlying cause.

Application of the coding guidelines ensure alignment with international best practice, enhancing, for example, the international comparability of mortality statistics. Importantly, the guidelines reduce the prospect of selecting a direct cause (for example, sepsis, pneumonia, heart, or respiratory failure) as the underlying cause, further facilitating identification of relevant health problems that cause from among a range of associated causes. Additionally, the guidelines help remove superfluous codes by linking relevant conditions reported in a death certificate to represent a more specific condition as the cause of death. For example, if both non-insulin-dependent diabetes mellitus (ICD-10 E11) and chronic kidney disease (N18) were reported on the death certificate, their joint involvement is indicated by one condition in the record axis as non-insulin-dependent diabetes mellitus with renal complications (E11.2), in place of the two separate causes of death included in the entity axis.

Entity axis and record axis data

The entity axis reflects the order and position of each cause as it was entered on the death certificate. While studies using death certificate data cannot substantiate the existence of causal relationships, the entity axis represents the doctors' (or other certifiers') perspective of the causal pathway by way of the location on the certificate (line and position) of the health event. This information is used to identify, for example, contributory causes reported in Part II of the death certificate. The causes of death in the entity axis are as per the death certificate; the ICD coding standards have not been applied to these causes. As such, in Australia the entity axis is not used for calculating leading causes of death and other public health indicators.

The record axis represents the causes of death after application of the automated ICD-10 coding rules. By application of the coding rules, one cause is selected as the underlying cause, and all other causes are presented in alphanumeric order of ICD-10 codes. The record axis data are used to derive key health indicators in Australia, facilitating international comparison of the underlying cause; they do not however, enable identification of the doctors' opinions

of the roles (immediate, intermediate, and contributory) of the causes. In the Australian data there can be up to 19 associated causes in addition to the underlying cause.

Figure S2.2 shows the structure of causes in the entity axis and the role of each in the record axis resulting from application of the coding rules.

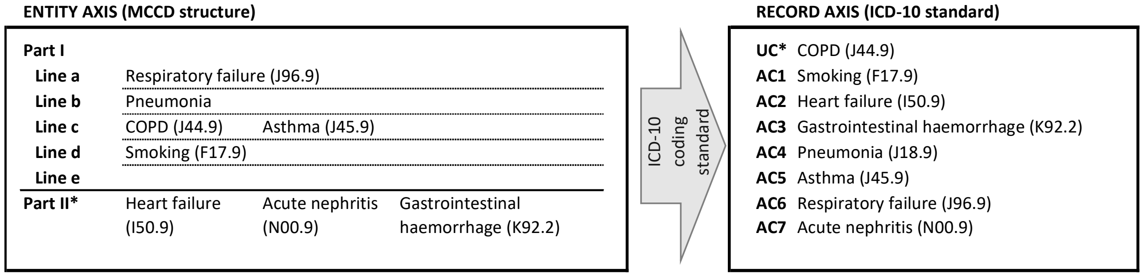

MCCD: Medical Certificate of Cause of Death; COPD: Chronic obstructive pulmonary disease
The lowest used line (in this case, d) represents the certifier's opinion of the underlying cause, the highest line (a), the immediate cause and in between (lines b and c), are intermediate causes.
Part II reflects the certifiers opinion of other significant conditions contributing to the death.
* For analysis, *contributing causes* include the underlying cause from the record axis and causes in Part II from the entity axis. Here the ICD-10 coding rules selected COPD as the underlying cause from the conditions and their positions in the entity axis.

Figure S2.2: Forms of cause of death data available for research

In this analysis, causes selected for weighting comprised the underlying cause from the record axis and contributing causes (i.e. causes reported in Part II of the death certificate) from the entity axis.

Changes over time in the underlying and non-underlying causes may be associated with changes in disease prevalence, certification practices, or coding guidelines.[4, 5] These drivers of change should be considered when assessing trends in multiple causes to understand whether changes are epidemiological or due to other factors.

Deaths and causes were assessed according to the reference year of death registration to facilitate identification of the impact of coding and administrative changes (usually implemented at a specific reference year). During the study period, the most significant changes to death registration processing were: the implementation of Iris coding software updating the previous automated coding of doctor-certified deaths; greater availability of information in the NCIS for coroner-certified deaths; and updates to WHO ICD-10 coding rules, all introduced in the reference year for 2013. The impact of updates to WHO ICD-10 coding rules on causes of death is described elsewhere.[4] Quality statements for the Australian causes of death data are reported elsewhere.[6]

1.3 Standardised ratio of multiple to underlying (SRMU)

The standardised ratio of multiple to underlying (SRMU) describes the extent to which a cause is selected as an associated cause compared to the underlying cause.[14, 15] This measure can be used to identify the degree to which causes contribute to death but are not measured in routine mortality statistics that use the underlying cause only.

The SRMU is calculated as a rate ratio, specifically the ratio of the age-standardised multiple cause (any mention) rate (ASR_{AM}) to the underlying cause rate (ASR_{UC}). The calculation is as follows

$$SRMU = \frac{ASR_{AM}}{ASR_{UC}} = \frac{\sum_x \frac{d_{AM,x}}{P_x} . w_{S_x}}{\sum_x \frac{d_{UC,x}}{P_x} . w_{S_x}}$$

where:

> $d_{AM,x}$ and $d_{UC,x}$ are the number of any mention and underlying cause deaths, respectively, in each age group x for the cause of interest;
>
> P_x is the population size (person-years) of age group x; and
>
> w_{S_x} is the standard population weight at age group x and is equivalent to the proportion of the total standard population (P_S) in age group x: ($w_{S_x} = P_{S_x} / \sum_x P_{S_x}$). P_S in this analysis is the Australian Estimated Resident Population for 30 June 2011.

The numerator (ASR_{AM}) incorporates the underlying cause and therefore the range of the SRMU is from 1.0 to infinity. An SRMU of 1.0 indicates that all mortality from the cause of interest is represented in the UC, SRMU=2.0 indicates approximately equal representation in the underlying and associated causes, and SRMU>2.0 suggests that the cause is more often an associated cause. An SRMU>3.0 for example, indicates that the involvement of the cause is at least twice as high as an associated compared to the underlying cause.

1.4 Weighting function for multiple cause weighting strategies

This section describes the application of existing multiple cause weighting strategies used to recalculate deaths and rates incorporating multiple cause.[16, 17]

Letting *i* be the index for *1 …D* deaths and *n* index the *1 … Ni* causes for each death. When *n=1*, the cause is the underlying cause and *n>1*, the cause is a contributing cause. Letting w_{ic} represents the cause of death weight attributed to cause *c* in death *i*. In all weighting functions the constraint is that weights applied in each death (w_{ic}) sum to 1. That is $\sum_{n=1}^{N_i} w_{ic} = 1$.

When considering only the underlying cause, that is, attributing 100% weight to the underlying cause and ignoring (or weighting as 0%) other causes in the death, the weighting function is:

$$w_{ic} = \begin{cases} 1 & n = 1 \\ 0 & n > 1 \end{cases}$$

The results reported in this analysis applied a 50% weight to the underlying cause with the remaining 50% apportioned equally across the contributing causes. The weighting function for this strategy (W) is:

$$w_{ic} = \begin{cases} 0.5 & n = 1 \\ \dfrac{0.5}{n_i - 1} & n > 1 \end{cases}$$

For sensitivity, we applied two additional weighting functions (see the results in Supplementary File 3 Additional tables). In the first (W1) all causes selected for weighting (the underlying and contributing) were weighted equally using the following function:

$$w_{ic} = \frac{1}{n_i}\ n \geq 1$$

And for the other alternative weighting strategy (W2), the underlying cause was weighted double, or twice that of each contributing cause. The weighting function is:

$$w_{ic} = \begin{cases} \dfrac{2}{n_i + 1} & n = 1 \\ \dfrac{1}{n_i + 1} & n > 1 \end{cases}$$

A hypothetical example of the weights ascribed to the underlying and contributing causes is under each of these strategies is shown in Figure S2.3.

Cause	Location	Weighting strategy			
		UC 100%	UC 50% (W)	Equal (W1)	UC double (W2)
Pneumonia	Part I (not underlying)	0	0	0	0
Lung cancer	Part I (underlying)	1	0.5	0.333	0.5
COPD	Contributing	0	0.25	0.333	0.25
Ischaemic heart disease	Contributing	0	0.25	0.333	0.25
	**Sum of weights**	**1**	**1**	**1**	**1**

W refers to the selected strategy used in the analysis; W1 and W2 to the alternative strategies for sensitivity analyses.

COPD: Chronic obstructive pulmonary disease.

Figure S2.3: Hypothetical example of application of multiple cause weighting to a death record

1.5 Key considerations for working with multiple causes of death

The methods used to categorise causes of death and to enumerate mentions of causes will impact the results of multiple cause analysis. Here we describe practical considerations in identifying and defining causes, non-contributing causes, and duplicate mentions of causes for population-level mortality indicators.

Descriptive multiple causes of death indicators

For calculating descriptive indicators (e.g. the average number of causes per death) maintain all mentions of causes in the record axis. This enables description of the extent to which multiple causes are used in certifying causes of death.

Specificity of the cause list

To better understand the role (underlying or associated) of causes of death and to facilitate weighting multiple causes, first develop an exhaustive (all ICD-10 codes) and mutually exclusive cause list to define causes by groups of ICD-10 codes. Such a list should be specific enough to identify causes not usually selected as the underlying cause. Very broad categories may result in important information being overlooked, especially for example, if there is variation in the role (underlying versus associated) between specific causes in the broader grouping of ICD-10 codes.

To ensure coverage of important fatal and usually non-fatal causes, there are mortality tabulation lists that could be consulted; these include the WHO condensed list (103 causes), the 2012 European shortlist (86 causes), a standard tabulation list for ranking leading underlying causes of death, as well as country-specific lists such as the Australian Burden of

Disease Study (216 causes) for identifying fatal and non-fatal health burden, and a specific list for categorising cardiovascular diseases.[3, 7-10]

Appropriate modifications to align the cause list to the research question and context should be made. For example, in this study, existing mortality tabulation lists were not suitable for adoption without modification as they either: were designed to tabulate leading underlying causes of death[3, 8, 9] (thus not identifying causes not usually selected as the underlying cause); did not suit the local Australian health context (for example, lacked specific causes such as asthma and COPD, each of which are monitored through national public health strategies in Australia),[11, 12] or comprised too many causes for this analysis.[10] In our analysis with expert input from the Investigators Group for the Multiple Causes of Death Project, we used the above causes lists as a starting point and identified 136 causes for multiple cause analysis. This provided a manageable number of causes for including causes not usually selected as underlying (see Cause list at end of document).

The level of specificity of a cause list should include the causes desired to be measured. The number of causes in the list will impact multiple causes statistics that are directly related to the number of causes per death (e.g. average number of causes and rates derived using the multiple-cause weighting method). Specific causes of interest should form their own group but note that a more detailed cause list can result in more causes per death compared with a less specific list. Similarly, the weights applied to multiple causes of death will be impacted. For example, a more detailed cause list results in a higher number of causes per death and there are more causes over which to apportion the weight of one death.

Ill-defined causes of death

Consider ignoring deaths and their causes with an ill-defined underlying cause as these do not provide further insight into patterns of causes of death.[13] Consider also excluding ill-defined causes from the non-underlying (associated or contributing, as relevant) causes. If they are maintained, with MC-weighting methods they will be considered as contributing to death. Alternatively, ill-defined causes could be redistributed to other causes using a redistribution method appropriate to multiple causes.

Duplicate mentions of causes

In mapping the ICD-10 codes for each cause in each death to the cause list, some ICD-10 codes can map to the same cause. This represents a duplicate mapping. The extent to which this occurs will depend on the level of detail of the list; a highly specific cause list will result in less duplication compared to one with causes defined by very broad ranges of ICD-10 codes.

If each cause is to be considered only once, ignore duplicate mentions of the underlying and other non-underlying causes. Maintaining these may over-estimate the contribution of diseases to mortality. Further, the mapping of two ICD-10 codes on the death certificate mapping to diabetes, for example, is not readily interpretable.

Initial sensitivity analyses comparing the SRMU with and without duplicate mentions showed differences for some causes (ischemic heart disease and cerebrovascular diseases) which could alter the interpretation of the SRMU.

Weighting strategy (if relevant)

For population level quantification of mortality: consider using the underlying cause as well as other contributing causes that were not part of the main morbid process (conditions in Part II of the death certificate). As the underlying cause of death is an internationally developed construct with a negotiated set of processes for identification, and remains critical for mortality statistics, it is reasonable to assign greater importance to the underlying cause. For example, a weight of 50% with the remaining 50% equally distributed among the causes in Part II.

For quantification of mortality from specific causes, consider using non-arbitrary weighting. Ideally, the development of non-arbitrary weights will be the object of an international consensus needing significant expert input using Delphi style consultation. If weighting of causes based on position on the death certificate is not relevant for the research objective, or these data are not available, consider all causes irrespective of their location on the death certificate, noting which causes were included for weighting.

Transparent reporting of methods

When reporting methods, include: the cause list used; details regarding the treatment of ill-defined causes and duplicate mentions; weighting strategies including the weights and type of data used (i.e. associated or selected types of causes).

In summary, the cause list adopted needs to be an exhaustive mapping of all possible causes of death, consist of mutually exclusive cause-groups, specific enough to identify causes that are not usually the underlying cause, and be suitable for monitoring causes of interest in the local health context and suitable for the research question. The exclusion or otherwise of ill-defined causes and duplicate mentions of causes will impact MC statistics. Further, complete and transparent reporting of analytical methods is necessary, and would facilitate comparison between studies.

References

1. World Health Organization. International statistical classification of diseases and related health problems - 10th revision. 2016.
2. Australian Bureau of Statistics. Cause of death certification. Canberra: Australia ABS; 2008.
3. World Health Organization. International statistical classification of diseases and related health problems - 10th revision. Geneva: Switzerland: WHO; 2016.
4. Australian Bureau of Statistics. *Causes of death, Australia, 2013.* 2015, [cited 4 June 2020]; Available from: https://www.abs.gov.au/AUSSTATS/abs@.nsf/Lookup/3303.0Main+Features12013?OpenDocument
5. Rao C, Adair T, Bain C, Doi SAR. Mortality from diabetic renal disease: A hidden epidemic. *Eur J Public Health* 2012; **22**: 280-4.
6. Australian Bureau of Statistics. *Causes of death, Australia 2017.* 2018, [cited 4 June 2020]; Available from: https://www.abs.gov.au/AUSSTATS/abs@.nsf/Lookup/3303.0Main+Features12017?OpenDocument
7. Joshy G, Korda R, Abhayaratna W, Soga K, Banks E. Categorising major cardiovascular disease hospitalisations from routinely collected data. *Public Health Research & Practice* 2015; **25**.
8. European Commission. *European Shortlist for Causes of Death*. 2012 [cited 30 September 2019]; Available from: https://ec.europa.eu/eurostat/ramon/nomenclatures/index.cfm?TargetUrl=LST_NOM_DTL&StrNom=COD_2012&StrLanguageCode=EN&IntPcKey=&StrLayoutCode=
9. Becker R, Silvi J, Ma Fat D, L'Hours A, Laurenti R. A method for tabulating leading causes of death. *Bulletin of the World Health Organization* 2006; **84**: 297-304.
10. Australian Institute of Health and Welfare. Australian Burden of Disease Study: methods and supplementary material 2015. Canberra, Australia: AIHW 2019.
11. National Asthma Council. National Asthma Strategy 2018. Melbourne: Australia: National Asthma Council; 2021.
12. Department of Health. *National Preventive Health Strategy*. 2021 [cited 11 May 2021]; Available from: https://www1.health.gov.au/internet/main/publishing.nsf/Content/national-preventive-health-strategy
13. Désesquelles A, Demuru E, Salvatore MA, et al. Mortality from Alzheimer's disease, Parkinson's disease, and dementias in France and Italy: a comparison using the multiple cause-of-death approach. *J Aging Health* 2014; **26**: 283-315.
14. Désesquelles A, Salvatore MA, Frova L, et al. Revisiting the mortality of France and Italy with the multiple-cause-of-death approach. *Demogr Res* 2010; **23**: 771-805.
15. Désesquelles A, Salvatore MA, Pappagallo M, et al. Analysing multiple causes of death: which methods for which data? An application to the cancer-related mortality in France and Italy. *Eur J Popul* 2012; **28**: 467-98.
16. Moreno-Betancur M, Sadaoui H, Piffaretti C, Rey G. Survival analysis with multiple causes of death extending the competing risks model. *Epidemiology* 2017; **28**: 12-9.
17. Piffaretti C, Moreno-Betancur M, Lamarche-Vadel A, Rey G. Quantifying cause-related mortality by weighting multiple causes of death. *Bull World Health Organ* 2016; **94**: 870-9.

Cause list

Cause name	ICD-10 codes
**Infectious diseases**	
Intestinal infections	A00–A09
Tuberculosis	A15–A19, B90
Septicaemia	A40–A41
Viral hepatitis	B15–B19, B942
HIV disease	B20–B24
Residual - infections	A20–A39, A42–A99, B00–B09, B25–B34, B35–B49, B50–B89, B91–B93, B940–B941, B948–B949, B95–B99
**Neoplasms**	
Oral cancers	C00–C14
Oesophagus cancer	C15
Stomach cancer	C16
Colorectal cancer	C18–C21, C260
Liver cancer	C22
Gallbladder cancer	C23–C24
Pancreatic cancer	C25
Larynx cancer	C32
Lung cancer	C33–C34
Malignant melanoma-skin	C43
Non-melanoma-skin	C44
Mesothelioma	C45
Breast cancer	C50
Cervical cancer	C53
Uterus cancer	C54–C55
Ovarian cancer	C56
Prostate cancer	C61
Kidney cancer	C64
Bladder cancer	C67
Brain cancer	C71
Thyroid cancer	C73
Cancer unknown primary	C76, C80, C97
Cancer secondary site	C77–C79
Hodgkin lymphoma	C81
Non-Hodgkin lymphomas	C82–C86
Other blood cancers	C88, C90–C96, D45–D46, D471, D473–D475
Residual - benign/in situ/uncertain neoplasms	D00–D44, D470, D472, D477–D479, D48
Residual - malignant neoplasms	C17, C261, C268, C269, C30–C31, C37–C39, C40–C41, C46–C49, C51–C52, C57–C58, C60, C62–C63, C65–C66, C68–C70, C72, C74–C75
**Blood diseases**	
Anaemias	D50–D64
Residual - blood diseases	D65–D89
**Endocrine disorders**	
Disorders of thyroid gland	E00–E07
Diabetes mellitus	E10–E14
Malnutrition	E40–E46
Obesity	E66
Amyloidosis	E85
Dehydration disorders	E86–E87
Metabolic disorders	E70–E84, E88–E90
Residual - endocrine	E15–E16, E20–E243, E248–E35, E50–E65, E67–E68

continued …

Cause list (continued)

Cause name	ICD-10 codes
**Mental & behavioural disorders**	
Alcohol induced diseases	E244, F10, G312, G621, G721, I426, K292, K70, K852, K860
Substance use disorders	F11–F19
Schizophrenia	F20–F29
Mood disorders	F30–F39
Residual - mental/behavioural	F04–F09, F40–F99
**Nervous system diseases**	
Inflammatory diseases - CNS	G00–G09
Systemic atrophies - CNS	G10–G14
Parkinson disease	G20
Dementia & Alzheimer disease	F00–F03, G30, G310, G318
Multiple sclerosis	G35
Epilepsy	G40, G41
Cerebral palsy	G80
Residual - nervous system	G21–G26, G311, G319, G32, G36–G37, G43–G44, G47, G50–G620, G622–G64, G70–G720, G722–G73, G81–G83, G90–G99 (G45–G46 – to TIA, G310, G318 to Dementia)
**Hearing and vision diseases**	H00–H95
**Cardiovascular diseases**	
Chronic rheumatic heart diseases	I05–I09
Hypertension	I10
Hypertensive diseases	I11–I15
Ischaemic heart disease	I20–I25
Pulmonary heart diseases	I26–I28
Non-rheumatic valve disorders	I34–I36
Atrial fibrillation	I48
Heart failure (specified)	I500, I501
Other heart diseases	I30–I33, I37–I39, I40–I41, I420–I425, I427–I43, I44–I45, I47, I49, I51, I52
Cerebrovascular disease	I60–I69
Artery diseases	I70–I79
Phlebitis & thrombophlebitis	I80
Transient cerebral ischaemic attack	G45–G46
Residual - cardiovascular	I00–I02, I81–I89, I950–I958, I96–I98
**Respiratory diseases**	
Influenza	J09–J11
Pneumonia	J12–J18
Other ALRI	J20–J22
COPD	J40–J44
Asthma	J45–J46
Bronchiectasis	J47
Pneumonitis	J69
Other interstitial respiratory diseases	J80–J84
Other diseases of pleura	J90–J94
Residual - respiratory	J00–J06, J30–J39, J60–J68, J70, J85–J86, J95, J961–J968, J97–J99

continued …

Cause list (continued)

Cause name	ICD-10 codes
**Digestive diseases**	
Diseases of oesophagus, stomach & duodenum	K20–K291, K293–K31
Other diseases of intestines	K55–K64
Diseases of peritoneum	K65–K67
Cirrhosis of the liver	K74
Other diseases of liver	K71–K73, K75–K77
Disorders of gallbladder, biliary tract & pancreas	K80–K851, K853–K859, K861–K87
Residual - digestive	K00–K14, K35–K38, K40–K46, K50–K52, K90–K93
**Skin diseases**	
Infections of skin	L00–L08
Residual - skin diseases	L10–L99
**Musculoskeletal conditions**	
Infectious arthropathies	M00–M03
Rheumatoid arthritis	M05–M06
Osteoarthritis	M15–M19
Systemic connective tissue disorders	M30–M36
Osteopathies & chondropathies	M80–M94
Residual - musculoskeletal	M07–M14, M20–M25, M40–M79, M95–M99
**Genitourinary diseases**	
Glomerular diseases	N00–N08
Renal tubulo-interstitial diseases	N10–N16
Renal failure	N17–N19
Urolithiasis	N20–N23
Hyperplasia of prostate	N40
Residual - genitourinary	N25–N29, N30–N39, N41–N51, N60–N99
**Maternal conditions**	O00–O99
**Perinatal conditions (including SIDS)**	P00–P284, P286–P96, R95
**Congenital conditions**	Q00–Q99
**Ill-defined causes**	I46, I509, I959, I99, J960, J969, P285, R00–R99 (excluding R95)
**Injuries**	S00–T99
Traumatic brain injury	S020, S021, S027–S029, S06, T902, T905
Spinal cord injury	S140, S141, S147, S240, S241, S247, S340, S341, S347, T060, T061, T093, T903, T913
Internal & crush injuries	S07, S17, S18, S224, S225, S25–S28, S297, S35–S37, S380, S381, S396, S397, S47, S57, S67, S77, S87, S97, T04, T065, T147, T914, T915
Poisoning - other substances	T36–T39, T407–T409, T41–T50, T52–T65, T940, T941, T96, T97
Poisoning - alcohol	T51
Poisoning - opioid	T400–T406
Hip fracture	S72, T931
Tibia & ankle fracture	S82
Humerus fracture	S422, S423, S424, S427
Other fractures	S022– S026, S028, S12, S220– S223, S228, S229, S32, S420–S421, S428–S429, S497, S52, S597, S620–S628, S697, S820, S92, T02, T08, T10, T12, T142, T911, T912, T921, T922, T932
Drowning/submersion injuries	T751
Dislocations	S030–S033, S131–S133, S231–S232, S331–S333, S430–S433, S530, S531, S630–S632, S730, S830, S831, S930, S931, S933, T03, T092, T112, T132, T143
Soft tissue injuries	S034–S035, S134–S136, S16, S230, S233–S235, S290, S335–S337, S390, S434–S437, S46, S532–S534, S56, S633–S637, S66, S731, S76, S832–S837, S86, S932, S934–S936, S96, T064, T095, T115, T135, T146
Burns	T20–T32, T95

continued …

Cause list (continued)

Cause name	ICD-10 codes
Medical-related injuries (consequences)	T80–T88, T983
Residual - injuries	S00, S01, S04, S05, S08–S11, S130, S142–S146, S15, S19, S20, S21, S242–S246, S298, S299, S30, S31, S330, S334, S342–S346, S348, S382, S383, S398, S399, S40, S41, S44, S45, S48, S498, S499, S50, S51, S54, S55, S58, S598, S599, S60, S61, S64, S65, S68, S698, S699, S70, S71, S74, S75, S78, S799, S80, S81, S84, S85, S88–S91, S94, S95, S98, S99, T00, T01, T05, T062, T063, T068, T07, T090, T091, T094, T096, T098, T099, T110, T111, T113, T114, T116, T118, T119, T130, T131, T133, T134, T136, T138, T139, T140, T141, T144, T145, T148, T149, T15–T19, T33–T35, T66–T75, T900, T901, T904, T908, T909, T910, T918–T920, T924, T928–T930,T933, T934, T936, T938, T939, T980, T981, T982 T980–T982
**External causes**	V00–Y98
RTI - motorcyclists	V203 –V209, V213–V219, V223–V229, V233–V239, V243–V249, V253–V259, V263–V269, V273–V279, V283–V289, V294–V299
RTI - motor vehicle occupants	V304–V309, V314–V319, V324–V329, V334–V339, V344–V349, V354–V359, V364–V369, V374–V379, V384–V389, V394–V399, V404–V409, V414–V419, V424–V429, V434–V439, V444–V449, V454–V459, V464–V469, V474–V479, V484–V489, V494–V499, V504–V509, V514–V519, V524–V529, V534–V539, V544–V549, V554–V559, V564–V569, V574–V579, V584–V589, V594–V599, V604–V609, V614–V619, V624–V629, V634–V639, V644–V649, V654–V659, V664–V669, V674–V679, V684–V689, V694–V699, V704–V709, V714–V719, V724–V729, V734–V739, V744–V749, V754–V759, V764–V769, V774–V779, V784–V789, V794–V799, V870–V879, V892, Y850
RTI - pedal cyclists	V103 –V109, V113–V119, V123–V129, V133–V139, V143–V149, V153–V159, V163–V169, V173–V179, V183–V189, V194–V199
RTI - pedestrians	V011, V019, V021, V029, V031, V039, V041, V049, V051, V059, V061, V069, V092, V093, V099
Accidental poisoning - alcohol	X45
Accidental poisoning - drugs	X42–X44
Falls	W00–W19
Drowning	V90, V92, W65–W74
Accidental threats to breathing	W75–W84
Suicide	X60–X84, Y870
Homicide & violence	X85–Y09, Y871
Medical-related injuries (external)	Y40–Y84, Y88
Residual - external causes	V010, V020, V030, V040, V050, V060, V090, V091, V100–V102, V110–V112, V120–V122, V130–V132, V140–V142, V150–V152, V160–V162, V170–V172, V180–V182, V190–V193, V200–V202, V210–V212, V220–V222, V230–V232, V240– V242, V250–V252, V260–V262, V270–V272, V280–V282, V290–V293, V300–V303, V310–V313, V320–V323, V330–V333, V340–V343, V350–V353, V360–V363, V370–V373, V380–V383, V390–V393, V400–V403, V410–V413, V420–V423, V430–V433, V440–V443, V450–V453, V460–V463, V470–V473, V480–V483, V490–V493, V500–V503, V510–V513, V520–V523, V530–V533, V540–V543, V550–V553, V560–V563, V570–V573, V580–V583, V590–V593, V600–V603, V610–V613, V620–V623, V630–V633, V640–V643, V650–V653, V660–V663, V670–V673, V680–V683, V690–V693, V700–V703, V710–V713, V720–V723, V730–V733, V740–V743, V750–V753, V760–V763, V770–V773, V780–V783, V790–V793, V80–V86, V88, V890, V891, V893, V899 , , V91, V93–V99, W20–W64, W85–W99, X00–X39, X40–X41, X46–X49, X50–X59, Y10–Y34, Y35–Y36, Y859, Y86, Y872, Y89, Y90–Y98

For description of ICD-10 codes, see reference above for World Health Organization (2016).

Four-character ICD-10 codes show here without the decimal point (e.g. B942 is B94.2)

CNS: Central nervous system; ALRI: Acute lower respiratory infection; COPD: Chronic obstructive pulmonary disease;

SIDS: Sudden infant death syndrome; RTI: Road traffic injury
