# Supplementary material for: Quantifying cause-related mortality in Australia, incorporating multiple causes: observed patterns, trends and practical considerations
Source: Int J Epidemiol. 2022 Aug 19;52(1):284–94. doi: 10.1093/ije/dyac167 (PMC9908048; doi:10.1093/ije/dyac167)
Supplement: dyac167_Supplementary_Data [file dyac167_supplementary_data.zip › dyac167_Supplementary_Data/Sup File3.pdf]

Supplementary File 3 – ADDITIONAL DATA TABLES

for Quantifying cause-related mortality in Australia incorporating multiple causes: observed patterns, trends, and practical considerations

Contents

Table S3.1: Descriptive measures for underlying and any-mention, SRMU and % involvement, by cause, Australia, 2015–2017 2

Table S3.2: Rates for underlying cause (ASR_{UC}) and weighting (ASR_W), percentage change and rate difference from ASR_{UC}, Australia, 2015–2017 5

Table S3.3: Age-standardised rates based on the selected and alternative multiple cause-weighting strategies, Australia, 2015–2017 8

Table S3.1: Descriptive measures for underlying and any-mention, SRMU and % involvement, by cause, Australia, 2015–2017

Cause name	No. deaths	% deaths	ASR_{UC}	No. mentions	% deaths	ASR_{AM}	SRMU	% UC
**Infectious diseases**	**8,322**	**1.8**	**10.730**	**39,800**	**8.5**	**51.539**	**4.8**	**20.8**
Intestinal infections	889	0.2	1.127	1,752	0.4	2.237	2.0	50.4
Tuberculosis	148	0.0	0.192	422	0.1	0.546	2.8	35.1
Septicaemia	4,889	1.0	6.243	28,162	6.0	36.215	5.8	17.2
Viral hepatitis	752	0.2	1.026	3,454	0.7	4.723	4.6	21.7
HIV disease	159	0.0	0.219	378	0.1	0.516	2.4	42.4
Residual-infectious	1,485	0.3	1.923	5,632	1.2	7.301	3.8	26.3
**Neoplasms**	**139,316**	**29.7**	**181.6**	**195,980**	**41.7**	**254.911**	**1.4**	**71.2**
Oral cancers	2,581	0.5	3.378	3,084	0.7	4.027	1.2	83.9
Oesophagus cancer	3,932	0.8	5.123	4,245	0.9	5.529	1.1	92.7
Stomach cancer	3,400	0.7	4.462	3,723	0.8	4.878	1.1	91.5
Colorectal cancer	16,339	3.5	21.277	19,925	4.2	25.854	1.2	82.3
Liver cancer	5,600	1.2	7.355	6,180	1.3	8.118	1.1	90.6
Gallbladder cancer	842	0.2	1.091	942	0.2	1.220	1.1	89.5
Pancreatic cancer	8,671	1.8	11.296	9,041	1.9	11.777	1.0	95.9
Larynx cancer	610	0.1	0.796	841	0.2	1.091	1.4	72.9
Lung cancer	25,142	5.4	32.75	27,392	5.8	35.666	1.1	91.8
Malignant melanoma-skin	4,178	0.9	5.478	5,363	1.1	7.001	1.3	78.2
Non-melanoma-skin	1,957	0.4	2.509	3,071	0.7	3.912	1.6	64.1
Mesothelioma	2,045	0.4	2.65	2,143	0.5	2.776	1.0	95.5
Breast cancer	8,901	1.9	11.711	11,975	2.5	15.603	1.3	75.1
Cervical cancer	719	0.2	0.965	854	0.2	1.138	1.2	84.8
Uterus cancer	1,531	0.3	1.988	1,808	0.4	2.345	1.2	84.8
Ovarian cancer	2,858	0.6	3.733	3,072	0.7	4.010	1.1	93.1
Prostate cancer	9,722	2.1	12.526	14,977	3.2	19.261	1.5	65.0
Kidney cancer	2,856	0.6	3.728	3,616	0.8	4.708	1.3	79.2
Bladder cancer	3,165	0.7	4.086	4,657	1.0	5.991	1.5	68.2
Brain cancer	4,245	0.9	5.646	4,399	0.9	5.846	1.0	96.6
Thyroid cancer	429	0.1	0.557	583	0.1	0.759	1.4	73.4
Cancer unknown primary	8,127	1.7	10.537	10,713	2.3	13.881	1.3	75.9
Hodgkin lymphoma	237	0.1	0.312	379	0.1	0.499	1.6	62.6
Non-Hodgkin lymphomas	4,526	1.0	5.894	6,082	1.3	7.908	1.3	74.5
Other blood cancers	10,549	2.2	13.696	14,934	3.2	19.339	1.4	70.8
Cancer secondary site	-	-	-	23,540	5.0	30.755	-	0.0
Residual-benign/in situ/uncertain neoplasms	1,577	0.3	2.047	3,057	0.7	3.957	1.9	51.7
Residual-malignant neoplasms	4,577	1.0	6.010	5,384	1.1	7.060	1.2	85.1
**Blood diseases**	**1,553**	**0.3**	**2.002**	**11,852**	**2.5**	**15.221**	**7.6**	**13.2**
Anaemias	787	0.2	0.998	7,525	1.6	9.547	9.6	10.4
Residual-blood diseases	766	0.2	1.005	4,327	0.9	5.673	5.6	17.7
**Endocrine disorders**	**20,276**	**4.3**	**26.176**	**85,628**	**18.2**	**110.534**	**4.2**	**23.7**
Disorders of thyroid gland	386	0.1	0.480	4,357	0.9	5.507	11.5	8.7
Diabetes	14,310	3.0	18.487	49,871	10.6	64.395	3.5	28.7
Malnutrition	314	0.1	0.398	2,426	0.5	3.121	7.8	12.7
Obesity	1,063	0.2	1.431	5,034	1.1	6.733	4.7	21.3
Amyloidosis	511	0.1	0.670	930	0.2	1.214	1.8	55.2
Dehydration disorders	928	0.2	1.156	7,551	1.6	9.600	8.3	12.0
Metabolic disorders	2,503	0.5	3.217	13,665	2.9	17.644	5.5	18.2
Residual-endocrine	261	0.1	0.337	1,794	0.4	2.321	6.9	14.5
**Mental & Behavioural disorders**	**5,206**	**1.1**	**6.919**	**45,256**	**9.6**	**60.554**	**8.8**	**11.4**
Alcohol induced diseases	3,741	0.8	5.049	10,488	2.2	14.220	2.8	35.5
Substance use disorders	123	0.0	0.171	12,112	2.6	16.261	95.0	1.1
Schizophrenia	158	0.0	0.206	2,895	0.6	3.883	18.8	5.3
Mood disorders	243	0.1	0.309	11,759	2.5	15.604	50.4	2.0
Residual-mental/behavioural	941	0.2	1.183	8,002	1.7	10.587	8.9	11.2

continued …

Table S3.1 (cont): Descriptive measures for underlying and any-mention, SRMU and % involvement, by cause, Australia, 2015–2017

Cause name	No. deaths	% deaths	ASR_{UC}	No. mentions	% deaths	ASR_{AM}	SRMU	% UC
**Nervous system diseases**	**53,729**	**11.4**	**67.964**	**110,668**	**23.6**	**140.733**	**2.1**	**48.3**
Inflammatory diseases - CNS	255	0.1	0.336	656	0.1	0.868	2.6	38.7
Systemic atrophies - CNS	2,476	0.5	3.247	2,907	0.6	3.807	1.2	85.3
Parkinson disease	5,307	1.1	6.9	10,223	2.2	13.291	1.9	51.9
Dementia & Alzheimer disease	40,853	8.7	51.063	78,187	16.6	98.122	1.9	52.0
Multiple sclerosis	536	0.1	0.709	835	0.2	1.1	1.6	64.5
Epilepsy	879	0.2	1.192	3,271	0.7	4.337	3.6	27.5
Cerebral palsy	321	0.1	0.441	552	0.1	0.755	1.7	58.4
Residual-nervous system	3,102	0.7	4.075	14,037	3.0	18.454	4.5	22.1
**Hearing & vision diseases**	**54**	**0.0**	**0.069**	**2,483**	**0.5**	**3.054**	**44.2**	**2.3**
**Cardiovascular diseases**	**129,111**	**27.5**	**163.745**	**395,146**	**84.1**	**502.47**	**3.1**	**32.6**
Chronic rheumatic heart diseases	1,157	0.2	1.492	2,382	0.5	3.076	2.1	48.5
Hypertension	1,735	0.4	2.154	60,298	12.8	76.39	35.5	2.8
Hypertensive diseases	5,126	1.1	6.393	6,896	1.5	8.71	1.4	73.4
Ischaemic heart disease	57,685	12.3	73.308	106,547	22.7	135.835	1.9	54.0
Pulmonary heart diseases	2,125	0.5	2.763	13,311	2.8	17.335	6.3	15.9
Non-rheumatic valve disorders	4,173	0.9	5.257	10,050	2.1	12.74	2.4	41.3
Atrial fibrillation	6,413	1.4	8.005	41,172	8.8	52.045	6.5	15.4
Heart failure (specified)	5,569	1.2	6.905	39,498	8.4	49.561	7.2	13.9
Other heart diseases	6,806	1.4	8.831	26,912	5.7	34.745	3.9	25.4
Cerebrovascular disease	31,521	6.7	39.914	60,415	12.9	76.7	1.9	52.0
Artery diseases	5,621	1.2	7.187	19,290	4.1	24.566	3.4	29.3
Phlebitis & thrombophlebitis	655	0.1	0.863	3,022	0.6	3.919	4.5	22.0
Transient cerebral ischaemic attacks	156	0.0	0.189	2,894	0.6	3.62	19.1	5.2
Residual-cardiovascular	369	0.1	0.484	2,459	0.5	3.228	6.7	15.0
**Respiratory diseases**	**45,187**	**9.6**	**57.555**	**166,655**	**35.5**	**212.822**	**3.7**	**27.0**
Influenza	2,019	0.4	2.533	2,585	0.6	3.255	1.3	77.8
Pneumonia	8,666	1.8	10.776	51,877	11.0	65.6	6.1	16.4
Other acute lower respiratory infections	1,275	0.3	1.576	7,344	1.6	9.262	5.9	17.0
COPD	21,938	4.7	28.216	48,397	10.3	62.296	2.2	45.3
Asthma	1,324	0.3	1.705	5,328	1.1	6.865	4.0	24.8
Bronchiectasis	1,176	0.3	1.509	2,749	0.6	3.53	2.3	42.8
Pneumonitis	2,324	0.5	2.898	22,625	4.8	28.868	10.0	10.0
Other interstitial respiratory diseases	4,181	0.9	5.416	13,069	2.8	16.792	3.1	32.3
Other diseases of pleura	265	0.1	0.334	3,566	0.8	4.601	13.8	7.3
Residual-respiratory	2,019	0.4	2.592	9,115	1.9	11.752	4.5	22.1
**Digestive diseases**	**14,745**	**3.1**	**18.893**	**53,224**	**11.3**	**69.079**	**3.7**	**27.3**
Diseases of oesophagus/stomach/duodenum	1,735	0.4	2.214	6,888	1.5	8.824	4.0	25.1
Other intestinal diseases	5,216	1.1	6.643	14,016	3.0	17.982	2.7	36.9
Diseases of peritoneum	225	0.0	0.292	1,776	0.4	2.32	7.9	12.6
Cirrhosis of the liver	1,401	0.3	1.856	5,696	1.2	7.588	4.1	24.5
Other diseases of liver	1,564	0.3	2.046	10,321	2.2	13.672	6.7	15.0
Disorders of gallbladder/biliary tract/pancreas	1,976	0.4	2.534	4,489	1.0	5.8	2.3	43.7
Residual-digestive	2,628	0.6	3.308	10,038	2.1	12.894	3.9	25.7
**Skin diseases**	**1,618**	**0.3**	**2.04**	**6,993**	**1.5**	**8.897**	**4.4**	**22.9**
Infections - skin	1,049	0.2	1.322	3,351	0.7	4.261	3.2	31.0
Residual-skin diseases	569	0.1	0.719	3,642	0.8	4.635	6.4	15.5
**Musculoskeletal conditions**	**4,095**	**0.9**	**5.197**	**25,696**	**5.5**	**32.514**	**6.3**	**16.0**
Infectious arthropathies	304	0.1	0.392	499	0.1	0.645	1.6	60.8
Rheumatoid arthritis	631	0.1	0.815	2,853	0.6	3.685	4.5	22.1
Osteoarthritis	393	0.1	0.475	5,886	1.3	7.300	15.4	6.5

continued …

Table S3.1 (cont): Descriptive measures for underlying and any-mention, SRMU and % involvement, by cause, Australia, 2015–2017

Cause name	No. deaths	% deaths	ASR_{UC}	No. mentions	% deaths	ASR_{AM}	SRMU	% UC
Systemic connective tissue disorders	826	0.2	1.081	2,334	0.5	3.023	2.8	35.7
Osteopathies	1,164	0.2	1.437	8,175	1.7	10.215	7.1	14.1
Residual-musculoskeletal	777	0.2	0.997	5,949	1.3	7.645	7.7	13.0
**Genitourinary diseases**	**10,715**	**2.3**	**13.515**	**75,880**	**16.2**	**96.687**	**7.2**	**14.0**
Glomerular diseases	285	0.1	0.371	1,692	0.4	2.178	5.9	17.0
Renal tubulo-interstitial diseases	381	0.1	0.497	1,481	0.3	1.925	3.9	25.8
Renal failure	6,708	1.4	8.462	57,767	12.3	73.647	8.7	11.5
Urolithiasis	128	0.0	0.166	332	0.1	0.430	2.6	38.6
Hyperplasia of prostate	190	0.0	0.238	944	0.2	1.197	5.0	19.9
Residual-genitourinary	3,023	0.6	3.780	13,664	2.9	17.309	4.6	21.8
**Maternal conditions**	**25**	**0.0**	**0.035**	**57**	**0.0**	**0.079**	**2.3**	**43.9**
**Perinatal conditions (incl SIDS)**	**1,775**	**0.4**	**2.462**	**2,186**	**0.5**	**3.031**	**1.2**	**81.2**
**Congenital conditions**	**1,814**	**0.4**	**2.479**	**2,899**	**0.6**	**3.943**	**1.6**	**62.9**
**Injuries - consequences**	**-**	**-**	**-**	**53,232**	**11.3**	**70.538**	**-**	**0.0**
Traumatic brain injury	-	-	-	4,346	0.9	5.645	-	0.0
Spinal cord injury	-	-	-	206	0.0	0.271	-	0.0
Internal & crush injuries	-	-	-	1,112	0.2	1.458	-	0.0
Poisoning-other substances	-	-	-	5,946	1.3	8.210	-	0.0
Hip fracture	-	-	-	6,489	1.4	8.092	-	0.0
Tibia and ankle fracture	-	-	-	287	0.1	0.370	-	0.0
Humerus fracture	-	-	-	469	0.1	0.590	-	0.0
Other fractures	-	-	-	4,774	1.0	5.983	-	0.0
Drowning/submersion injuries	-	-	-	1,017	0.2	1.383	-	0.0
Dislocations	-	-	-	102	0.0	0.132	-	0.0
Soft tissue injuries	-	-	-	48	0.0	0.063	-	0.0
Burns	-	-	-	403	0.1	0.549	-	0.0
Medical-related injuries (consequences)	-	-	-	3,264	0.7	4.262	-	0.0
Poisoning-alcohol	-	-	-	1,287	0.3	1.794	-	0.0
Poisoning-opioid	-	-	-	3,782	0.8	5.283	-	0.0
Residual-injuries	-	-	-	19,700	4.2	26.453	-	0.0
**External causes**	**32,117**	**6.8**	**42.982**	**57,157**	**12.2**	**75.347**	**1.8**	**57.0**
RTI: motorcyclists	635	0.1	0.882	637	0.1	0.885	1.0	99.7
RTI: motor vehicle occupants	2,458	0.5	3.374	2,662	0.6	3.652	1.1	92.4
RTI: pedal cyclists	96	0.0	0.131	100	0.0	0.136	1.0	95.9
RTI: pedestrians	450	0.1	0.609	461	0.1	0.624	1.0	97.6
Accidental poisoning-alcohol	262	0.1	0.363	946	0.2	1.319	3.6	27.5
Accidental poisoning-drugs	3,173	0.7	4.446	3,318	0.7	4.644	1.0	95.7
Falls	8,115	1.7	10.269	9,937	2.1	12.565	1.2	81.7
Drowning	655	0.1	0.893	698	0.1	0.951	1.1	93.9
Accidental threats to breathing	553	0.1	0.720	3,507	0.7	4.538	6.3	15.9
Suicide	9,132	1.9	12.617	9,145	1.9	12.634	1.0	99.9
Homicide & violence	718	0.2	0.996	757	0.2	1.050	1.1	94.9
Medical-related injuries (external)	482	0.1	0.627	15,171	3.2	19.652	31.3	3.2
Residual-external causes	5,388	1.1	7.054	9,818	2.1	12.697	1.8	55.6

SRMU: standardised ratio of multiple to underlying cause (ASR_{AM} / ASR_{UC}); UC: underlying cause; AM: any mention of a cause; ASR: age-standardised rate.

% deaths for number of mentions refers to the proportion of deaths having a mention of the cause

% UC refers to the involvement of the cause of death as the underlying cause expressed as percent and is calculated as the (1/SRMU)*100.

COPD: Chronic obstructive pulmonary disease; CNS: Central nervous system; SIDS: Sudden infant death syndrome; RTI: Road traffic injury.

Table S3.2: Rates for underlying cause (ASR_{UC}) and weighting (ASR_{W}), percentage change and rate difference from ASR_{UC}, Australia, 2015–2017

Cause name	ASR_{UC}	ASR_{W}	% change	Rate difference
**Infectious diseases**	**10.730**	**9.138**	**-14.83**	**-1.591**
Intestinal infections	1.127	0.883	-21.726	-0.245
Tuberculosis	0.192	0.211	10.115	0.019
Septicaemia	6.243	4.622	-25.974	-1.622
Viral hepatitis	1.026	1.351	31.733	0.326
HIV disease	0.219	0.259	18.689	0.041
Residual-infectious	1.923	1.812	-5.74	-0.11
**Neoplasms**	**181.600**	**160.014**	**-11.887**	**-21.586**
Oral cancers	3.378	2.950	-12.675	-0.428
Oesophagus cancer	5.123	4.396	-14.19	-0.727
Stomach cancer	4.462	3.880	-13.049	-0.582
Colorectal cancer	21.277	18.805	-11.62	-2.472
Liver cancer	7.355	6.216	-15.488	-1.139
Gallbladder cancer	1.091	0.927	-15.002	-0.164
Pancreatic cancer	11.296	9.670	-14.39	-1.625
Larynx cancer	0.796	0.698	-12.201	-0.097
Lung cancer	32.750	27.081	-17.31	-5.669
Malignant melanoma-skin	5.478	5.004	-8.642	-0.473
Non-melanoma-skin	2.509	2.273	-9.407	-0.236
Mesothelioma	2.650	2.287	-13.692	-0.363
Breast cancer	11.711	11.123	-5.019	-0.588
Cervical cancer	0.965	0.873	-9.599	-0.093
Uterus cancer	1.988	1.752	-11.849	-0.236
Ovarian cancer	3.733	3.306	-11.446	-0.427
Prostate cancer	12.526	11.718	-6.447	-0.807
Kidney cancer	3.728	3.277	-12.096	-0.451
Bladder cancer	4.086	3.628	-11.208	-0.458
Brain cancer	5.646	5.086	-9.907	-0.559
Thyroid cancer	0.557	0.511	-8.294	-0.046
Cancer unknown primary	10.537	8.795	-16.524	-1.741
Hodgkin lymphoma	0.312	0.291	-6.92	-0.022
Non-Hodgkin lymphomas	5.894	5.237	-11.154	-0.657
Other blood cancers	13.696	12.202	-10.91	-1.494
Cancer secondary site	0.000	0.811	.	0.811
Residual-benign/in situ/uncertain neoplasms	2.047	1.990	-2.817	-0.058
Residual-malignant neoplasms	6.010	5.226	-13.041	-0.784
**Blood diseases**	**2.002**	**2.993**	**49.484**	**0.991**
Anaemias	0.998	1.815	81.953	0.817
Residual-blood diseases	1.005	1.178	17.25	0.173
**Endocrine disorders**	**26.176**	**35.096**	**34.076**	**8.92**
Disorders of thyroid gland	0.480	1.399	191.479	0.919
Diabetes	18.487	23.991	29.771	5.504
Malnutrition	0.398	0.665	67.355	0.268
Obesity	1.431	2.227	55.626	0.796
Amyloidosis	0.670	0.572	-14.702	-0.099
Dehydration disorders	1.156	1.264	9.327	0.108
Metabolic disorders	3.217	4.406	36.949	1.189
Residual-endocrine	0.337	0.572	69.635	0.235

continued ...

Table S3.2 (cont): Rates for underlying cause (ASR_{UC}) and weighting (ASR_W), percentage change and rate difference from ASR_{UC}, Australia, 2015–2017

Cause name	ASR_{UC}	ASR_W	% change	Rate difference
**Mental & Behavioural disorders**	**6.919**	**16.553**	**139.225**	**9.633**
Alcohol induced diseases	5.049	5.979	18.421	0.930
Substance use disorders	0.171	2.985	1643.142	2.814
Schizophrenia	0.206	1.105	435.864	0.899
Mood disorders	0.309	3.867	1150.049	3.558
Residual-mental/behavioural	1.183	2.616	121.065	1.433
**Nervous system diseases**	**67.964**	**69.817**	**2.728**	**1.854**
Inflammatory diseases - CNS	0.336	0.291	-13.376	-0.045
Systemic atrophies - CNS	3.247	2.965	-8.681	-0.282
Parkinson disease	6.900	6.855	-0.652	-0.045
Dementia & Alzheimer disease	51.063	52.143	2.115	1.080
Multiple sclerosis	0.709	0.718	1.203	0.009
Epilepsy	1.192	1.570	31.715	0.378
Cerebral palsy	0.441	0.448	1.619	0.007
Residual-nervous system	4.075	4.827	18.451	0.752
**Hearing & vision diseases**	**0.069**	**0.644**	**830.937**	**0.575**
**Cardiovascular diseases**	**163.745**	**164.721**	**0.596**	**0.976**
Chronic rheumatic heart diseases	1.492	1.294	-13.259	-0.198
Hypertension	2.154	12.530	481.613	10.375
Hypertensive diseases	6.393	4.449	-30.408	-1.944
Ischaemic heart disease	73.308	63.455	-13.441	-9.853
Pulmonary heart diseases	2.763	3.668	32.75	0.905
Non-rheumatic valve disorders	5.257	4.965	-5.55	-0.292
Atrial fibrillation	8.005	12.591	57.278	4.585
Heart failure (specified)	6.905	9.208	33.353	2.303
Other heart diseases	8.831	9.040	2.366	0.209
Cerebrovascular disease	39.914	33.453	-16.187	-6.461
Artery diseases	7.187	7.635	6.242	0.449
Phlebitis & thrombophlebitis	0.863	1.137	31.794	0.274
Transient cerebral ischaemic attacks	0.189	0.642	239.102	0.453
Residual-cardiovascular	0.484	0.654	35.172	0.170
**Respiratory diseases**	**57.555**	**55.677**	**-3.263**	**-1.878**
Influenza	2.533	1.771	-30.087	-0.762
Pneumonia	10.776	9.697	-10.014	-1.079
Other acute lower respiratory infections	1.576	1.561	-0.975	-0.015
COPD	28.216	28.156	-0.212	-0.060
Asthma	1.705	2.207	29.457	0.502
Bronchiectasis	1.509	1.387	-8.096	-0.122
Pneumonitis	2.898	2.915	0.608	0.018
Other interstitial respiratory diseases	5.416	4.816	-11.092	-0.601
Other diseases of pleura	0.334	0.511	53.149	0.177
Residual-respiratory	2.592	2.656	2.481	0.064
**Digestive diseases**	**18.893**	**17.541**	**-7.155**	**-1.352**
Diseases - oesophagus/stomach/duodenum	2.214	2.491	12.514	0.277
Other intestinal diseases	6.643	5.446	-18.009	-1.196
Diseases of peritoneum	0.292	0.252	-13.639	-0.04
Cirrhosis of the liver	1.856	1.946	4.83	0.09
Other diseases of liver	2.046	2.238	9.35	0.191
Disorders - gallbladder/biliary tract/pancreas	2.534	2.136	-15.69	-0.398
Residual-digestive	3.308	3.032	-8.349	-0.276
**Skin diseases**	**2.040**	**2.201**	**7.850**	**0.160**
Infections - skin	1.322	1.168	-11.651	-0.154
Residual-skin diseases	0.719	1.033	43.707	0.314

continued …

Table S3.2 (cont): Rates for underlying cause (ASR_{UC}) and weighting (ASR_W), percentage change and rate difference from ASR_{UC}, Australia, 2015–2017

Cause name	ASR_{UC}	ASR_W	% change	Rate difference
**Musculoskeletal conditions**	**5.197**	**9.114**	**75.381**	**3.917**
Infectious arthropathies	0.392	0.301	-23.292	-0.091
Rheumatoid arthritis	0.815	1.275	56.408	0.460
Osteoarthritis	0.475	1.737	265.326	1.261
Systemic connective tissue disorders	1.081	1.240	14.782	0.160
Osteopathies	1.437	2.732	90.174	1.296
Residual-musculoskeletal	0.997	1.829	83.49	0.832
**Genitourinary diseases**	**13.515**	**20.952**	**55.024**	**7.437**
Glomerular diseases	0.371	0.637	71.491	0.265
Renal tubulo-interstitial diseases	0.497	0.458	-8.001	-0.040
Renal failure	8.462	15.369	81.622	6.907
Urolithiasis	0.166	0.144	-13.4	-0.022
Hyperplasia of prostate	0.238	0.327	37.37	0.089
Residual-genitourinary	3.780	4.017	6.28	0.237
**Maternal conditions**	**0.035**	**0.042**	**20.895**	**0.007**
**Perinatal conditions (incl SIDS)**	**2.462**	**2.477**	**0.616**	**0.015**
**Congenital conditions**	**2.479**	**2.440**	**-1.575**	**-0.039**
**Injuries - consequences**	**0.000**	**1.868**	**.**	**1.868**
Traumatic brain injury	0.000	0.125	.	0.125
Spinal cord injury	0.000	0.021	.	0.021
Internal & crush injuries	0.000	0.038	.	0.038
Poisoning-other substances	0.000	0.173	.	0.173
Hip fracture	0.000	0.434	.	0.434
Tibia and ankle fracture	0.000	0.026	.	0.026
Humerus fracture	0.000	0.043	.	0.043
Other fractures	0.000	0.410	.	0.410
Drowning/submersion injuries	0.000	0.003	.	0.003
Dislocations	0.000	0.008	.	0.008
Soft tissue injuries	0.000	0.006	.	0.006
Burns	0.000	0.004	.	0.004
Medical-related injuries (consequences)	0.000	0.059	.	0.059
Poisoning-alcohol	0.000	0.039	.	0.039
Poisoning-opioid	0.000	0.035	.	0.035
Residual-injuries	0.000	0.444	.	0.444
**External causes**	**42.982**	**33.074**	**-23.050**	**-9.907**
RTI: motorcyclists	0.882	0.801	-9.249	-0.082
RTI: motor vehicle occupants	3.374	3.021	-10.481	-0.354
RTI: pedal cyclists	0.131	0.122	-6.846	-0.009
RTI: pedestrians	0.609	0.521	-14.328	-0.087
Accidental poisoning-alcohol	0.363	0.249	-31.522	-0.114
Accidental poisoning-drugs	4.446	2.730	-38.601	-1.716
Falls	10.269	7.636	-25.644	-2.633
Drowning	0.893	0.752	-15.807	-0.141
Accidental threats to breathing	0.720	0.639	-11.309	-0.081
Suicide	12.617	8.085	-35.916	-4.531
Homicide & violence	0.996	0.909	-8.703	-0.087
Medical-related injuries (external)	0.627	1.604	155.753	0.977
Residual-external causes	7.054	6.006	-14.854	-1.048

Age-standardised rate (ASR) refers to the age-standardised rate based on the underlying causes (ASR_{UC}) and weighted multiple causes (ASR_W).

% change refers to the change in rates of ASR_W relative to ASR_{UC} expressed as a percent (negative numbers indicate a decrease in rates).

Rate difference is calculated based as ASR_{UC} minus ASR_W.

COPD: Chronic obstructive pulmonary disease; CNS: Central nervous system; SIDS: Sudden infant death syndrome; RTI: Road traffic injury.

Table S3.3: Age-standardised rates based on the selected and alternative multiple cause-weighting strategies, Australia, 2015–2017

Cause name	ASR_W	ASR_{W1}	ASR_{W2}
**Infectious diseases**	**9.138**	**8.660**	**9.160**
Intestinal infections	0.883	0.810	0.880
Tuberculosis	0.211	0.210	0.210
Septicaemia	4.622	4.180	4.700
Viral hepatitis	1.351	1.450	1.340
HIV disease	0.259	0.260	0.250
Residual-infectious	1.812	1.750	1.780
**Neoplasms**	**160.014**	**155.220**	**162.160**
Oral cancers	2.950	2.860	3.000
Oesophagus cancer	4.396	4.260	4.490
Stomach cancer	3.880	3.750	3.940
Colorectal cancer	18.805	18.270	19.070
Liver cancer	6.216	5.970	6.340
Gallbladder cancer	0.927	0.890	0.940
Pancreatic cancer	9.670	9.320	9.850
Larynx cancer	0.698	0.670	0.700
Lung cancer	27.081	25.910	27.740
Malignant melanoma-skin	5.004	4.910	5.070
Non-melanoma-skin	2.273	2.210	2.290
Mesothelioma	2.287	2.210	2.320
Breast cancer	11.123	10.980	11.170
Cervical cancer	0.873	0.860	0.890
Uterus cancer	1.752	1.700	1.770
Ovarian cancer	3.306	3.210	3.350
Prostate cancer	11.718	11.500	11.760
Kidney cancer	3.277	3.180	3.330
Bladder cancer	3.628	3.520	3.660
Brain cancer	5.086	4.980	5.160
Thyroid cancer	0.511	0.500	0.520
Cancer unknown primary	8.795	8.420	8.980
Hodgkin lymphoma	0.291	0.280	0.290
Non-Hodgkin lymphomas	5.237	5.060	5.270
Other blood cancers	12.202	11.830	12.300
Cancer secondary site	0.811	0.940	0.670
Residual-benign/in situ/uncertain neoplasms	1.990	1.970	1.990
Residual-malignant neoplasms	5.226	5.060	5.310
**Blood diseases**	**2.993**	**3.220**	**2.910**
Anaemias	1.815	2.010	1.750
Residual-blood diseases	1.178	1.210	1.150
**Endocrine disorders**	**35.096**	**37.540**	**34.700**
Disorders of thyroid gland	1.399	1.660	1.370
Diabetes	23.991	25.330	23.560
Malnutrition	0.665	0.720	0.630
Obesity	2.227	2.420	2.170
Amyloidosis	0.572	0.550	0.580
Dehydration disorders	1.264	1.270	1.230
Metabolic disorders	4.406	4.960	4.610
Residual-endocrine	0.572	0.630	0.550

continued …

Table S3.3 (cont): Age-standardised rates based on the selected and alternative multiple cause-weighting strategies, Australia, 2015–2017

Cause name	ASR_W	ASR_{W1}	ASR_{W2}
**Mental & Behavioural disorders**	**16.553**	**18.750**	**15.660**
Alcohol induced diseases	5.979	6.170	5.870
Substance use disorders	2.985	3.590	2.690
Schizophrenia	1.105	1.290	1.000
Mood disorders	3.867	4.690	3.550
Residual-mental/behavioural	2.616	3.010	2.550
**Nervous system diseases**	**69.817**	**69.150**	**68.490**
Inflammatory diseases - CNS	0.291	0.280	0.300
Systemic atrophies - CNS	2.965	2.900	3.000
Parkinson disease	6.855	6.810	6.820
Dementia & Alzheimer disease	52.143	51.310	50.900
Multiple sclerosis	0.718	0.710	0.710
Epilepsy	1.570	1.650	1.520
Cerebral palsy	0.448	0.450	0.440
Residual-nervous system	4.827	5.040	4.800
**Hearing & vision diseases**	**0.644**	**0.810**	**0.630**
**Cardiovascular diseases**	**164.721**	**165.830**	**165.580**
Chronic rheumatic heart diseases	1.294	1.260	1.320
Hypertension	12.530	15.280	11.970
Hypertensive diseases	4.449	4.000	4.630
Ischaemic heart disease	63.455	61.080	64.240
Pulmonary heart diseases	3.668	3.830	3.530
Non-rheumatic valve disorders	4.965	4.910	5.010
Atrial fibrillation	12.591	13.860	12.400
Heart failure (specified)	9.208	9.890	9.160
Other heart diseases	9.040	9.130	9.070
Cerebrovascular disease	33.453	32.120	34.200
Artery diseases	7.635	7.790	7.650
Phlebitis & thrombophlebitis	1.137	1.200	1.110
Transient cerebral ischaemic attacks	0.642	0.780	0.640
Residual-cardiovascular	0.654	0.690	0.640
**Respiratory diseases**	**55.677**	**55.000**	**55.580**
Influenza	1.771	1.580	1.830
Pneumonia	9.697	9.450	9.800
Other acute lower respiratory infections	1.561	1.550	1.550
COPD	28.156	27.900	27.900
Asthma	2.207	2.370	2.220
Bronchiectasis	1.387	1.360	1.400
Pneumonitis	2.915	2.900	2.890
Other interstitial respiratory diseases	4.816	4.670	4.870
Other diseases of pleura	0.511	0.540	0.490
Residual-respiratory	2.656	2.660	2.640
**Digestive diseases**	**17.541**	**17.190**	**17.620**
Diseases - oesophagus/stomach/duodenum	2.491	2.630	2.550
Other intestinal diseases	5.446	5.110	5.490
Diseases of peritoneum	0.252	0.240	0.250
Cirrhosis of the liver	1.946	1.980	1.960
Other diseases of liver	2.238	2.280	2.220
Disorders - gallbladder/biliary tract/pancreas	2.136	2.010	2.130
Residual-digestive	3.032	2.930	3.010

continued …

Table S3.3 (cont): Age-standardised rates based on the selected and alternative multiple cause-weighting strategies, Australia, 2015–2017

Cause name	ASR_W	ASR_{W1}	ASR_{W2}
**Skin diseases**	**2.201**	**2.190**	**2.130**
Infections - skin	1.168	1.100	1.140
Residual-skin diseases	1.033	1.090	0.990
**Musculoskeletal conditions**	**9.114**	**10.150**	**8.910**
Infectious arthropathies	0.301	0.280	0.300
Rheumatoid arthritis	1.275	1.360	1.210
Osteoarthritis	1.737	2.100	1.700
Systemic connective tissue disorders	1.240	1.270	1.220
Osteopathies	2.732	3.060	2.650
Residual-musculoskeletal	1.829	2.080	1.830
**Genitourinary diseases**	**20.952**	**22.500**	**20.100**
Glomerular diseases	0.637	0.690	0.600
Renal tubulo-interstitial diseases	0.458	0.440	0.460
Renal failure	15.369	16.820	14.590
Urolithiasis	0.144	0.140	0.150
Hyperplasia of prostate	0.327	0.360	0.330
Residual-genitourinary	4.017	4.050	3.970
**Maternal conditions**	**0.042**	**0.040**	**0.040**
**Perinatal conditions (incl SIDS)**	**2.477**	**2.480**	**2.470**
**Congenital conditions**	**2.440**	**2.420**	**2.430**
**Injuries - consequences**	**1.868**	**2.620**	**2.050**
Traumatic brain injury	0.125	0.170	0.130
Spinal cord injury	0.021	0.030	0.020
Internal & crush injuries	0.038	0.050	0.040
Poisoning-other substances	0.173	0.240	0.180
Hip fracture	0.434	0.630	0.490
Tibia and ankle fracture	0.026	0.040	0.030
Humerus fracture	0.043	0.060	0.050
Other fractures	0.410	0.590	0.470
Drowning/submersion injuries	0.003	0.000	0.000
Dislocations	0.008	0.010	0.010
Soft tissue injuries	0.006	0.010	0.010
Burns	0.004	0.000	0.000
Medical-related injuries (consequences)	0.059	0.080	0.070
Poisoning-alcohol	0.039	0.050	0.040
Poisoning-opioid	0.035	0.050	0.040
Residual-injuries	0.444	0.600	0.460
**External causes**	**33.074**	**30.590**	**33.720**
RTI: motorcyclists	0.801	0.790	0.820
RTI: motor vehicle occupants	3.021	2.970	3.090
RTI: pedal cyclists	0.122	0.120	0.120
RTI: pedestrians	0.521	0.510	0.530
Accidental poisoning-alcohol	0.249	0.220	0.250
Accidental poisoning-drugs	2.730	2.140	2.670
Falls	7.636	6.850	7.660
Drowning	0.752	0.720	0.770
Accidental threats to breathing	0.639	0.630	0.660
Suicide	8.085	7.000	8.450
Homicide & violence	0.909	0.900	0.930
Medical-related injuries (external)	1.604	1.910	1.600
Residual-external causes	6.006	5.830	6.170

ASR: Age-standardised rate.

Weighting strategy W refers to the weighting the underlying cause 50% with the remaining 50% apportioned equally to contributing causes; W1 to weighting all causes an equal amount, and W2 to weighting the underlying cause twice the weight of each other cause (see also Supplementary File 2 Detailed methods).

COPD: Chronic obstructive pulmonary disease; CNS: Central nervous system; SIDS: Sudden infant death syndrome; RTI: Road traffic injury.
